# Heimionones A–E, New Sesquiterpenoids Produced by *Heimiomyces* sp., a Basidiomycete Collected in Africa

**DOI:** 10.3390/molecules28093723

**Published:** 2023-04-26

**Authors:** Sebastian Pfütze, Atchara Khamsim, Frank Surup, Cony Decock, Josphat C. Matasyoh, Marc Stadler

**Affiliations:** 1 Department of Microbial Drugs, Helmholtz Center for Infection Research (HZI), German Center for Infection Research (DZIF), Partner Site Hannover/Braunschweig, Inhoffenstrasse 7, 38124 Braunschweig, Germany; sebastian.pfuetze@helmholtz-hzi.de (S.P.); frank.surup@helmholtz-hzi.de (F.S.); 2 Institute of Microbiology, Technische Universität Braunschweig, Spielmannstraße 7, 38106 Braunschweig, Germany; 3 Earth and Life Institute, Mycothéque de l’ Universite Catholique de Louvain (BCCM/MUCL), Place Croix du Sud 3, B-1348 Louvain-la-Neuve, Belgium; cony.decock@uclouvain.be; 4 Department of Chemistry, Egerton University, P.O. Box 536, Njoro 20115, Kenya

**Keywords:** *Heimiomyces* sp., heimionone, chemical diversity

## Abstract

With heimionones A–E (**1**–**5**), five new terpenoids were isolated from submerged cultures of *Heimiomyces* sp. in addition to the previously described compounds hispidin, hypholomin B, and heimiomycins A and B. Planar structures of the metabolites were elucidated by 1D and 2D NMR in addition to HRESIMS data. While ROESY data assigned relative configurations, absolute configurations were determined by the synthesis of MTPA esters of **1**, **3**, and **5**. The [6.3.0] undecane core structure of compounds **3**–**5** is of the asteriscane-type, however, the scaffold of **1** and **2** with their bicyclo [5.3.0] decane core and germinal methyl substitution is, to our knowledge, unprecedented. Together with several new compounds that were previously isolated from solid cultures of this strain, *Heimiomyces* sp. showed an exceptionally high chemical diversity of its secondary metabolite profile.

## 1. Introduction

In the ever-growing effort to isolate, identify, and characterize novel natural products, the kingdom of Fungi serves as a well-known, reliable, and nearly inexhaustible source. Ongoing developments in numerous modern techniques significantly support the identification of hitherto unknown secondary metabolites, leading to a continuously rising number of newly discovered molecules. With regard to the ca. 35.000 species comprising [1] and therefore the second-largest phylum of the kingdom Fungi, the Basidiomycota have proven to produce chemically very diverse secondary-metabolite profiles next to their biological diversity [2]. While there are Basidiomycota that mainly produce several congeners of the same compound family, such as members of the genus *Armillaria*, which are known for their large amount of structurally closely related sesquiterpenoids aryl esters [3], there are also genera that show a high chemical diversity within their secondary metabolite profile concerning the presence of different core structures. *Heimiomyces* sp. (MUCL 56078) is an example of the latter since our previous studies already led to the isolation and identification of heimiocalamenes C–E (**8**–**10**) and heimiomycins A–C (**11**–**13**) [4], as well as bis-heimiomycins A–D (**14**–**17**), heimiomycins D–E (**18**–**19**) and heimiocalamenes A–B (**20**–**21**) [5], showing the enormous potential of this specimen to produce chemically very diverse secondary metabolites. Intriguingly, the secondary metabolite pattern of *Heimiomyces* sp. even changed drastically after switching the cultivation conditions from solid rice cultures to submerged cultures.

We herein present the isolation, structural elucidation, and biological evaluation of the new terpenoids heimionones A–E (**1**–**5**) with uncommon bicyclo [5.3.0] decane and [6.3.0] undecane core structures, respectively, that were isolated from submerged cultures of *Heimiomyces* sp. alongside the previously described heimiomycins A–B (**11**–**12**) [4], as well as hispidin and hypholomin B (**6**–**7**) [6,7].

## 2. Results and Discussion

### 2.1. Isolation and Structure Elucidation of Metabolites from Heimiomyces sp. (Figure 1, Figure 2 and Figure 3) 

Heimionone A (**1**) was isolated as a yellow oil from the mycelial extracts of liquid cultures. Based on an HRESIMS analysis, its molecular formula was assigned as C_22_H_28_O_5_ according to the molecular ion cluster at *m*/*z* 373.2012 [M + H]^+^ (calcd. for C_22_H_29_O_5_ 373.2023) indicating nine degrees of unsaturation. ^1^H NMR and HSQC data (Table S6) led to the identification of six methyls at δ_H_ 1.02 (s, H_3_-12), 1.13 (s, H_3_-11), 1.40 (d, *J* = 6.3 Hz, H_3_-15), 1.77 (s, H_3_-7′), 1.82 (d, *J* = 7.0 Hz, H_3_-6′), and 2.31 (s, H_3_-13), three oxymethines at δ_H_ 4.70 (q, H-14), 5.14 (s, H-9), and 6.12 (s, H-1), and five olefinic methines at δ_H_ 5.81 (d, *J* = 15.7 Hz, H-2′), 6.07 (q, H-5′), 7.35 (d, *J* = 15.7 Hz, H-3′), 7.56 (d, *J* = 9.8 Hz, H-4), and 7.69 (d, *J* = 9.8 Hz, H-5). The ^13^C and HMBC NMR data (Table S6) revealed the presence of 22 carbon resonances, including two carbonyl carbons (δ_C_ 187.5, C-7; 168.6, C-1′); ten sp^2^-hybridized carbons, comprising five nonprotonated carbons (δ_C_ 153.0, C-2; 152.4, C-3; 151.7, C-6; 146.1, C-8; 135.3, C-4′) and five methines (δ_C_ 152.6, C-3′; 139.2, C-5′; 138.6, C-5; 131.8, C-4; 115.1, C-1′); one quaternary carbon (δ_C_ 45.5, C-10); three methines (δ_C_ 84.2, C-9; 83.5, C-1; 67.8, C-14); and six methyl carbons (δ_C_ 26.1, C-15; 22.3, C-13; 20.7, C-12; 20.5, C-11; 14.8, C-6′; 11.9 C-7′). The carbonyl and sp^2^-hybridized carbons accounted for seven degrees of unsaturation, suggesting two rings in the scaffold of heimionone A (**1**). By analyzing the ^1^H-^1^H COSY data, the first spin system was given due to correlations between H_3_-13, H-5, H-4, H-14, and H_3_-15. Correlations between H_3_-11/H_3_-12, H_2_-9, and H-1 led to the identification of the second spin system. HMBC correlations from H_3_-13 to C-4/C-5/C-6/C-7/C-8 and H-4 to C-2/C-3/C-5/C-6/C-7/C-8 revealed a cyclohepta-3,5,8-triene-7-one ring. The 14-hydroxyethyl moiety was deduced from the HMBC correlations of H_3_-15 to C-3/C-14. Further HMBC correlations from H_3_-11 and H_3_-12 to C-1/C-9/C-10, from H-9 to C-2/C-8/C-10/C-11, and from H-1 to C-2/C-8/C-9/C-10/C-11 led to the identification of a dimethylcyclopentane substructure that was fused to the cyclohepta-3,5,8-triene-7-one ring across C-2 and C-8. HMBC correlations from H_3_-6′ to C-3′/C-4′/C-5′/C-7′ and from H-3′ to C-1′/C-2′/C-4′/C-5′/C-7′ revealed the presence of a 4′-methylhexa-2′,4′-dienoic acid partial structure that was fused to C-1 according to an HMBC correlation from H-1 to C-1′. The *E* ∆^2′,3′^ configuration was assigned according to the large coupling constants (*J* = 15.7 Hz) between the olefinic methines H-2′ and H-3′, while the ∆^4′,5′^ configuration was assigned as *E* due to the ROESY correlation between H-3′ and H-5′. Furthermore, this structural elucidation was supported by the comparison of the ^1^H and ^13^C data to the ones of daldinin F (Figure S17 and Table S11), which was reported to carry the same side chain as heiminone A [8]. The relative configuration of the bicyclic core was obtained by the evaluation of ROE data. Due to the key correlation between H-1 and H_3_-12, these protons were arbitrarily assigned to the α face of the molecule. A correlation between H-9β/H-11 indicated an β orientation of these protons. Finally, the absolute configuration was determined by Mosher’s method. The derivatization of heimionone A (**1**) to its corresponding *R*- and *S*-MTPA esters at position C-9 and C-14 was done with *S*- and *R*-MTPA chloride. The pattern of ∆δ*^S^*^,*R*^ chemical shifts (see Figure 3) with positive values of H-4, H-5, H_3_-11, and H_3_-12, and negative ones of H_3_-13 and H_3_-15 determined the 1*S*,9*R*,14*S* absolute configuration.

**Figure 1:**
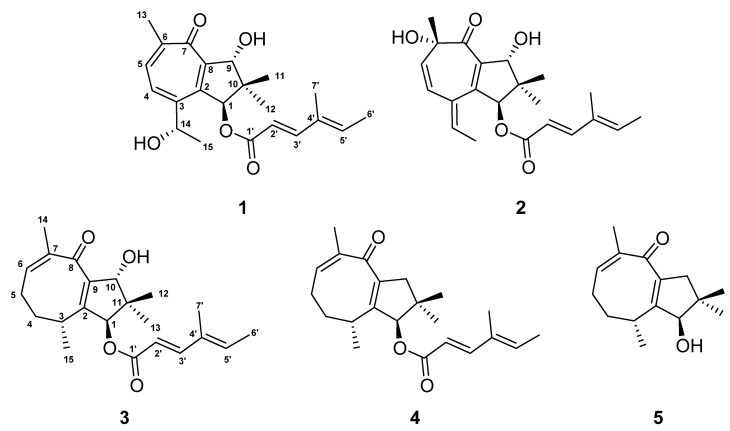
Chemical structures of heimionone A–E (**1**–**5**) isolated from liquid cultures of *Heimiomyces* sp.

**Figure 2:**
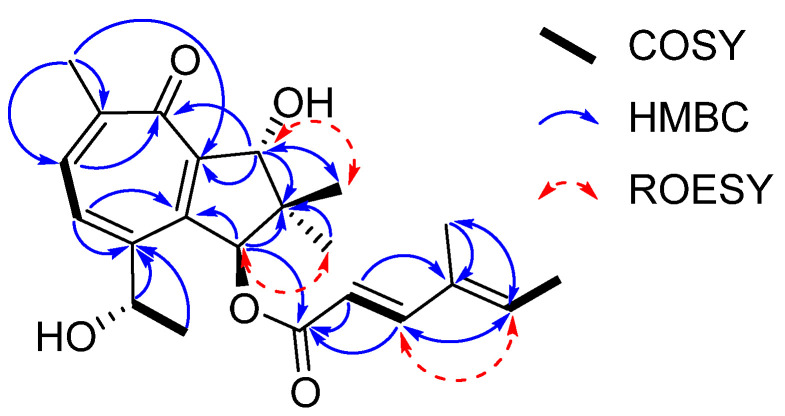
Key COSY, HMBC, and ROESY correlations of heimionone A (**1**).

**Figure 3:**
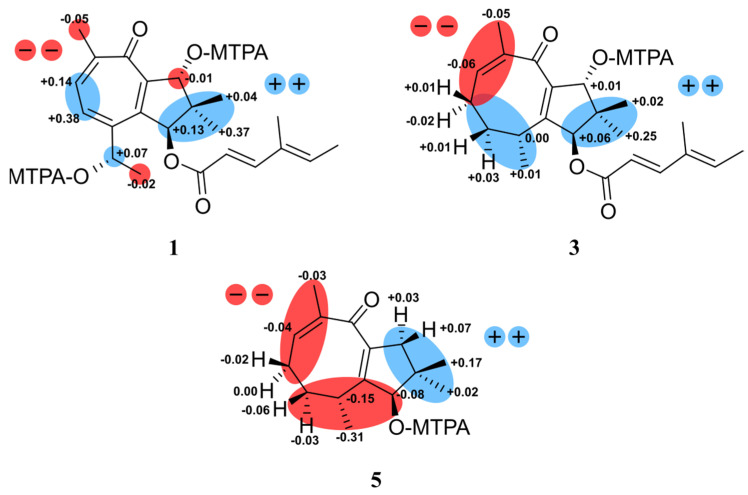
The Δδ*^SR^* values of (*S*)/(*R*) MTPA esters obtained from heimionone A (**1**) diagnostic for 1*S*,9*R*,14*S*; heimionone C (**3**) diagnostic for 1*S*,3*R*,10*R* and heimionone E (**5**) diagnostic for 1*R*,3*R*.

Heimionone B (**2**), which is closely related to **1**, was obtained as a yellow oil from the supernatant extracts of liquid cultures. Its HRESIMS data indicated the same molecular formula (C_22_H_28_O_5_) as obtained for **1**. ^1^H and ^13^C data showed high similarities to **1**. They differ by the relocation of the hydroxy function from C-14 for **1** to C-6 (δ_C_ 78.7) for **2**, whereas the oxymethine at C-14 was replaced by an olefinic methine (δ_H_ 6.16, δ_C_ 141.6). The cyclohepta-4,8-diene-7-one scaffold was confirmed by HMBC correlations of H_3_-13 to C-5/C-6/C-7 and H-4 to C-2/C-3/C-6. Relative configuration was assigned according to the ROE correlations. In particular, interactions between H-9β and H_3_-11 and between H-1α and H_3_-12 implied the same configuration as **1**. Additionally, correlations among H-9α/H_3_-11/H_3_-13 defined the 6*R* configuration. The absolute configuration of **2** was confirmed by comparison to heimionone A (**1**) as 1*S*,9*R*,6*R*.

The yellow oil heimionone C (**3**) was isolated from the mycelial extracts of liquid cultures with a molecular formula of C_22_H_30_O_4_, implying eight degrees of unsaturation. Analysis of the ^1^H and ^13^C spectra showed similarities to those of **1** and **2**. HMBC and COSY interactions confirmed the presence of the previously described dimethylcyclopentane substructure carrying a 4′-methylhexa-2′,4′-dienoc acid partial structure that was fused to C-1. A spin system was given based on ^1^H-^1^H COSY correlations among H_3_-14, H-6, H_2_-5, H_2_-4, H-3, and H_3_-15. Furthermore, HMBC correlations from H_3_-14 to C-6/C-7/C-8, H_2_-4 to C-3/C-5, and H_3_-15 to C-2/C-3/C-4 led to the identification of the 3,7-dimethylcycloocta-6,9-dien-8-one ring that was fused to the dimethylcyclopentane ring across C-2 and C-9 according to correlations from H-1/H-3/H_3_-15 to C-2 and from H-1/H-10/H_3_-12 to C-9. The relative configuration was deduced by an analysis of ROE data. Due to the key correlations among H-1α/H-4α/H-5α/H_3_-13/H_3_-15, these protons were arbitrarily assigned to the α face of the molecule. Correlations between H-3β/H-4β/H-5β and between H-10β/H_3_-12 indicated a β orientation of these protons. Finally, the absolute configuration was determined after heimionone C (**3**) was derivatized to its corresponding *S*- and *R*-MTPA esters at position C-10 in the course of Mosher’s method. The ∆δ*^SR^* chemical shift pattern (see Figure 3) indicated *R*-configuration of C-10 and thus a 1*S*,3*R*,10*R* absolute configuration.

Heimionone D (**4**) was obtained as a yellow oil from the mycelial extracts of liquid cultures with a molecular formula of C_22_H_30_O_3_. The 1D and 2D NMR data of **4** suggested a high similarity to **3**. They only differ in the absence of a hydroxy function, which was replaced by a methylene (δ_H_ 2.30 H-10α; 2.77, H-10β). This was confirmed by the HMBC correlations from H-1, H_3_-12, and H_3_-13 to C-10 (δ_C_ 47.8). The ROESY correlations among H-1α/H-4α/H-5α/H-10α/H_3_-13/H_3_-15 implied **4** has the same configuration as heimionone C (**3**).

Heimionone E (**5**) was isolated as a yellow oil from the mycelial extracts of liquid cultures. Its NMR spectroscopic data were highly similar to the ones of heimionone D (**4**). They only differ in the absence of the 4′-methylhexa-2′,4′-dienoc acid moiety that was replaced by the hydroxy function at position C-1. Finally, the absolute configuration was determined as 1*R*,3*SR* after heimionone E (**5**) was derivatized to its corresponding *S*- and *R*-MTPA esters at position C-1 using Mosher’s method with both *R*- and *S*-MTPA chloride.

Compounds **3**–**5** can be assigned to the asteriscanes according to their characteristic four methyl groups on the five-eight-membered ring system [9].

Although the bicyclo [5.3.0] decane scaffold of **1** and **2** is part of daucane-, isodaucane, aromadendrane, lactarane-, africane, gualane-, nor-guaiane and pseudoguaiane-type sesquiterpenoids, the methyl pattern of **1** and **2** with its germinal demethylation of C-10 is different from those [10]. Thus, the carbon backbone of **1** and **2** can be regarded as a new scaffold. Nevertheless, nardoguaianone E-I with similar structures were previously described after isolation from *Nardostachys chinensis* [11]. Interestingly, asteriscane-type sesquiterpenoids have been isolated from the soft coral *Sinularia capillosa* [12].

Furthermore, hispidin (**6**) [6] and hypholomin B (**7**) [7], both of which have been described to show antioxidant effects [13,14], were isolated from the submerged cultures of *Heimiomyces* sp., together with heimiomycins A (**11**) and B (**12**), which were previously published from fermentations of the same fungal strain under different conditions [4].

Interestingly, the secondary metabolite profile of *Heimiomyces* sp. mainly changed after the cultivation conditions were switched from solid to liquid media. While our previous experiments led to the isolation and identification of bis-heimiomycins A–D (**14**–**17**) and heimiomycins D–E (**18**–**19**) [5] from cultures on a solid rice medium, other compounds such as heimiocalamenes C–E (**8**–**10**), heimiomycins A–C (**11**–**13**) and heimiocalamenes A–B (**20**–**21**) [4,5], as well as the herein described heimionones A–E (**1**–**5**), were isolated after cultivation of *Heimiomyces* sp. in liquid YM6.3 and an MOF medium, respectively. Up to this point, this strain has already shown impressive chemical diversity. Since several other, yet unidentified, minor peaks were observed in the UV/Vis- and MS-spectra of its extracts, there is a potential even to find more exciting and new secondary metabolites if their production in more significant amounts can be triggered, for example by another change in the cultivation conditions.

### 2.2. Biological Assays

To evaluate the antimicrobial activity of compounds ***1***–***5*** a serial-dilution assay against several Gram-positive and Gram-negative bacteria as well as fungal strains was carried out, though no outstanding activities were observed (Table S4). Furthermore, all compounds were tested for their cytotoxicity against the human cervical cancer cell line KB3.1 and the mouse fibroblast cell line L929 where heimionone A (**1**) and heimionone C (**3**) showed weak cytotoxic effects (Table S5).

## 3. Materials and Methods

### 3.1. General Experimental Procedures

Optical rotations were measured using the 241 polarimeter (PerkinElmer, Waltham, MA, USA). Measurements of the UV spectra were carried out using the UV-Vis spectrophotometer UV-2450 (Shimadzu, Kyōto, Japan) and measurements of the ECD spectra were carried out using a J-815 spectropolarimeter (Jasco, Pfungstadt, Germany). NMR spectra were obtained using the Avance III 500 MHz spectrometer equipped with a BBFO (plus) SmartProbe (^1^H 500 MHz, ^13^C 125 MHz) and the Avance III 700 MHz spectrometer equipped with a 5 mm TCI cryoprobe (^1^H 700 MHz, ^13^C 175 MH) (both Bruker, Billerica, MA, USA). NMR data were referenced to selected chemical shifts of acetonitrile-*d*_3_ (^1^H: 1.94 ppm, ^13^C: 1.4 ppm), methanol-*d*_4_ (^1^H: 3.31 ppm, ^13^C: 49.2 ppm), and pyridine-*d*_5_ (^1^H: 7.22 ppm), respectively. HRESIMS mass spectra were recorded with a 1200 series HPLC-UV system (Agilent, Santa Clara, CA, USA) in combination with an ESI-TOF-MS (Maxis, Bruker). Performance of the measurements was conducted with a 2.1 × 50 mm, 1.7 µm, C18 Acquity UPLC BEH (Waters, Milford, MA, USA) column, using MilliQ H_2_O + 0.1% formic acid as solvent A and MeCN + 0.1% formic acid as solvent B (gradient: 5% B for 0.5 min increasing to 100% B in 19.5 min and maintaining 100% B for 5 min, flow rate: 0.6 mL/min, UV detection: 200–600 nm). 

### 3.2. Fungal Material

*Heimiomyces* sp. (MUCL 56078) was collected by C. Decock and J. C. Matasyoh from Mount Elgon National Reserve in Kenya (1°7′6″ N, 34°31′30″ E). The genus was identified, and a dried specimen was deposited, as previously described by Cheng et al. [4].

### 3.3. Seed Culture and Fermentation of Heimiomyces sp.

The maintenance of *Heimiomyces* sp. cultures was carried out on YM6.3 agar plates. A 500 mL Erlenmeyer shape culture flask containing 200 mL of YM6.3 medium (10 g/L malt extract, 4 g/L d-glucose, 4 g/L yeast extract, pH 6.3) was used for inoculation with three 50 mm^2^ sized pieces of well-grown mycelium from YM6.3 agar plates. The seed culture was incubated at 23 °C and 140 min^−1^ on a rotary shaker for 23 days. For the following homogenization an Ultra-Turrax^®^ (T25 easy clean digital, IKA, Staufen im Breisgau, Germany), equipped with an S 25 N–25 F dispersing tool was used at 8000 rpm for 10–20 s. The homogenized culture broth was utilized as inoculum by transferring 3 mL per flask into 15 500 mL Erlenmeyer shape culture flasks containing 200 mL of YM6.3 medium and subsequently, the incubation was performed at 23 °C and 140 min^−1^ on a rotary shaker. Consumption of the glucose was monitored using test strips (Medi-Test Glucose, Macherey-Nagel, Düren, Germany) leading to a termination of the fermentation process two days after the culture broth tested negative for glucose.

### 3.4. Harvest and Extraction

Separation of the mycelium and supernatant was carried out by centrifugation at 5100 min^−1^ for 15 min (lab centrifuge 4-16KS, Sigma Laborzentrifugen GmbH, Osterode am Harz, Germany). Extraction of the mycelium was performed with acetone in an ultrasonic bath for 30 min, twice. The liquid phase was evaporated at 40 °C after separation from the solid phase by filtration, leading to a remaining aqueous phase that was subsequently diluted with water and extracted against ethyl acetate. Afterward, the organic phase was evaporated to dryness (40 °C) and led to 607 mg of extract from the mycelium. Extraction of the supernatant was carried out with ethyl acetate (1:1) in a separatory funnel, twice. Evaporation of the organic phase at 40 °C led to 251 mg extract from the supernatant of YM6.3 cultures. Both extracts were filtered using the SPME Strata^TM^-X 33 µm Polymeric RP cartridge (Phenomenex, Aschaffenburg, Germany).

### 3.5. Analytical HPLC

The extracts obtained from liquid cultures of *Heimiomyces* sp. were dissolved in acetone to yield a concentration of 10 mg/mL. Analysis of the samples was performed with an analytical HPLC device (Dionex UltiMate 3000 series, Sunnyvale, CA, USA) coupled to an ion trap mass spectrometer (amazon speed™ by Bruker). As mobile phase HPLC grade water and MeCN, both containing 0.1% of formic acid, were used. After injection of 2 μL of the samples, the separation was carried out over an ACQUITY-UPLC^®^ BEH C18 column (50 × 2.1 mm; particle size: 1.7 μm) (Waters) with a flow rate of 600 μL/min. The gradient started at 5% of MeCN, then increased to 100% MeCN in 20 min and remained for 5 min at 100%. To evaluate the obtained chromatograms, the appropriate analysis software (Data Analysis, version 4.4 by Bruker) was used.

### 3.6. Isolation of Compounds ***1***–***5*** via Reversed-Phase Liquid Chromatography

After evaluation of the analytical data, the extracts were separated via RP HPLC using a Gilson PLC 2250 Purification System (Limburg, Germany). The extract obtained from the supernatant of the culture broth was purified using the Synergi^TM^ Polar RP 250 × 50 mm, 80 Å, 10 µm (Phenomenex); solvent A: MilliQ water + 0.1% formic acid, solvent B: acetonitrile + 0.1% formic acid, flow rate: 50 mL/min, gradient: 5 min B at 20%, increasing to 100% B in 80 min, maintaining 100% B for 10 min. This yielded compound **5** (3.83 mg, t_R_ = 44.0–44.75 min), compound **2** (2.46 mg, t_R_ = 47.5–48.5 min), compound **1** (3.5 mg, t_R_ = 49.5–50.5 min) and compound **3** (1.26 mg, t_R_ = 65.0–66.0 min). The extract obtained from the mycelium of the culture broth was purified using the same equipment and conditions. This yielded compound **1** (1.77 mg, t_R_ = 49.5–50.5 min), compound **3** (1.87 mg, t_R_ = 58.5–59.5 min), and compound **4** (2.31 mg, t_R_ = 68.0–68.5 min). 

### 3.7. Spectral Data of Compounds ***1***–***5***

Heimionone A (**1**): yellow oil; [α]^25^_D_ +250 (*c* 0.50, MeOH); UV/vis (0.01 mg/mL, MeOH) λ_max_(logε): 325 (3.99), 268 (4.48), 247 (4.48), 202 (3.82) nm; ECD (0.2 mg/mL, MeOH) λ(Δε): 265 (+19.8), 243 (−18.0), 220 (−1.2), 202 (−7.6) nm, Figure S1; ^1^H and ^13^C NMR data (MeOH-*d*_4_), Table 1 and Table S6; ESIMS *m*/*z* 373.19 [M + H]^+^, 371.24 [M − H]^−^; HRESIMS *m*/*z* 373.2012 [M + H]^+^ (calcd. for C_22_H_29_O_5_, 373.2023); t_R_ = 9.2 min (analytical HPLC).

Heimionone B (**2**): yellow oil; [α]^25^_D_ +230 (*c* 0.10, MeOH); UV/vis (0.01 mg/mL, MeOH) λ_max_(logε): 329 (3.70), 267 (4.23), 248 (4.22), 198 (4.73) nm; ECD (0.2 mg/mL, MeOH) λ(Δε): 266 (+10.2), 241 (−7.7), 222 (+0.1) nm, Figure S1; ^1^H and ^13^C NMR data (MeOH-*d*_4_), Table 1 and Table S7); ESIMS *m*/*z* 373.21 [M + H]^+^, 371.04 [M − H]^−^; HRESIMS *m*/*z* 373.2011 [M + H]^+^ (calcd. for C_22_H_29_O_5_, 373.2010); t_R_ = 9.3 min (analytical HPLC).

Heimionone C (**3**): yellow oil; [α]^25^_D_ +156 (*c* 0.50, MeOH); UV/vis (0.01 mg/mL, MeOH) λ_max_(logε): 376 (3.18), 269 (4.35), 198 (4.41) nm; ECD (0.2 mg/mL, MeOH) λ(Δε): 270 (+8.9), 241 (−0.6), 219 (+0.2), 205 (−10.8) nm, Figure S2; ^1^H and ^13^C NMR data (acetonitrile-*d*_3_), Table 1 and Table S8; ESIMS *m*/*z* 341.25 [M − H_2_O + H]^+^, 375.28 [M – H + H_2_O]^−^; HRESIMS *m*/*z* 381.2034 [M + Na]^+^ (calcd. for C_22_H_30_NaO_4_, 381.2036); t_R_ = 11.9 min (analytical HPLC).

Heimionone D (**4**): yellow oil; [α]^25^_D_ +72 (*c* 1.00, MeOH); UV/vis (0.01 mg/mL, MeOH) λ_max_(logε): 268 (3.68), 198 (4.50) nm; ECD (0.4 mg/mL, MeOH) λ(Δε): 270 (+1.9), 247 (−0.7), 218 (+0.6), 200 (−3.7) nm, Figure S2; ^1^H and ^13^C NMR data (acetonitrile-*d*_3_) Table 1 and Table S9; ESIMS *m*/*z* 365.20 [M + Na]^+^; HRESIMS *m*/*z* 365.2086 [M + Na]^+^ (calcd. for C_22_H_30_NaO_3_, 365.2087); t_R_ = 14.1 min (analytical HPLC).

Heimionone E (**5**): yellow oil; [α]^25^_D_ +20 (*c* 0.25, MeOH); UV/vis (0.05 mg/mL, MeOH) λ_max_(logε): 266 (3.83), 202 (3.82) nm; ECD (1.0 mg/mL, MeOH) λ(Δε): 404 (+0.2), 361 (−1.0), 319 (+0.2), 255 (−2.0), 225 (+1.0), 204 (−6.8) nm, Figure S2; ^1^H and ^13^C NMR data (methanol-*d*_4_, Table 1 and Table S10; ESIMS *m*/*z* 235.07 [M + H]^+^, 251.06 [M – H + H_2_O]^−^; HRESIMS *m*/*z* 235.1694 [M + H]^+^ (calcd. for C_15_H_23_O_2_, 235.1693); t_R_ = 8.1 min (analytical HPLC).

### 3.8. Preparation of the (R)- and (S)-MTPA Ester Derivatives of ***1***, ***3***, and ***5***

The 0.5 mg of each compound were dissolved in 300 µL deuterated pyridine. Afterward, 2 µL of (*R*)-(−)-α-methoxy-α-(trifluoromethyl)phenylacetyl chloride were added into the solution and left for 15 min at room temperature. The reaction was monitored by analytical HPLC/MS. Another 2 µL of *R*-MTPA were added if the compounds were not completely converted into the corresponding Mosher-ester. Immediately after Mosher esterification, the samples were transferred into 3.0 mm NMR tubes. This was followed by measurements of ^1^H NMR (Tables S1–S3), ^1^H,^1^H-COSY NMR, ^1^H,^13^C-HSQC NMR, and ^1^H,^13^C-HMBC NMR spectra. The same procedure was repeated with another 0.5 mg of each compound using (*S*)-(+)- α-methoxy- α-(trifluoromethyl)phenylacetyl chloride. Evaluation of the obtained Δδ*^SR^* values was conducted as described by Hoye et al. [15].

### 3.9. Antimicrobial Assay

Antimicrobial activities of all isolated compounds were assessed by performing a serial-dilution assay resulting in the determination of their minimum inhibitory concentration (MIC) against several yeast, fungal, and bacterial strains (Table S4). The assay was carried out in 96-well microtiter plates, as previously described by Harms et al. [16].

### 3.10. Cytotoxicity Assay

The in vitro cytotoxicity of compounds **1**–**5** against the mouse fibroblast cell line L929 and the cervix carcinoma cell line KB3.1 (Table S5) was assessed in 96-well plates, as previously published by Harms et al. [15].

## 4. Conclusions

Five new terpenoids, namely heimionones A-E (**1**–**5**), with uncommon bicyclo [5.3.0] decane and [6.3.0] undecane core structures, respectively, were isolated from submerged cultures of *Heimiomyces* sp. (MUCL 56078). In association with the previously described hispidin (**6**), hypholomin B (**7**), heimiocalamenes C–E (**8**–**10**), and heimiomycins A–C (**11**–**13**), as well as bis-heimiomycins A–D (**14**–**17**), heimiomycins D–E (**18**–**19**), and heimiocalamenes A–B (**20**–**21**), it becomes clear that *Heimiomyces* sp. is capable of producing a chemically very diverse spectrum of secondary metabolites. On top of that, a high number of as yet unidentified minor peaks were observed in the UV/Vis- and MS-spectra of the extracts obtained from *Heimiomyces* sp., therefore especially this strain should be considered for further analysis of its secondary metabolism, particularly for cultivation in more different media. Since all isolated compounds did not show outstanding activities in the antimicrobial and cytotoxicity assays, they can be considered for other assays with different targets. However, our findings once again show the importance of examining unexplored species from the tropics for their secondary metabolism during the search for novel natural products.

## Figures and Tables

**Table 1:** ^1^H and ^13^C spectroscopic data of compounds **1**, **2**, and **5** in methanol-*d*_4_ and **3**–**4** in acetonitrile-*d*_3_ (δ in ppm).

	1 ^b,d^		2 ^b,d^		3 ^a,c^		4 ^b,d^		5 ^b,d^	
No.	δ_C_, Type	δ_H_ (*J* in Hz)	δ_C_, Type	δ_H_ (*J* in Hz)	δ_C_, Type	δ_H_ (*J* in Hz)	δ_C_, Type	δ_H_ (*J* in Hz)	δ_C_, Type	δ_H_ (*J* in Hz)
1	83.5, CH	α: 6.12, s	86.1, CH	α: 6.32, m	83.1, CH	α: 5.62, s	85.6, CH	α: 5.58, s	85.3, CH	α: 4.13, s
2	153.0, C		146.2, C		150.3, C		150.3, C		156.5, C	
3	152.4, C		133.5, C		31.2, CH	ß: 2.86, dqd (12.8, 6.9, 6.0)	31.2, CH	ß: 2.96, m	31.9, CH	β: 2.98, dqd (12.5, 6.9, 5.0)
4	131.8, CH	7.56, d (9.8)	134.8, C	6.26, d (11.8)	37.1, CH_2_	α: 1.57, m β: 1.78, m	37.3, CH_2_	α: 1.53, m β: 1.74, m	38.3, CH_2_	α: 1.82, m β: 1.51, m
5	138.6, CH	7.69, dd (9.8, 0.9)	130.5, CH	5.53, d (11.8)	26.3, CH_2_	α: 2.46, m β: 2.11, m	26.3, CH_2_	α: 2.07, m β: 2.32, m	26.7, CH_2_	α: 2.30, m β: 2.08, m
6	151.7, C		78.7, C		140.9, CH	6.47, m	139.1, CH	6.35, ddd (9.7, 8.4, 1.4)	139.4, CH	6.36, ddd (9.8, 8.4, 1.4)
7	187.5, C		199.3, C		142.0, C		141.5, C		142.1, C	
8	146.1, C		142.2, C		194.2, C		194.3, C		196.3, C	
9	84.2, CH	ß: 5.14, s	83.4, CH	ß: 4.61, s	148.9, C		146.9, C		144.7, C	
10	45.5, C		47.2, C		83.3, CH	ß: 4.89, s	47.8, CH_2_	α: 2.30, d (16.4) β: 2.77, d (16.4)	47.2, CH_2_	α: 2.21, d (16.1) β: 2.76, d (16.1)
11	20.5, CH_3_	1.13, s	22.0, CH_3_	0.98, m	44.2, C		39.4, C		39.6, C	
12	20.7, CH_3_	1.02, s	21.7, CH_3_	1.18, m	20.4, CH_3_	1.01, s	22.2, CH_3_	0.99, s	22.5, CH_3_	1.13, s
13	22.3, CH_3_	2.31, br s	27.2, CH_3_	1.45, m	21.4, CH_3_	0.98, s	28.0, CH_3_	1.06, s	28.3, CH_3_	0.96, s
14	67.8, CH	ß: 4.70, q (6.3)	141.6, CH	6.16, q (7.7)	20.1, CH_3_	1.89, s	20.3, CH_3_	1.88, s	20.3, CH_3_	1.90, s
15	26.1, CH_3_	1.40, d (6.3)	16.9, CH_3_	1.90, d (7.7)	16.7, CH_3_	0.95, d (6.9)	17.0, CH_3_	0.97, d (6.9)	18.0, CH_3_	1.26, d (6.9)
1′	168.6, C		168.7, C		167.7, C		167.6, C			
2′	115.1, CH	5.81, d (15.7)	115.3, CH	5.81, d (15.7)	115.7, CH	5.79, d (15.7)	115.9, CH	5.81, d (15.7)		
3′	152.6, CH	7.35, d (15.7)	152.2, CH	7.33, d (15.7)	150.9, CH	7.30, d (15.7)	150.8, CH	7.31, d (15.7)		
4′	135.3, C		135.3, C		134.9, C		134.9, C			
5′	139.2, CH	6.07, q (7.0)	139.0, CH	6.06, q (7.1)	138.4, CH	6.06, q (7.0)	138.2, CH	6.06, q (6.9)		
6′	14.8, CH_3_	1.82, d (7.0)	14.8, CH_3_	1.84, d (7.1)	14.8, CH_3_	1.80, d (7.0)	14.8, CH_3_	1.80, d (6.9)		
7′	11.9, CH_3_	1.77, m	11.9, CH_3_	1.79, m	12.0, CH_3_	1.77, s	12.0, CH_3_	1.77, br s		

^a 1^H 500 MHz, ^b 1^H 700 MHz; ^c 13^C 125 MHz, ^d 13^C 175 MHz.

## Data Availability

The data are available in the Supporting Information of the article.

## Supplementary Materials

The following supporting information can be downloaded at: https://www.mdpi.com/article/10.3390/molecules28093723/s1, Figure S1. ECD spectra of **1** and **2**; Figure S2. ECD spectra of **3**–**5**; Figure S3. ^1^H NMR spectrum (700 MHz, pyridine-*d*_5_) of the *S*-MTPA ester of heimionone A (**1**); Figure S4. COSY NMR spectrum (700 MHz, pyridine-*d*_5_) of the *S*-MTPA ester of heimionone A (**1**); Figure S5. ^1^H NMR spectrum (700 MHz, pyridine-*d*_5_) of the *R*-MTPA ester of heimionone A (**1**); Figure S6. COSY NMR spectrum (700 MHz, pyridine-*d*_5_) of the *R*-MTPA ester of heimionone A (**1**); Figure S7. ^1^H NMR spectrum (700 MHz, pyridine-*d*_5_) of the *S*-MTPA ester of heimionone C (**3**); Figure S8. COSY NMR spectrum (700 MHz, pyridine-*d*_5_) of the *S*-MTPA ester of heimionone C (**3**); Figure S9. ^1^H NMR spectrum (700 MHz, pyridine-*d*_5_) of the *R*-MTPA ester of heimionone C (**3**); Figure S10. COSY NMR spectrum (700 MHz, pyridine-*d*_5_) of the *R*-MTPA ester of heimionone C (**3**); Figure S11. ^1^H NMR spectrum (700 MHz, pyridine-*d*_5_) of the *S*-MTPA ester of heimionone E (**5**); Figure S12. ^1^H NMR spectrum (700 MHz, pyridine-*d*_5_) of the *R*-MTPA ester of heimionone E (**5**); Figure S13. COSY NMR spectrum (700 MHz, pyridine-*d*_5_) of the *R*-MTPA ester of heimionone E (**5**); Figure S14. Previously described compounds that were observed in submerged cultures of *Heimiomyces* sp. **6**: hispidin, **7**: hypholomin B.; Figure S15. Compounds previously isolated from *Heimiomyces* sp.: **8**–**10**: heimiocalamenes C–E (including originally proposed and revised structure of **10**), **11**–**13**: heimiomycins A–C; Figure S16. Compounds previously isolated from *Heimiomyces* sp. **14**–**17**: bis-heimiomycins A–D, **18**–**19**: heimiomycins D–E, **20**–**21**: heimiocalamenes A–B; Figure S17. Chemical structures of heimionone A (**1**) and daldinin F [8]; Figures S18–S47: NMR spectra. Table S1. ^1^H NMR data (700 MHz, Pyridine-*d*_5_, δ in ppm) of (*S*)/(*R*) MTPA esters obtained from heimionone A (**1**); Table S2. ^1^H NMR data (700 MHz, Pyridine-*d*_5_, δ in ppm) of (*S*)/(*R*) MTPA esters obtained from heimionone C (**3**); Table S3. ^1^H NMR data (700 MHz, Pyridine-*d*_5_, δ in ppm) of (*S*)/(*R*) MTPA esters obtained from heimionone E (**5**); Table S4. Minimum inhibitory concentration (MIC in µg/mL) for bacterial and fungal strains; Table S5. Half inhibitory concentration (IC_50_ in µM); Table S6. NMR spectroscopic data (^13^C (δC), 175 MHz and ^1^H (δH), 500 MHz, methanol-*d*_4_) for heimionone A (**1**); Table S7. NMR spectroscopic data (^13^C (δC), 175 MHz and ^1^H (δH), 700 MHz, methanol-*d*_4_) for heimionone B (**2**); Table S8. NMR spectroscopic data (^13^C (δC), 125 MHz and ^1^H (δH), 500 MHz, acetonitrile-*d*_3_) for heimionone C (**3**); Table S9. NMR spectroscopic data (^13^C (δC), 175 MHz and ^1^H (δH), 700 MHz, acetonitrile-*d*_3_) for heimionone D (**4**); Table S10. NMR spectroscopic data (^13^C (δC), 175 MHz and ^1^H (δH), 700 MHz, methanol-*d*_4_) for heimionone E (**5**); Table S11. Comparison of the NMR spectroscopic data for the 4-methylhexa-2,4-dienoic acid partial structure of heimionone A (**1**) (^13^C (δC), 175 MHz and ^1^H (δH), 500 MHz, methanol-*d*_4_) and daldinin F (^13^C (δC), 150 MHz and ^1^H (δH), 600 MHz, chloroform-*d*_1_) [8].

## Sample Availability

Samples of the isolated compounds are not available from the authors, due to consumption for structure elucidation and bioassays.
