# Supplementary material for: Heimionones A–E, New Sesquiterpenoids Produced by Heimiomyces sp., a Basidiomycete Collected in Africa
Source: Molecules. 2023 Apr 26;28(9):3723. doi: 10.3390/molecules28093723 (PMC10179880; doi:10.3390/molecules28093723)
Supplement: Supplementary file 1 [file molecules-28-03723-s001.zip › molecules-2356625-supplementary.pdf]

Heimionones A–E, New Sesquiterpenoids Produced by *Heimiomyces* sp., a Basidiomycete Collected in Africa – Supporting information

Sebastian Pfütze [1,2], Atchara Khamsim [1,2], Frank Surup [1,2], Cony Decock [3], Josphat C. Matasyoh [4] and Marc Stadler [1,2,*]

[1] Department of Microbial Drugs, Helmholtz Centre for Infection Research (HZI), German Centre for Infection Research (DZIF), Partner Site Hannover/Braunschweig, Inhoffenstrasse 7, 38124 Braunschweig, Germany
[2] Institute of Microbiology, Technische Universität Braunschweig, Spielmannstraße 7, 38106 Braunschweig, Germany
[3] Earth and Life Institute, Mycothéque de l' Universite Catholique de Louvain (BCCM/MUCL), Place Croix du Sud 3, B-1348 Louvain-la-Neuve, Belgium
[4] Department of Chemistry, Egerton University, P.O. Box 536, Njoro 20115, Kenya
* Correspondence: marc.stadler@helmholtz-hzi.de; Tel.: +49-531-6181-4240

Content

1. Experimental Section 4
  1.1. ECD spectra of compounds 1-5. 4
  1.2. NMR spectroscopic data of the (*S*)- and (*R*)-MTPA Ester Derivatives of 1, 3 and 5. 6
  1.3. Evaluation of antimicrobial activity. 20
  1.4. Evaluation of cytotoxicity. 20
2. Structures of known compounds 21
3. NMR spectroscopic data of 1-5. 23
4. Literature 30

List of Figures

Figure S1. ECD spectra of 1 and 2. 4
Figure S2. ECD spectra of 3-5.......... 5
Figure S3. ^{1}H NMR spectrum (700 MHz, pyridine-d_5) of the *S*-MTPA ester of heimionone A (1)....... 7
Figure S4. COSY NMR spectrum (700 MHz, pyridine-d_5) of the *S*-MTPA ester of heimionone A (1)... 8
Figure S5. ^{1}H NMR spectrum (700 MHz, pyridine-d_5) of the *R*-MTPA ester of heimionone A (1)....... 9
Figure S6. COSY NMR spectrum (700 MHz, pyridine-d_5) of the *R*-MTPA ester of heimionone A (1). 10
Figure S7. ^{1}H NMR spectrum (700 MHz, pyridine-d_5) of the *S*-MTPA ester of heimionone C (3)...... 12
Figure S8. COSY NMR spectrum (700 MHz, pyridine-d_5) of the *S*-MTPA ester of heimionone C (3). 13
Figure S9. ^{1}H NMR spectrum (700 MHz, pyridine-d$_5$) of the *R*-MTPA ester of heimionone C (3)...... 14
Figure S10. COSY NMR spectrum (700 MHz, pyridine-d_5) of the *R*-MTPA ester of heimionone C (3). 15
Figure S11. ^{1}H NMR spectrum (700 MHz, pyridine-d_5) of the *S*-MTPA ester of heimionone E (5). ... 17
Figure S12. ^{1}H NMR spectrum (700 MHz, pyridine-d_5) of the *R*-MTPA ester of heimionone E (5). ... 18
Figure S13. COSY NMR spectrum (700 MHz, pyridine-d_5) of the *R*-MTPA ester of heimionone E (5). 19
Figure S14. Previously described compounds that were observed in submerged cultures of *Heimiomyces* sp. 6: hispidin, 7: hypholomin B. 21
Figure S15. Compounds previously isolated from *Heimiomyces* sp.: 8-10: heimiocalamenes C-E (including originally proposed and revised structure of 10), 11-13: heimiomycins A-C. 21
Figure S16. Compounds previously isolated from *Heimiomyces* sp. 14-17: bis-heimiomycins A-D, 18-19: heimiomycins D-E, 20-21: heimiocalamenes A-B. 22
Figure S17. Chemical structures of heimionone A (1) and daldinin F [1]. 28
Figure S18. ^{1}H NMR spectrum (500 MHz, methanol-d_4) of heimionone A (1). 31
Figure S19. ^{13}C NMR spectrum (175 MHz, methanol-d_4) of heimionone A (1). 32
Figure S20. COSY NMR spectrum (500 MHz, methanol-d_4) of heimionone A (1). 33
Figure S21. HSQC NMR spectrum (500 MHz, methanol-d_4) of heimionone A (1). 34
Figure S22. HMBC NMR spectrum (500 MHz, methanol-d_4) of heimionone A (1). 35
Figure S23. ROESY NMR spectrum (500 MHz, methanol-d_4) of heimionone A (1). 36
Figure S24. ^{1}H NMR spectrum (700 MHz, methanol-d_4) of heimionone B (2). 37
Figure S25. ^{13}C NMR spectrum (175 MHz, methanol-d_4) of heimionone B (2). 38
Figure S26. COSY NMR spectrum (700 MHz, methanol-d_4) of heimionone B (2). 39
Figure S27. HSQC NMR spectrum (700 MHz, methanol-d_4) of heimionone B (2). 40
Figure S28. HMBC NMR spectrum (700 MHz, methanol-d_4) of heimionone B (2). 41
Figure S29. ROESY NMR spectrum (700 MHz, methanol-d_4) of heimionone B (2). 42
Figure S30. ^{1}H NMR spectrum (500 MHz, acetonitrile-d_3) of heimionone C (3). 43
Figure S31. ^{13}C NMR spectrum (125 MHz, acetonitrile-d_3) of heimionone C (3). 44
Figure S32. COSY NMR spectrum (500 MHz, acetonitrile-d_3) of heimionone C (3). 45
Figure S33. HSQC NMR spectrum (500 MHz, acetonitrile-d_3) of heimionone C (3). 46
Figure S34. HMBC NMR spectrum (500 MHz, acetonitrile-d_3) of heimionone C (3). 47
Figure S35. ROESY NMR spectrum (500 MHz, acetonitrile-d_3) of heimionone C (3). 48
Figure S36. ^{1}H NMR spectrum (700 MHz, acetonitrile-d_3) of heimionone D (4). 49
Figure S37. ^{13}C NMR spectrum (175 MHz, acetonitrile-d_3) of heimionone D (4). 50
Figure S38. COSY NMR spectrum (700 MHz, acetonitrile-d_3) of heimionone D (4). 51
Figure S39. HSQC NMR spectrum (700 MHz, acetonitrile-d_3) of heimionone D (4). 52
Figure S40. HMBC NMR spectrum (700 MHz, acetonitrile-d_3) of heimionone D (4). 53
Figure S41. ROESY NMR spectrum (700 MHz, acetonitrile-d_3) of heimionone D (4). 54
Figure S42. ^{1}H NMR spectrum (700 MHz, methanol-d_4) of heimionone E (5). 55

Figure S43. ^{13}C **NMR spectrum (175 MHz, methanol-*d*$_4$) of heimionone E (5).** 56
Figure S44. COSY NMR spectrum (700 MHz, methanol-*d*$_4$) of heimionone E (5). 57
Figure S45. HSQC NMR spectrum (700 MHz, methanol-*d*$_4$) of heimionone E (5). 58
Figure S46. HMBC NMR spectrum (700 MHz, methanol-*d*$_4$) of heimionone E (5). 59
Figure S47. ROESY NMR spectrum (700 MHz, methanol-*d*$_4$) of heimionone E (5). 60

List of tables

Table S1. ^{1}H **NMR data (700 MHz, Pyridine-*d*$_5$, δ in ppm) of (*S*)/(*R*) MTPA esters obtained from heimionone A (1)** 6
Table S2. ^{1}H **NMR data (700 MHz, Pyridine-*d*$_5$, δ in ppm) of (*S*)/(*R*) MTPA esters obtained from heimionone C (3)** 11
Table S3. ^{1}H **NMR data (700 MHz, Pyridine-*d*$_5$, δ in ppm) of (*S*)/(*R*) MTPA esters obtained from heimionone E (5).** 16
Table S4. Minimum inhibitory concentration (MIC in µg/mL) for yeast, bacterial and fungal strains. 20
Table S5. Half inhibitory concentration (IC_{50} in µM) 20
Table S6. NMR spectroscopic data (^{13}C (δ_C), 175 MHz and ^{1}H (δ_H), 500 MHz, methanol-*d*$_4$) for heimionone A (1). 23
Table S7. NMR spectroscopic data (^{13}C (δ_C), 175 MHz and ^{1}H (δ_H), 700 MHz, methanol-*d*$_4$) for heimionone B (2). 24
Table S8. NMR spectroscopic data (^{13}C (δ_C), 125 MHz and ^{1}H (δ_H), 500 MHz, acetonitrile-*d*$_3$) for heimionone C (3) 25
Table S9. NMR spectroscopic data (^{13}C (δ_C), 175 MHz and ^{1}H (δ_H), 700 MHz, acetonitrile-*d*$_3$) for heimionone D (4) 26
Table S10. NMR spectroscopic data (^{13}C (δ_C), 175 MHz and ^{1}H (δ_H), 700 MHz, methanol-*d*$_4$) for heimionone E (5). 27
Table S11. Comparison of the NMR spectroscopic data for the 4-methylhexa-2,4-dienoic acid partial structure of heimionone A (1) (^{13}C (δ_C), 175 MHz and ^{1}H (δ_H), 500 MHz, methanol-*d*$_4$) and daldinin F (^{13}C (δ_C), 150 MHz and ^{1}H (δ_H), 600 MHz, chloroform-*d*$_1$) [1]. 29

1. Experimental Section

1.1. ECD spectra of compounds 1-5.

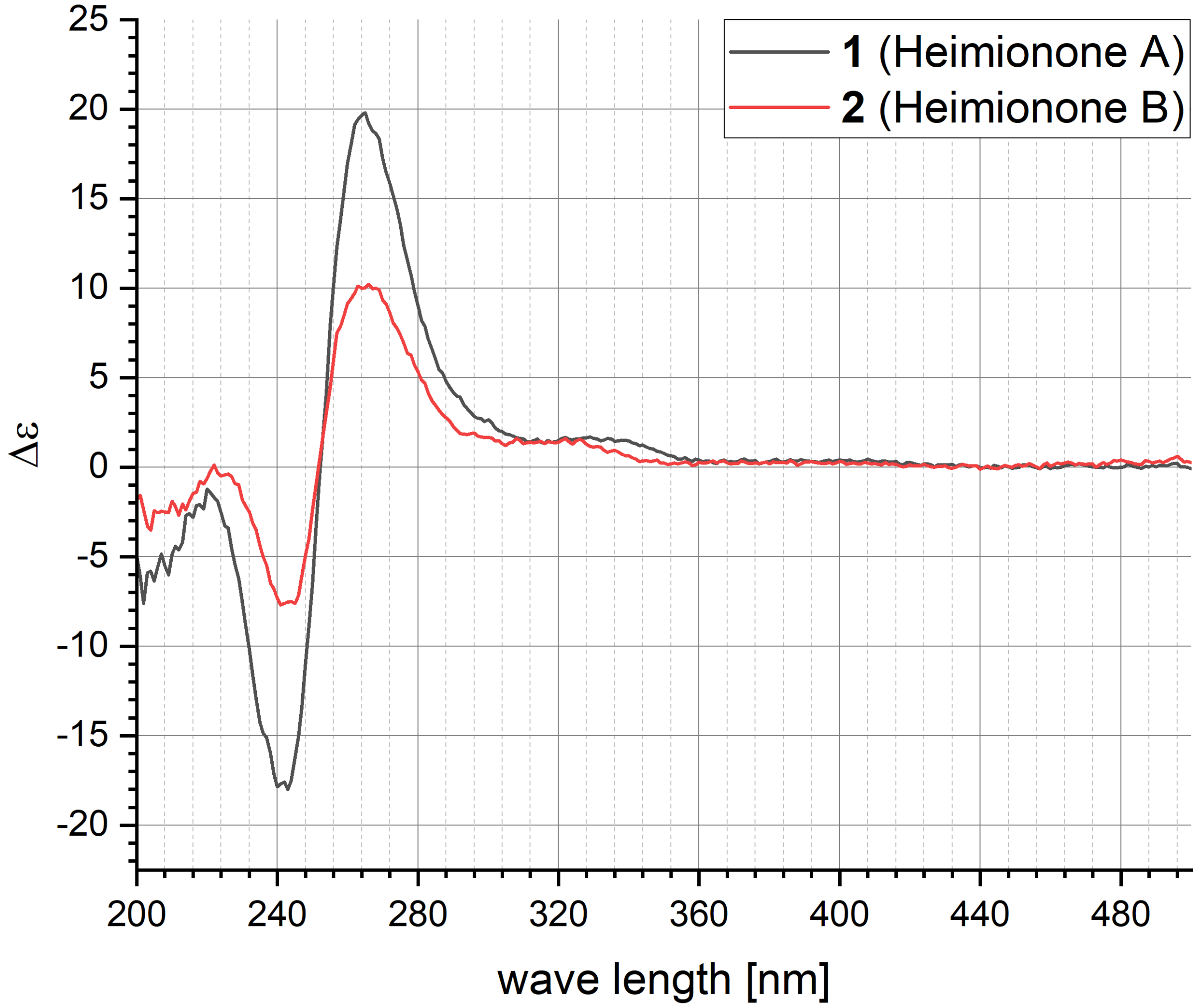

Figure S1. ECD spectra of **1** and **2.**

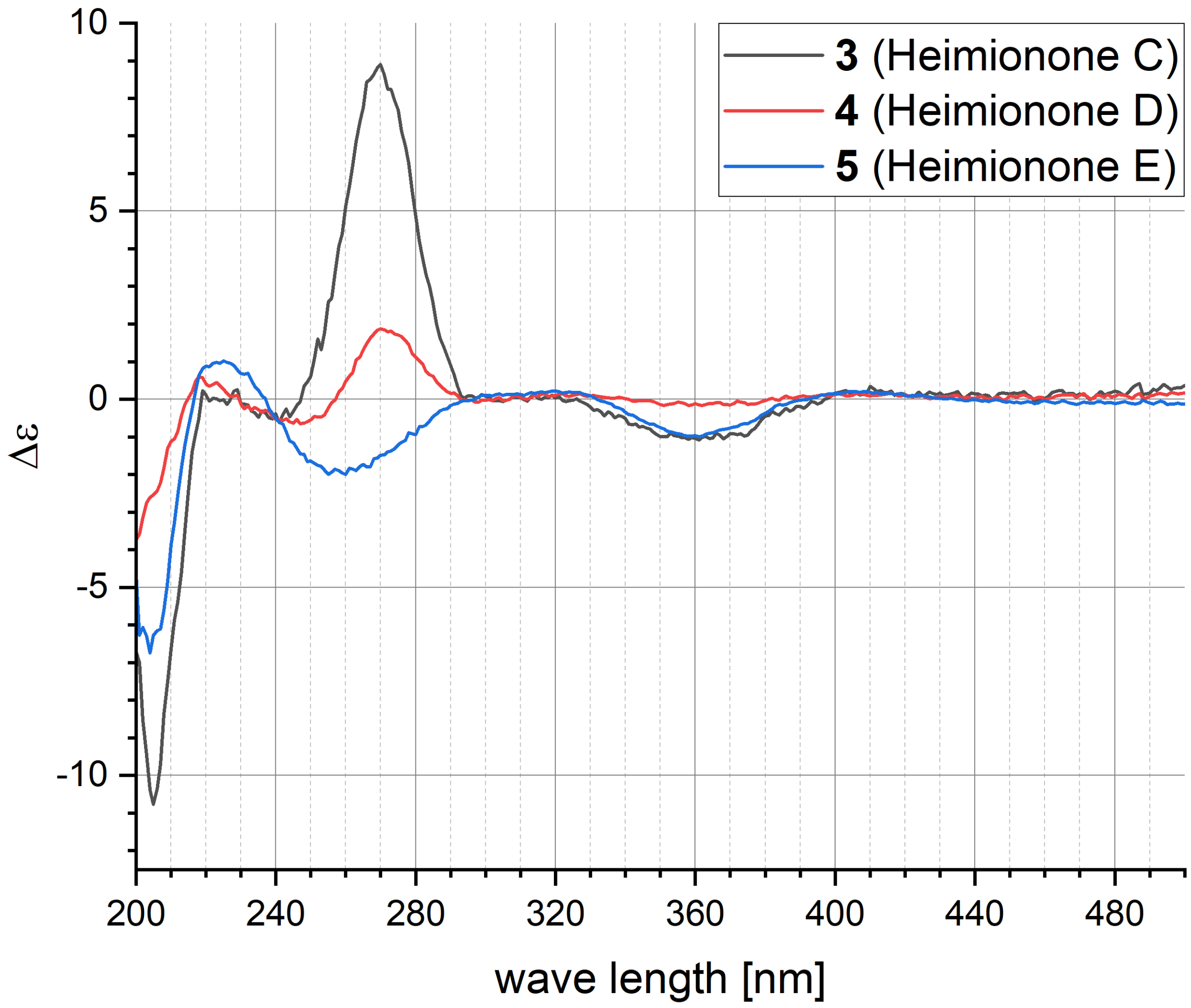

Figure S2. ECD spectra of **3**-**5**.

1.2. NMR spectroscopic data of the (*S*)- and (*R*)-MTPA Ester Derivatives of 1, 3 and 5.

Table S1. ^{1}H NMR data (700 MHz, Pyridine-*d*$_5$, δ in ppm) of (*S*)/(*R*) MTPA esters obtained from heimionone A (**1**).

atom	heimionone A (1)	(*S*)-MTPA ester	(*R*)-MTPA ester	Δδ*SR*
**1**	6.59	6.63	6.50	0.13
**4**	7.99	7.32	6.94	0.38
**5**	7.52	7.31	7.17	0.14
**9**	5.55	7.15	7.16	-0.01
**11**	1.38	1.37	1.33	0.04
**12**	1.33	1.31	0.94	0.37
**13**	2.32	2.23	2.28	-0.05
**14**	5.34	6.33	6.26	0.07
**15**	1.70	1.61	1.63	-0.02

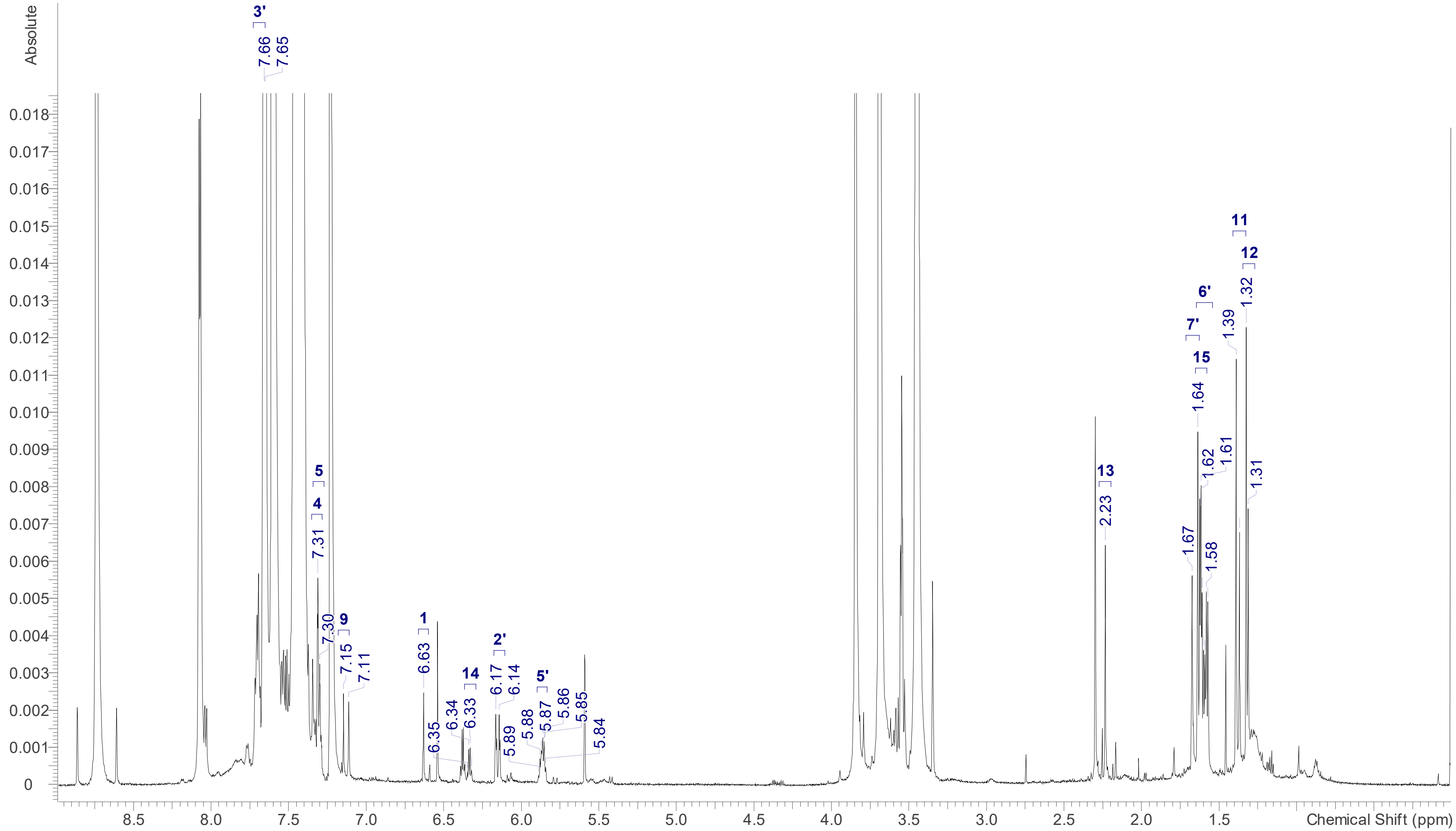

Figure S3. ^{1}H NMR spectrum (700 MHz, pyridine-d_5) of the *S*-MTPA ester of heimionone A (**1**).

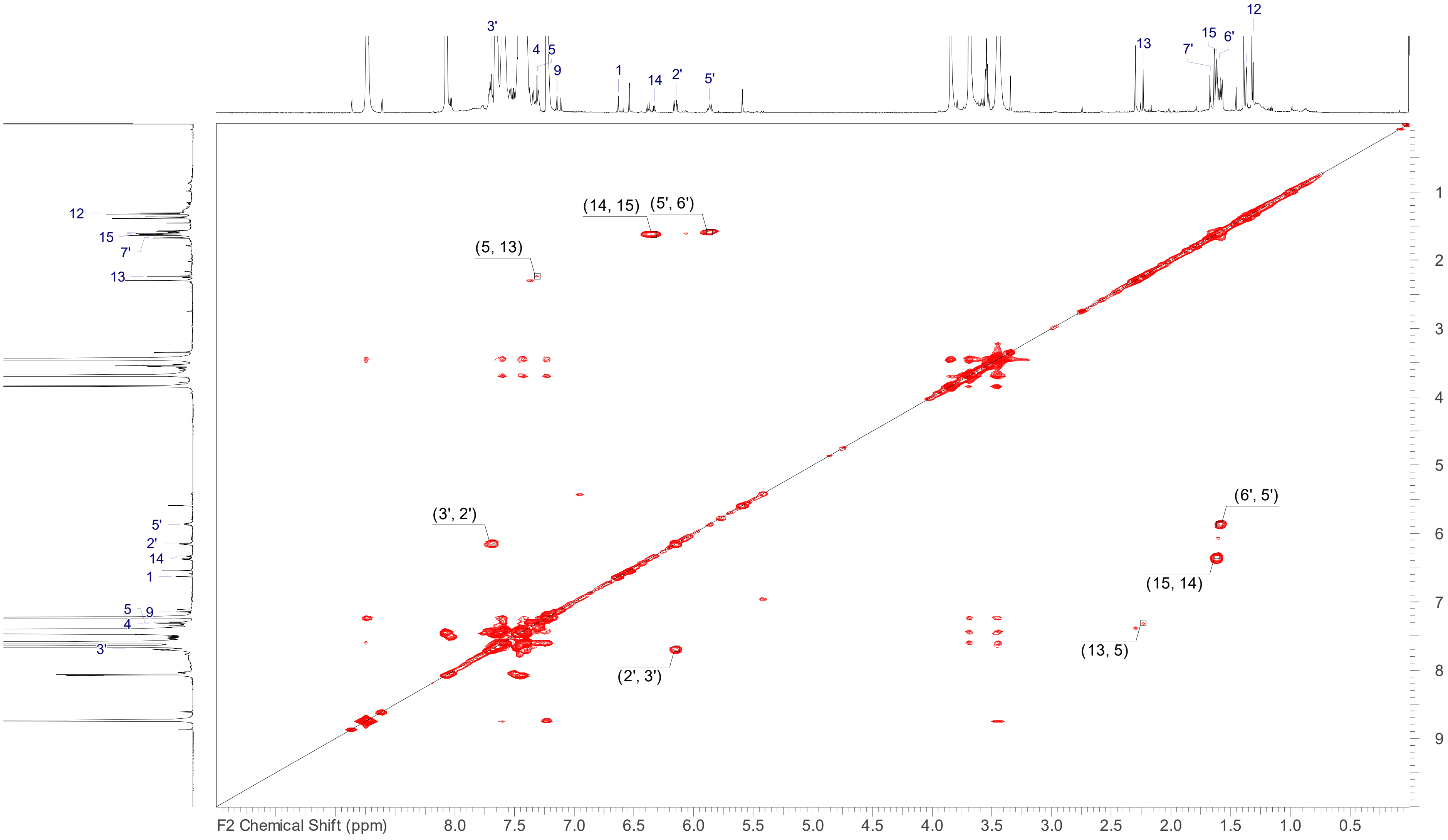

Figure S4. COSY NMR spectrum (700 MHz, pyridine-d_5) of the *S*-MTPA ester of heimionone A (**1**).

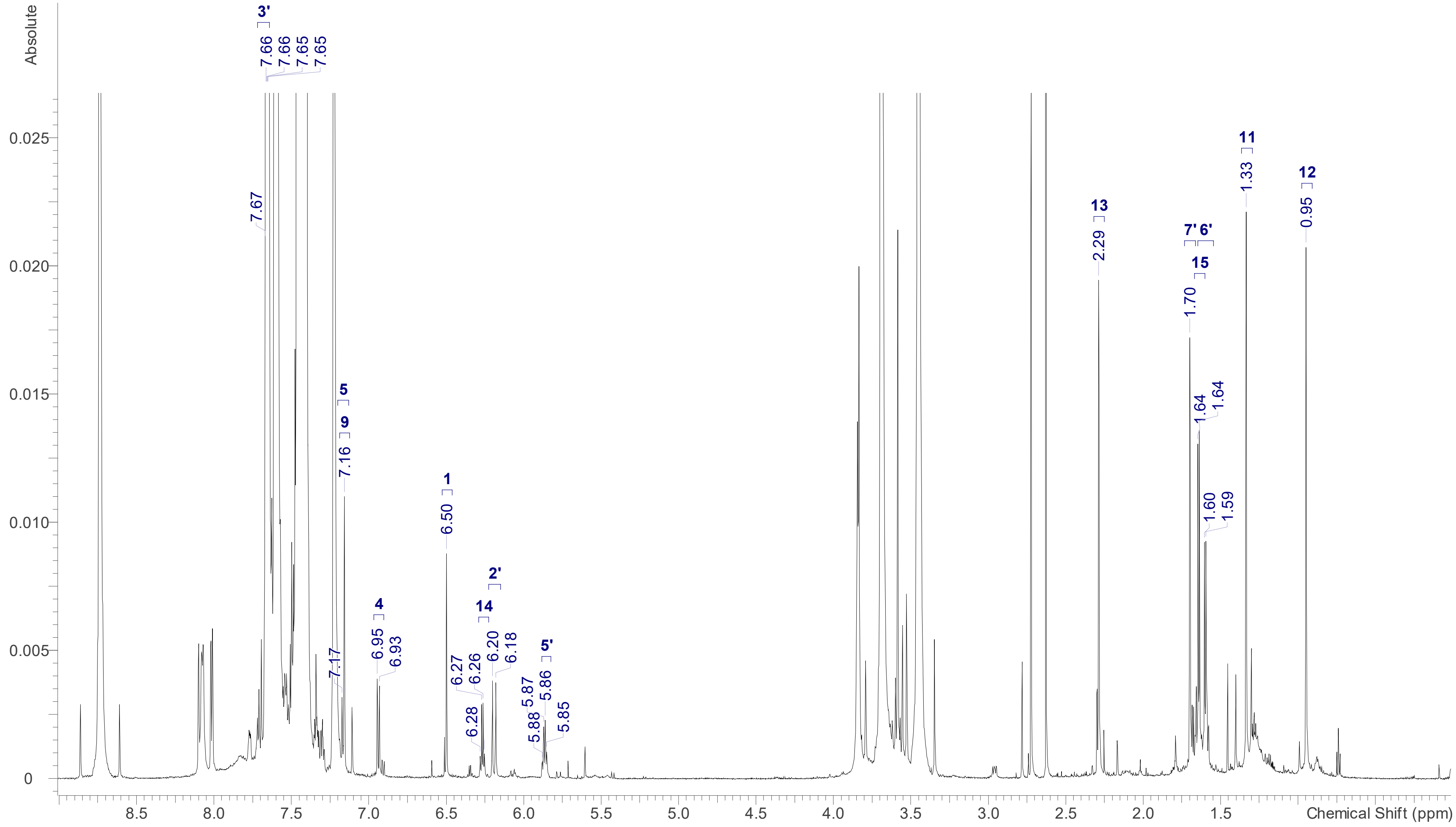

Figure S5. ^{1}H NMR spectrum (700 MHz, pyridine-d_5) of the *R*-MTPA ester of heimionone A (**1**).

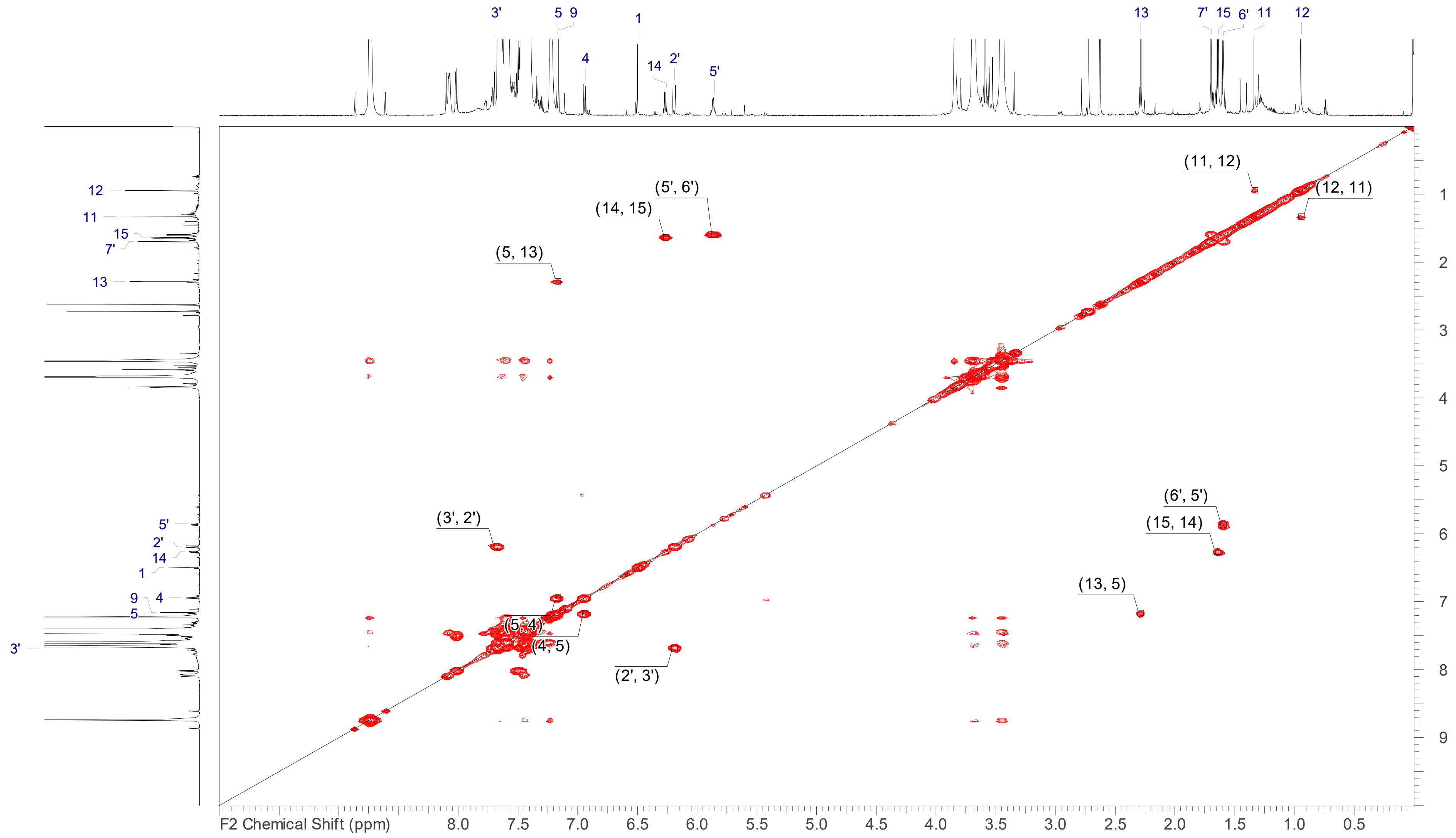

Figure S6. COSY NMR spectrum (700 MHz, pyridine-d_5) of the *R*-MTPA ester of heimionone A (**1**).

Table S2. ^{1}H NMR data (700 MHz, Pyridine-d_5, δ in ppm) of (*S*)/(*R*) MTPA esters obtained from heimionone C (**3**).

**atom**	**heimionone C (3)**	**(*S*)-MTPA ester**	**(*R*)-MTPA ester**	**Δδ*SR***
**1**	6.05	6.07	6.01	0.06
**3**	2.88	2.86	2.86	0.00
**4α**	1.47	1.46	1.43	0.03
**4β**	1.64	1.65	1.64	0.01
**5α**	2.68	2.43	2.45	-0.02
**5β**	1.90	1.94	1.93	0.01
**6**	6.29	6.30	6.36	-0.06
**10**	5.56	6.96	6.95	0.01
**12**	1.33	1.33	1.31	0.02
**13**	1.36	1.22	0.97	0.25
**14**	2.04	1.96	2.01	-0.05
**15**	1.08	1.04	1.03	0.01

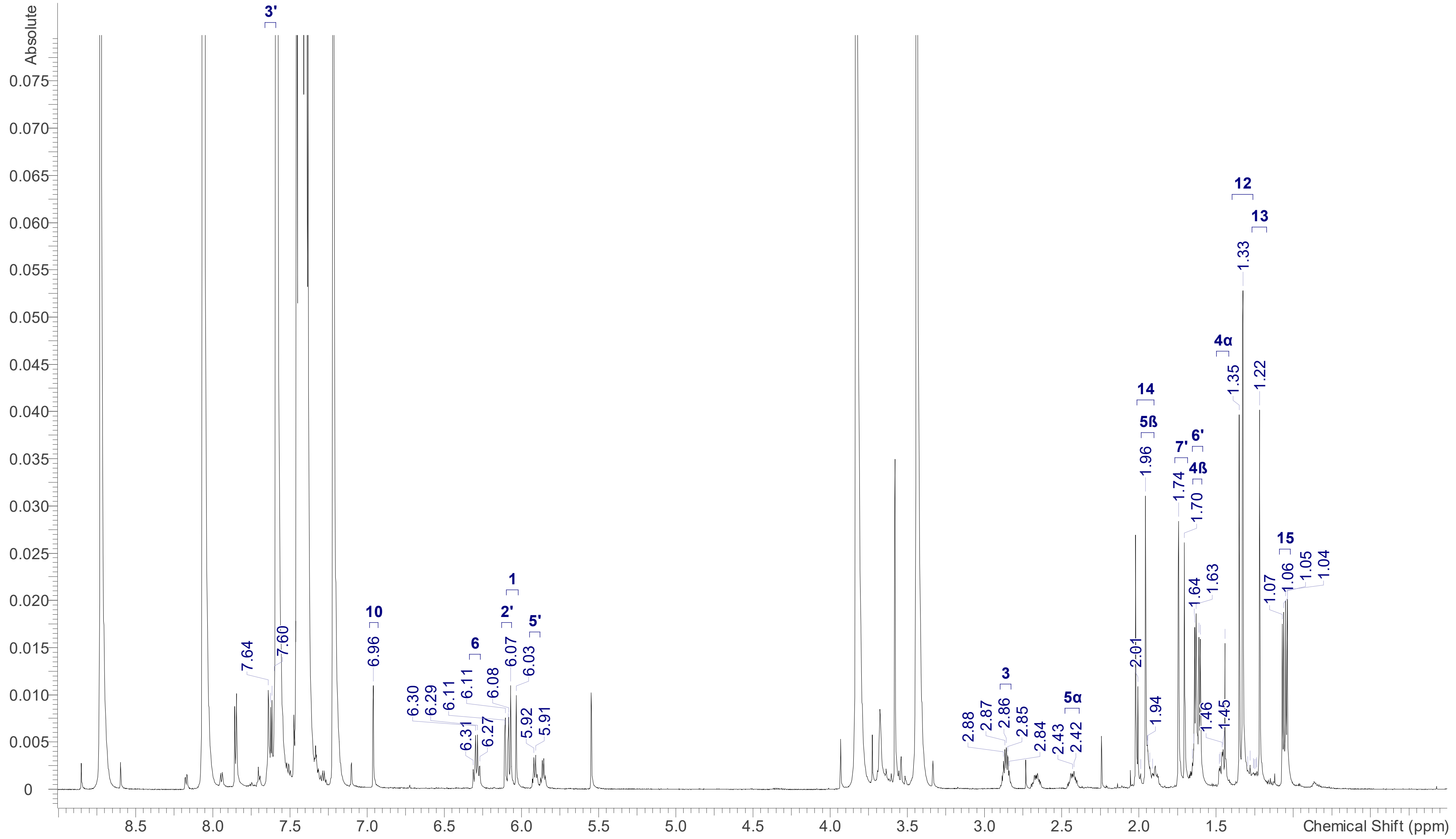

Figure S7. ^{1}H NMR spectrum (700 MHz, pyridine-d_5) of the *S*-MTPA ester of heimionone C (**3**).

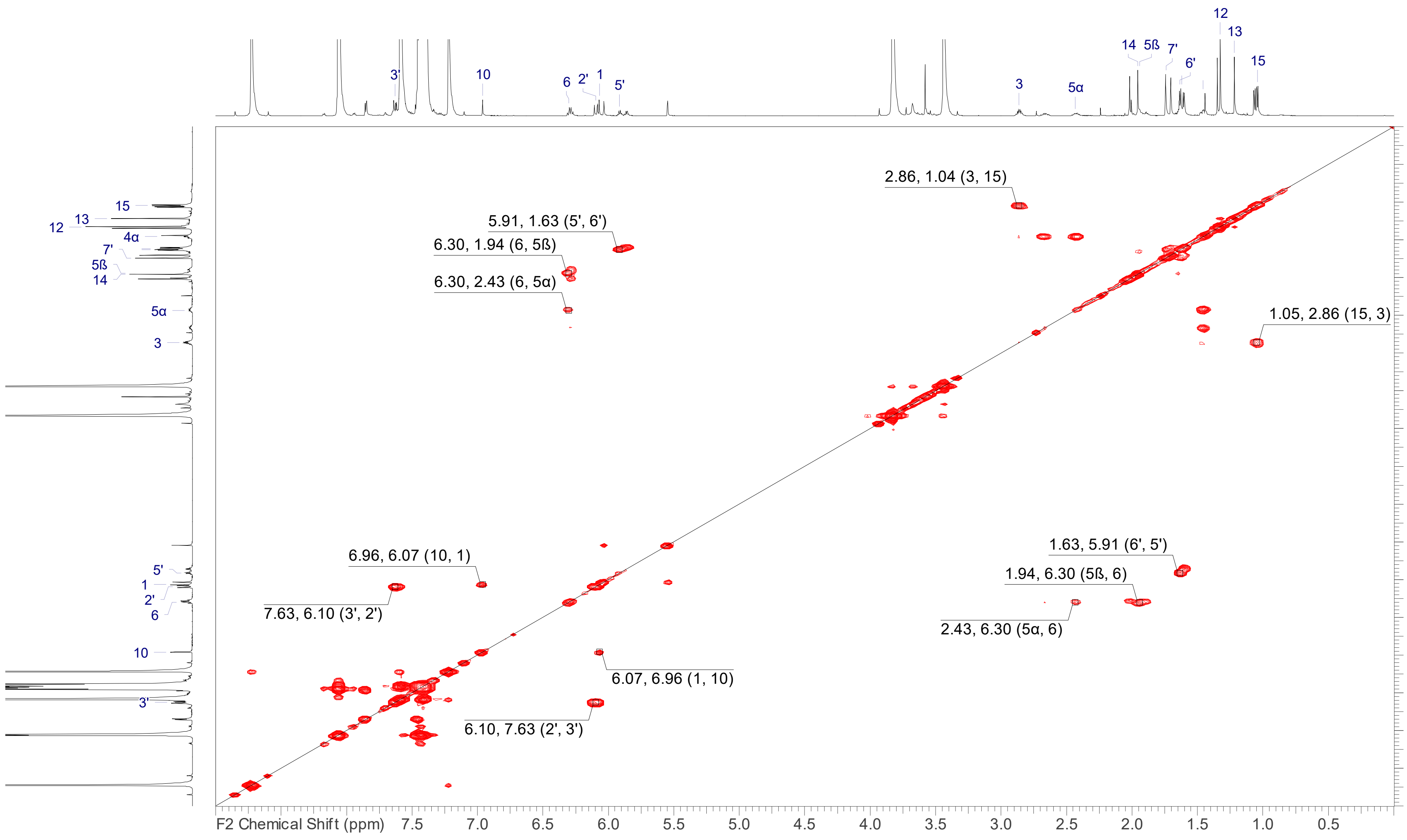

Figure S8. COSY NMR spectrum (700 MHz, pyridine-d_5) of the *S*-MTPA ester of heimionone C (**3**).

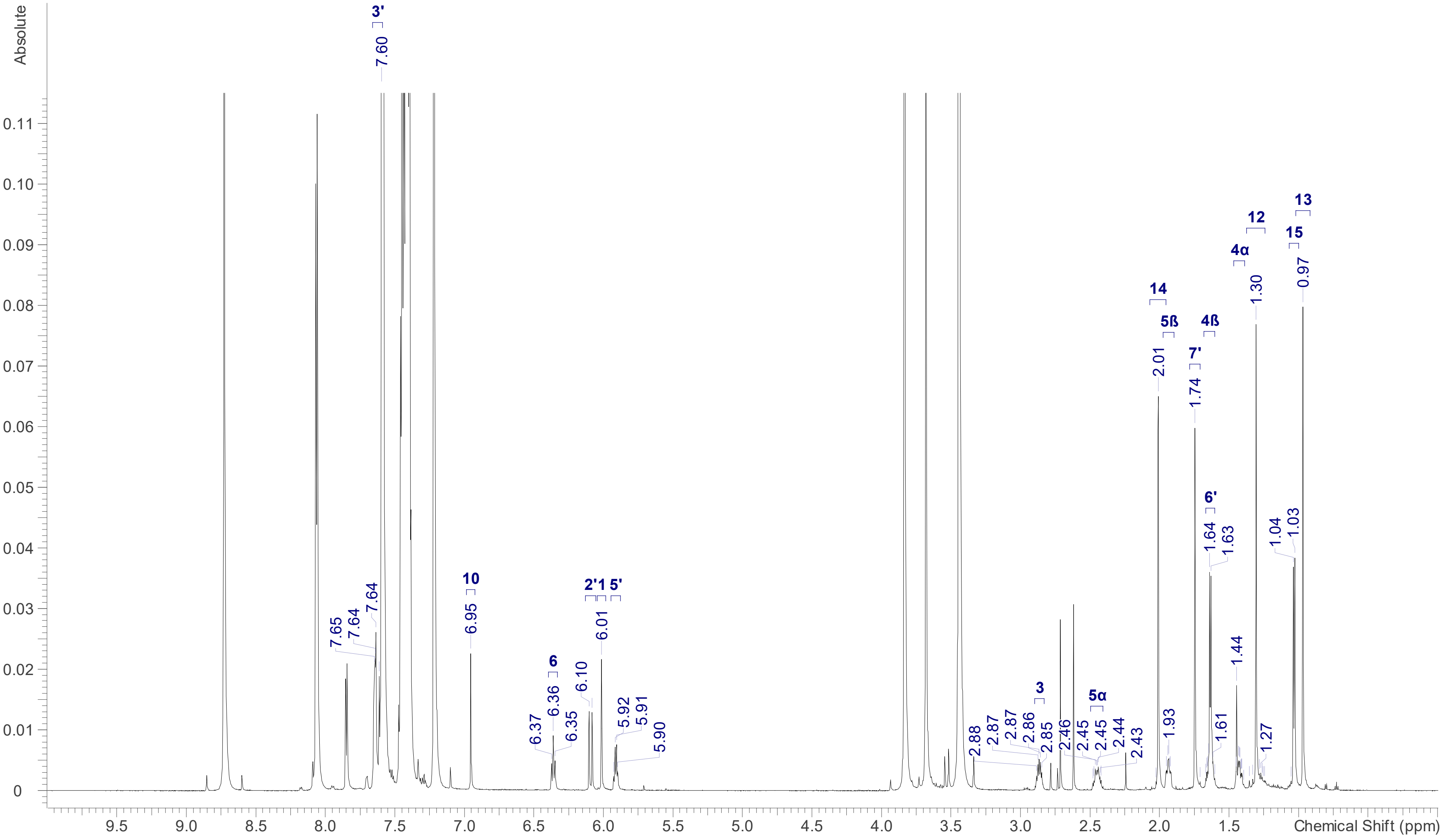

Figure S9. ^{1}H NMR spectrum (700 MHz, pyridine-d_5) of the *R*-MTPA ester of heimionone C (**3**).

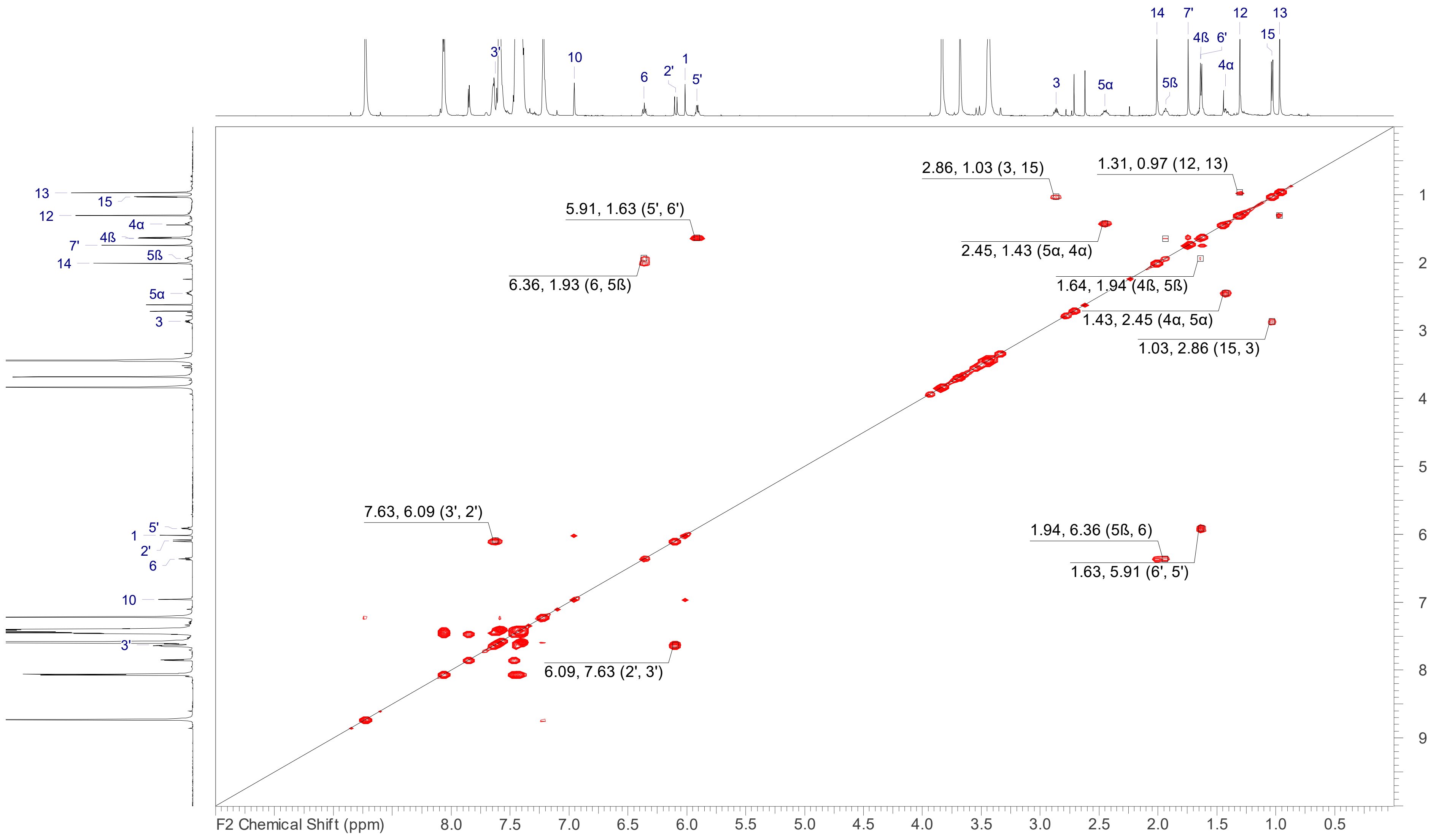

Figure S10. COSY NMR spectrum (700 MHz, pyridine-d_5) of the *R*-MTPA ester of heimionone C (**3**).

Table S3. ^{1}H NMR data (700 MHz, Pyridine-d_5, δ in ppm) of (*S*)/(*R*) MTPA esters obtained from heimionone E (**5**).

atom	heimionone E (5)	(*S*)-MTPA ester	(*R*)-MTPA ester	Δδ*SR*
**1**	4.36	5.89	5.97	-0.08
**3**	3.03	2.84	2.99	-0.15
**4α**	1.66	1.56	1.62	-0.06
**4β**	1.42	1.39	1.42	-0.03
**5α**	2.31	2.22	2.24	-0.02
**5β**	1.91	1.93	1.93	0
**6**	6.13	6.15	6.19	-0.04
**10α**	2.47	2.49	2.46	+0.03
**10β**	3.11	3.10	3.03	+0.07
**12**	1.36	1.20	1.02	+0.17
**13**	0.98	1.07	1.05	+0.02
**14**	2.01	1.95	1.97	-0.03
**15**	1.44	0.81	1.12	-0.31

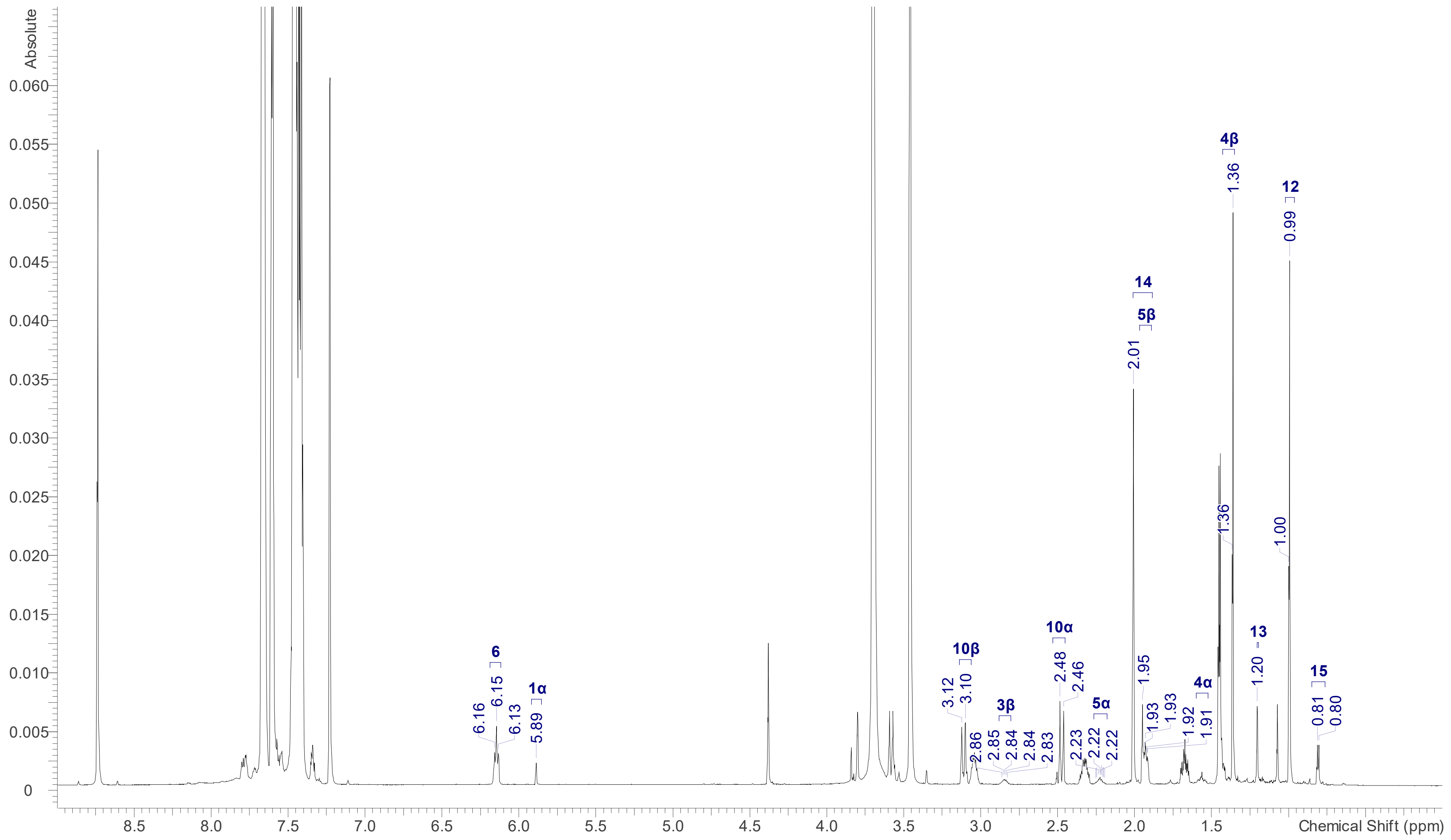

Figure S11. ^{1}H NMR spectrum (700 MHz, pyridine-d_5) of the *S*-MTPA ester of heimionone E (**5**).

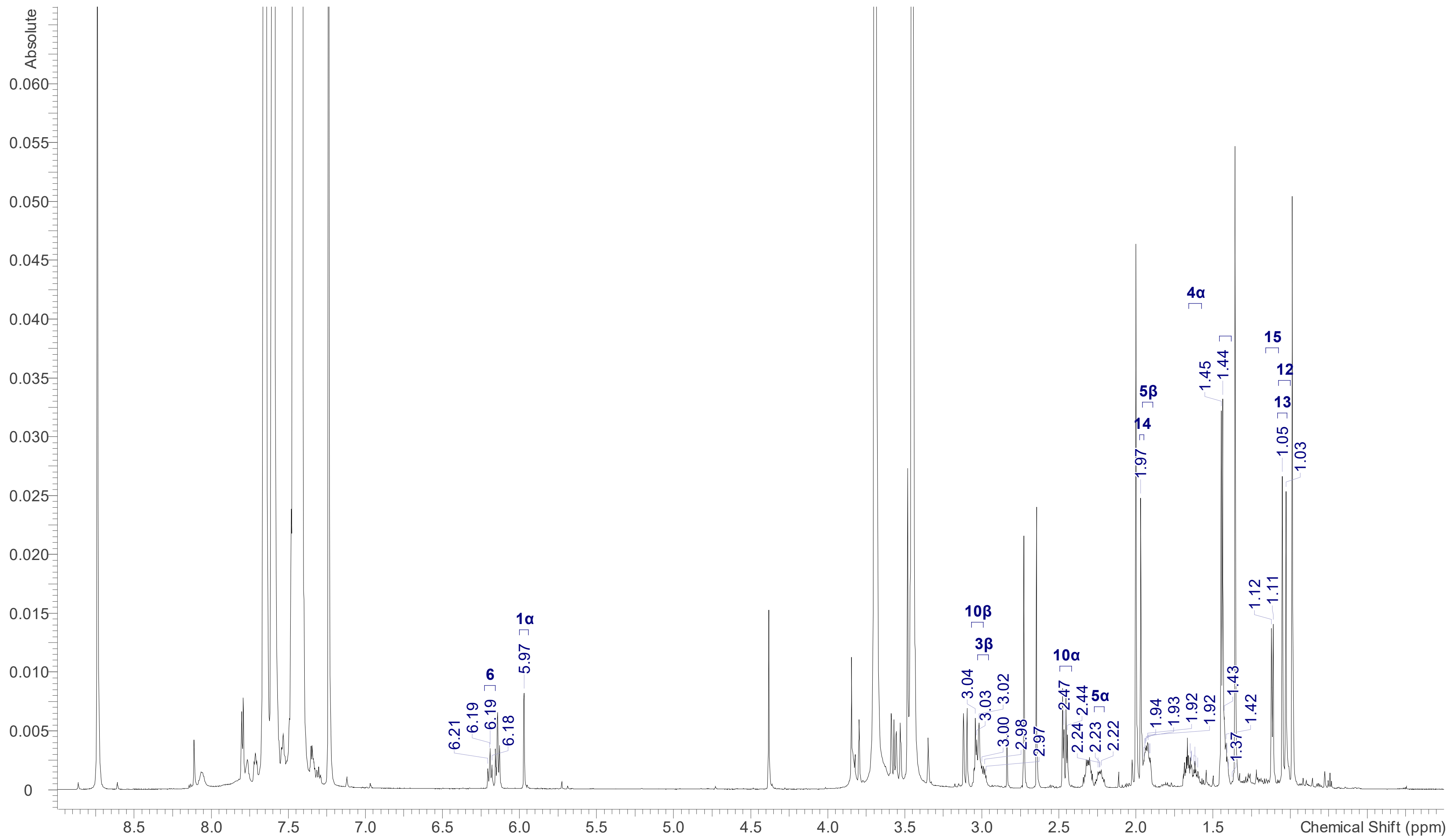

Figure S12. ^{1}H NMR spectrum (700 MHz, pyridine-d_5) of the *R*-MTPA ester of heimionone E (**5**).

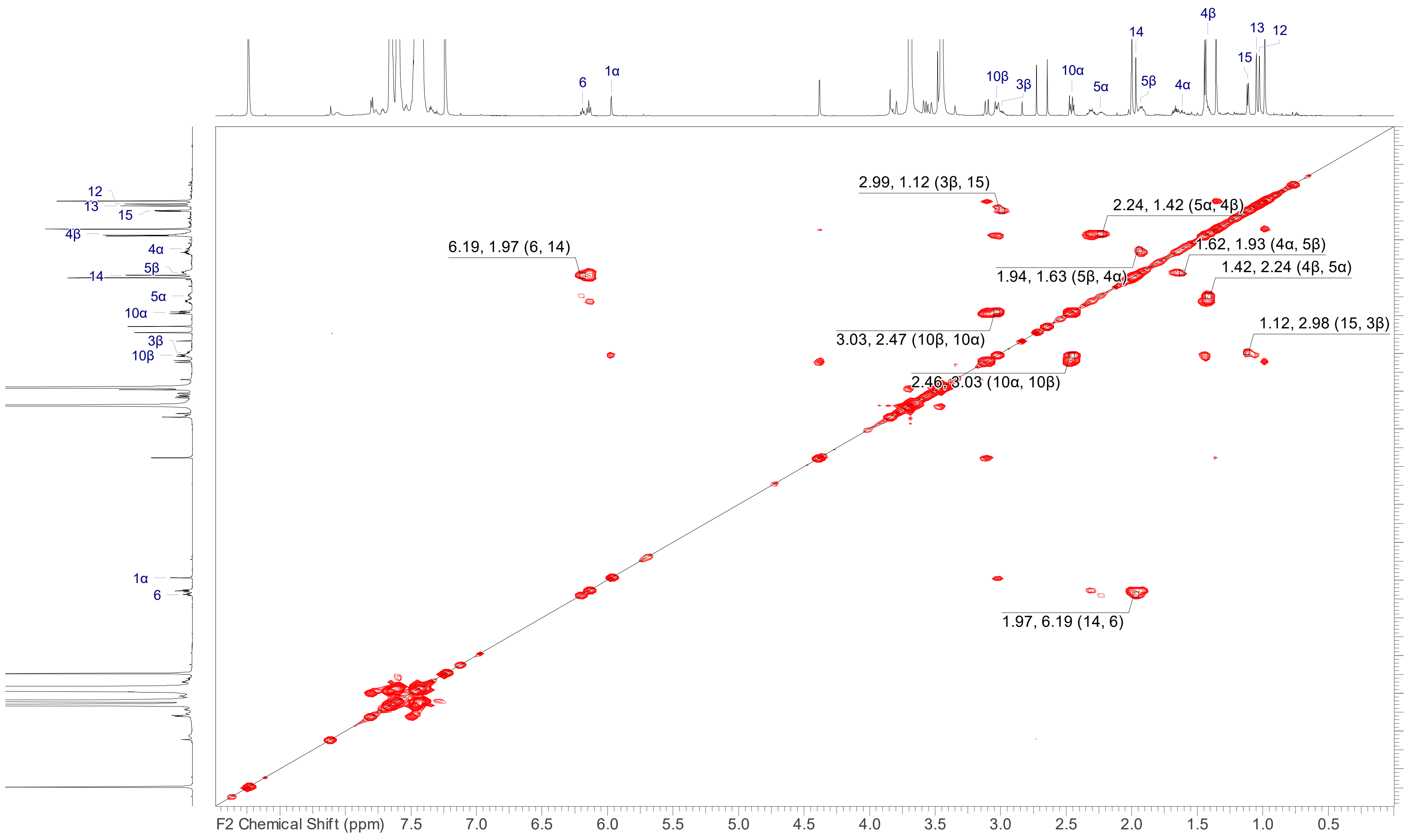

Figure S13. COSY NMR spectrum (700 MHz, pyridine-d_5) of the *R*-MTPA ester of heimionone E (**5**).

1.3. Evaluation of antimicrobial activity.

Table S4. Minimum inhibitory concentration (MIC in µg/mL) for yeast, bacterial and fungal strains.

To perform the serial dilution assay stock solutions (1 mg/mL) of compounds **1**-**5** were used. The antimicrobials [O] Oxytetracyclin, [G] Gentamycin and [K] Kanamycin for bacteria and [N] Nystatin for filamentous fungi and yeast were used as positive controls. No inhibitory effects were observed for negative controls methanol and acetone (20 µL). Compounds **1-5** did not show activities against *Schizosaccharomyces pombe* (DSM 70572), *Pichia anomala* (DSM 6766), *Candida albicans* (DSM 1665), *Escherichia coli* (DSM 1116), *Mycobacterium smegmatis* (ATCC 700084), *Pseudomonas aeruginosa* (DSM PA14), *Chromobacterium violaceum* (DSM 30191) and *Acinetobacter baumannii* (DSM 30008).

**compound**	***Bacillus subtilis* *DSM 10***	***Staphylococcus aureus* *DSM 346***	***Mucor hiemalis* *DSM 2656***	***Rhodotorula glutinis* *DSM 10134***
**1** (heimionone A)	-	-	33.3	66.6
**2** (heimionone B)	-	-	66.6	66.6
**3** (heimionone C)	33.3	66.6	33.3	66.6
**4** (heimionone D)	-	-	-	-
**5** (heimionone E)	66.6	-	-	-
positive control	*8.3 [O]*	*0.83 [O]*	*8.3 [N]*	*4.2 [N]*

IC_{50} in µg/ml; - no activity

1.4. Evaluation of cytotoxicity.

Table S5. Half inhibitory concentration (IC_{50} in µM).

To perform the cytotoxicity assay stock solutions (1 mg/mL) of all compounds were used. Epothilon B was tested as positive control. Negative controls methanol and acetone (20 µL) did not show inhibitory effects.

**compound**	**KB3.1**	**L929**
**1** (heimionone A)	19.4	34.9
**2** (heimionone B)	59.1	45.7
**3** (heimionone C)	19.6	39.1
**4** (heimionone D)	-	-
**5** (heimionone E)	-	-
Epo B (positive control)	*5,5 × 10^{-5}*	*5,8 × 10^{-3}*

IC_{50} in µM; - no activity

2. Structures of known compounds.

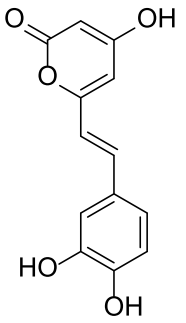

6

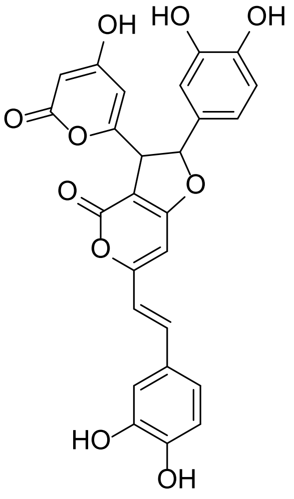

7

Figure S14. Previously described compounds that were observed in submerged cultures of *Heimiomyces* sp. **6**: hispidin, **7**: hypholomin B.

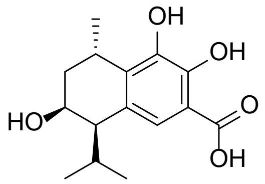

8

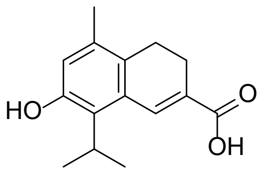

9

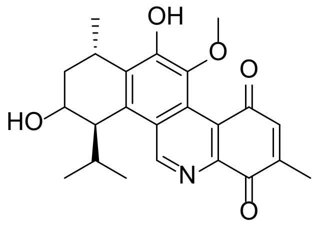

10 (original structure)

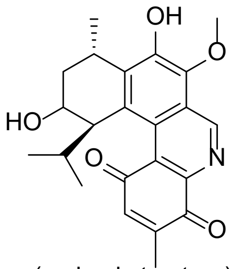

10 (revised structure)

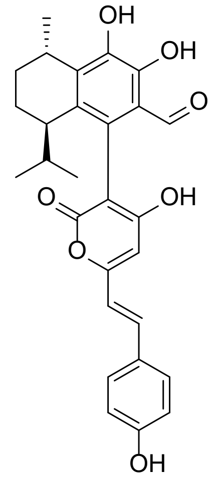

11

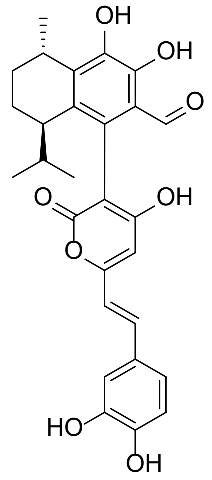

12

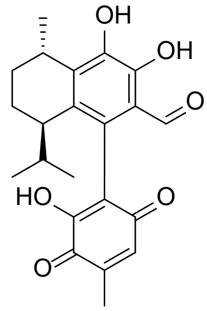

13

Figure S15. Compounds previously isolated from *Heimiomyces* sp.: **8**-**10**: heimiocalamenes C-E (including originally proposed and revised structure of 10), **11**-**13**: heimiomycins A-C.

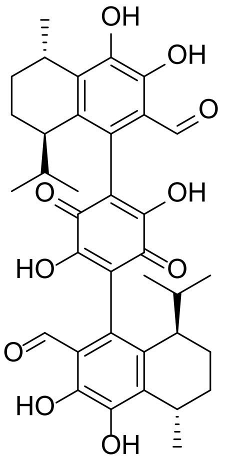

14

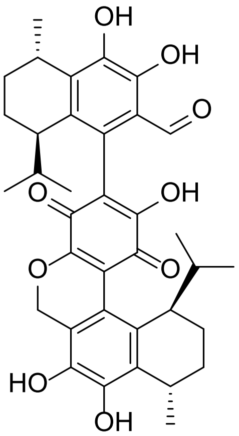

15

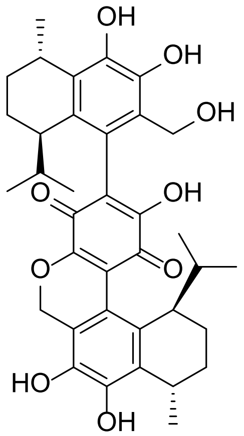

16

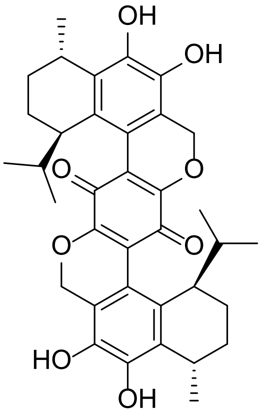

17

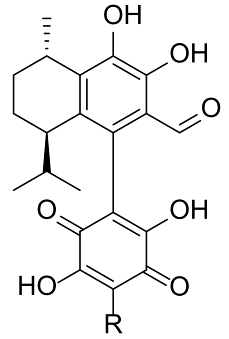

18: R = CH_3
19: R = H

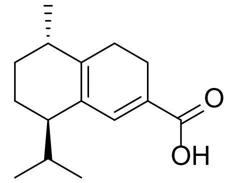

20

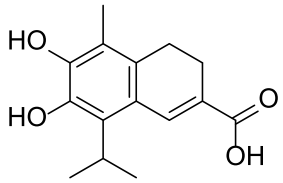

21

Figure S16. Compounds previously isolated from *Heimiomyces* sp. **14**-**17**: bis-heimiomycins A-D, **18**-**19**: heimiomycins D-E, **20**-**21**: heimiocalamenes A-B.

3. NMR spectroscopic data of 1-5.

Table S6. NMR spectroscopic data (^{13}C (δ_C), 175 MHz and ^{1}H (δ_H), 500 MHz, methanol-d_4) for heimionone A (**1**).

No.	δ_C, type	δ_H (J in Hz)	COSY	C to H HMBC	N/ROESY
1	83.5, CH	α: 6.12, s	11, 9β	11, 10, 9, 4, 8, 2, 1'	12, 14β
2	153.0, C				
3	152.4, C				
4	131.8, CH	7.56, d (9.8)	13, 14β, 5	15, 14, 5, 8, 6	
5	138.6, CH	7.69, dd (9.8, 0.9)	13, 4	13, 4, 6, 7	
6	151.7, C				
7	187.5, C				
8	146.1, C				
9	84.2, CH	β: 5.14, s	1α	11, 10, 8, 2, 7	11
10	45.5, C				
11	20.5, CH_3	1.13, s	12, 1α	12, 10, 1	9β
12	20.7, CH_3	1.02, s	11	11, 10, 1, 9	1α
13	22.3, CH_3	2.31, br s	4, 5	4, 5, 8, 6, 7	
14	67.8, CH	ß: 4.70, q (6.3)	15, 4	15, 4, 8, 3	1α
15	26.1, CH_3	1.40, d (6.3)	14β	14, 3	
1'	168.6, C				
2'	115.1, CH	5.81, d (15.7)	7', 3'	4', 5', 1'	7'
3'	152.6, CH	7.35, d (15.7)	2'	7', 2', 4', 5', 1'	5'
4'	135.3, C				
5'	139.2, CH	6.07, q (7.0)	6'	7', 6', 3'	3'
6'	14.8, CH_3	1.82, d (7.0)	5'	7', 4', 5', 3'	
7'	11.9, CH_3	1.77, m	2'	6', 4', 3'	2'

Table S7. NMR spectroscopic data (^{13}C (δ_C), 175 MHz and ^1H (δ_H), 700 MHz, methanol-d_4) for heimionone B (**2**).

No.	δ_C, type	δ_H (J in Hz)	COSY	C to H HMBC	N/ROESY
1	86.1, CH	α: 6.32, m		12, 10, 9, 8, 2, 1'	12
2	146.2, C				
3	133.5, C				
4	134.8, C	6.26, d (11.8)	5	6, 3, 14, 2	
5	130.5, CH	5.53, d (11.8)	4	13, 6, 7	
6	78.7, C				
7	199.3, C				
8	142.2, C				
9	83.4, CH	β: 4.61, s		11, 10, 1, 8, 2	11, 13
10	47.2, C				
11	22.0, CH_3	0.98, m	12	12, 10, 9, 1	13, 9β
12	21.7, CH_3	1.18, m	11	11, 10, 9, 1	1α
13	27.2, CH_3	1.45, m		6, 5, 7	11, 9β
14	141.6, CH	6.16, q (7.7)	15	15, 4, 2	
15	16.9, CH_3	1.90, d (7.7)	14	3, 4, 14, 2	
1'	168.7, C				
2'	115.3, CH	5.81, d (15.7)	3'	4', 1'	7'
3'	152.2, CH	7.33, d (15.7)	2'	7', 2', 4', 5', 1'	5'
4'	135.3, C				
5'	139.0, CH	6.06, q (7.1)	6'	7', 6', 3'	3'
6'	14.8, CH_3	1.84, d (7.1)	5'	4', 5', 3'	
7'	11.9, CH_3	1.79, m		4', 5', 3'	2'

Table S8. NMR spectroscopic data (^{13}C (δ_C), 125 MHz and ^1H (δ_H), 500 MHz, acetonitrile-d_3) for heimionone C (**3**).

No.	δ_C, type	δ_H (J in Hz)	COSY	C to H HMBC	N/ROESY
1	83.1, CH	α: 5.62, s	10β	13, 3, 11, 10, 2', 9, 2, 1'	13, 4α
2	150.3, C				
3	31.2, CH	β: 2.86, dqd (12.8, 6.9, 6.0)	15	4, 2	4β
4	37.1, CH_2	α: 1.57, m		15, 5, 3	15, 5α, 1α
		β: 1.78, m	5α	5, 3	5β, 3β
5	26.3, CH_2	α: 2.46, m	4α, 6		13, 4α
		β: 2.11, m	6	7	4β
6	140.9, CH	6.47, m	14, 5β, 5α		
7	142.0, C				
8	194.2, C				
9	148.9, C				
10	83.3, CH	β: 4.89, s	1α	12, 13, 11, 9, 2	12
11	44.2, C				
12	20.4, CH_3	1.01, s		13, 11, 10, 9	10β
13	21.4, CH_3	0.98, s		12, 11, 10	5α, 1α
14	20.1, CH_3	1.89, s	6	4, 6, 7, 8	
15	16.7, CH_3	0.95, d (6.9)	3β	5, 3, 4, 2	4α
1'	167.7, C				
2'	115.7, CH	5.79, d (15.7)	3'	4', 1'	7'
3'	150.9, CH	7.30, d (15.7)	2'	7', 2', 4', 5', 1'	5'
4'	134.9, C				
5'	138.4, CH	6.06, q (7.0)	6'	7'	3'
6'	14.8, CH_3	1.80, d (7.0)	5'	4', 5', 3'	
7'	12.0, CH_3	1.77, s		4', 5', 3'	2'

Table S9. NMR spectroscopic data (^{13}C (δ_C), 175 MHz and ^1H (δ_H), 700 MHz, acetonitrile-d_3) for heimionone D (**4**).

No.	δ_C, type	δ_H (J in Hz)	COSY	C to H HMBC	N/ROESY
1	85.6, CH	α: 5.58, s	10β	13, 3, 10, 9, 2, 1'	15, 13, 4α
2	150.3, C				
3	31.2, CH	β: 2.96, m	15	15, 4, 1, 9, 2	4β, 5β
4	37.3, CH_2	α: 1.53, m		15, 5, 3, 6	15, 5α, 1α
		β: 1.74, m		15, 5, 3	3β
5	26.3, CH_2	α: 2.07, m	6		4α
		β: 2.32, m	6	4, 6, 7	3β
6	139.1, CH	6.35, ddd (9.7, 8.4, 1.4)	14, 5α, 5β	14	
7	141.5, C				
8	194.3, C				
9	146.9, C				
10	47.8, CH_2	α: 2.30, d (16.4)	10β	13, 11, 1, 9, 2	13
		β: 2.77, d (16.4)	10α, 1α	12, 13, 11, 9, 2	12
11	39.4, C				
12	22.2, CH_3	0.99, s		13, 11, 10, 1	10β
13	28.0, CH_3	1.06, s		12, 11, 10, 1	10α, 1α
14	20.3, CH_3	1.88, s	6	6, 7, 8	
15	17.0, CH_3	0.97, d (6.9)	3β	3, 4, 2	4α, 1α
1'	167.6, C				
2'	115.9, CH	5.81, d (15.7)	3'	4', 1'	7'
3'	150.8, CH	7.31, d (15.7)	2'	7', 2', 4', 5', 1'	5'
4'	134.9, C				
5'	138.2, CH	6.06, q (6.9)	6'	7', 6', 3'	3'
6'	14.8, CH_3	1.80, d (6.9)	5'	5'	
7'	12.0, CH_3	1.77, br s		4', 3'	2'

Table S10. NMR spectroscopic data (^{13}C (δ_C), 175 MHz and ^{1}H (δ_H), 700 MHz, methanol-d_4) for heimionone E (**5**).

No.	δ_C, type	δ_H (J in Hz)	COSY	C to H HMBC	N/ROESY
1	85.3, CH	α: 4.13, s	12, 10β	13, 3, 11, 10, 9, 2	13, 15
2	156.5, C				
3	31.9, CH	β: 2.98, dqd (12.5, 6.9, 5.0)	15, 4β, 4α	15, 5, 4, 1, 9, 2	4β
4	38.3, CH_2	α: 1.82, m	4β, 5β, 5α, 3β	15, 5, 3, 6, 2	15
		β: 1.51, m	4α, 5β, 5α, 3β	15, 5, 3, 6, 2	5β, 3β
5	26.7, CH_2	α: 2.30, m	4β, 4α, 5β, 6	14, 3, 4, 6, 7	13
		β: 2.08, m	4β, 4α, 5α, 6	3, 4, 6, 7	4β
6	139.4, CH	6.36, ddd (9.8, 8.4, 1.4)	14, 5β, 5α	14, 5, 7, 8	
7	142.1, C				
8	196.3, C				
9	144.7, C				
10	47.2, CH_2	α: 2.21, d (16.1)	12, 10β	13, 11, 1, 9, 2, 8	13
		β: 2.76, d (16.1)	13, 10α, 1α	12, 13, 3, 11, 1, 9, 2	12
11	39.6, C				
12	22.5, CH_3	1.13, s	13, 10α, 1α	13, 11, 10, 1, 9	10β
13	28.3, CH_3	0.96, s	12, 10β	12, 11, 10, 1	10α, 5α, 1α
14	20.3, CH_3	1.90, s	6	5, 4, 6, 7, 8	
15	18.0, CH_3	1.26, d (6.9)	3β	5, 3, 4, 2	4α, 1α

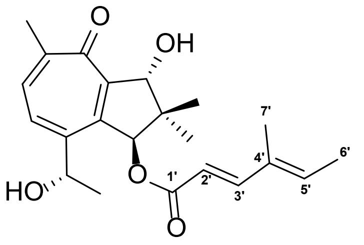

heimionone A (**1**)

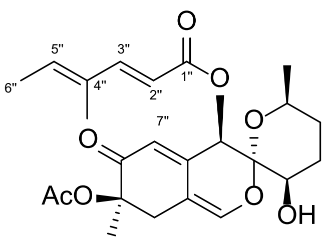

daldinin F

Figure S17. Chemical structures of heimionone A (**1**) and daldinin F [1].

Table S11. Comparison of the NMR spectroscopic data for the 4-methylhexa-2,4-dienoic acid partial structure of heimionone A (**1**) (^{13}C (δ_C), 175 MHz and ^1H (δ_H), 500 MHz, methanol-d_4) and daldinin F (^{13}C (δ_C), 150 MHz and ^1H (δ_H), 600 MHz, chloroform-d_1) [1].

	**heiminone A (1)**		**daldinin F**	
No.	δ_C , type	δ_H (J in Hz)	δ_C , type	δ_H (J in Hz)
1'/1''	168.6, C		167.6, C	
2'/2''	115.1, CH	5.81, d (15.7)	112.9, CH	5.75, d (15.7)
3'/3''	152.6, CH	7.35, d (15.7)	141.5, CH	7.37, d (15.7)
4'/4''	135.3, C		133.8, C	
5'/5''	139.2, CH	6.07, q (7.0)	139.3, CH	6.07, q (7.1)
6'/6''	14.8, CH_3	1.82, d (7.0)	14.8, CH_3	1.83, d (7.1)
7'/7''	11.9, CH_3	1.77, m	11.7, CH_3	1.75, s

4. Literature

[1] Quang, D.N.; Hashimoto, T.; Tanaka, M.; Stadler, M.; Asakawa, Y. Cyclic Azaphilones Daldinins E and F from the Ascomycete Fungus *Hypoxylon Fuscum* (Xylariaceae). Phytochemistry 2004, 65, 469–473

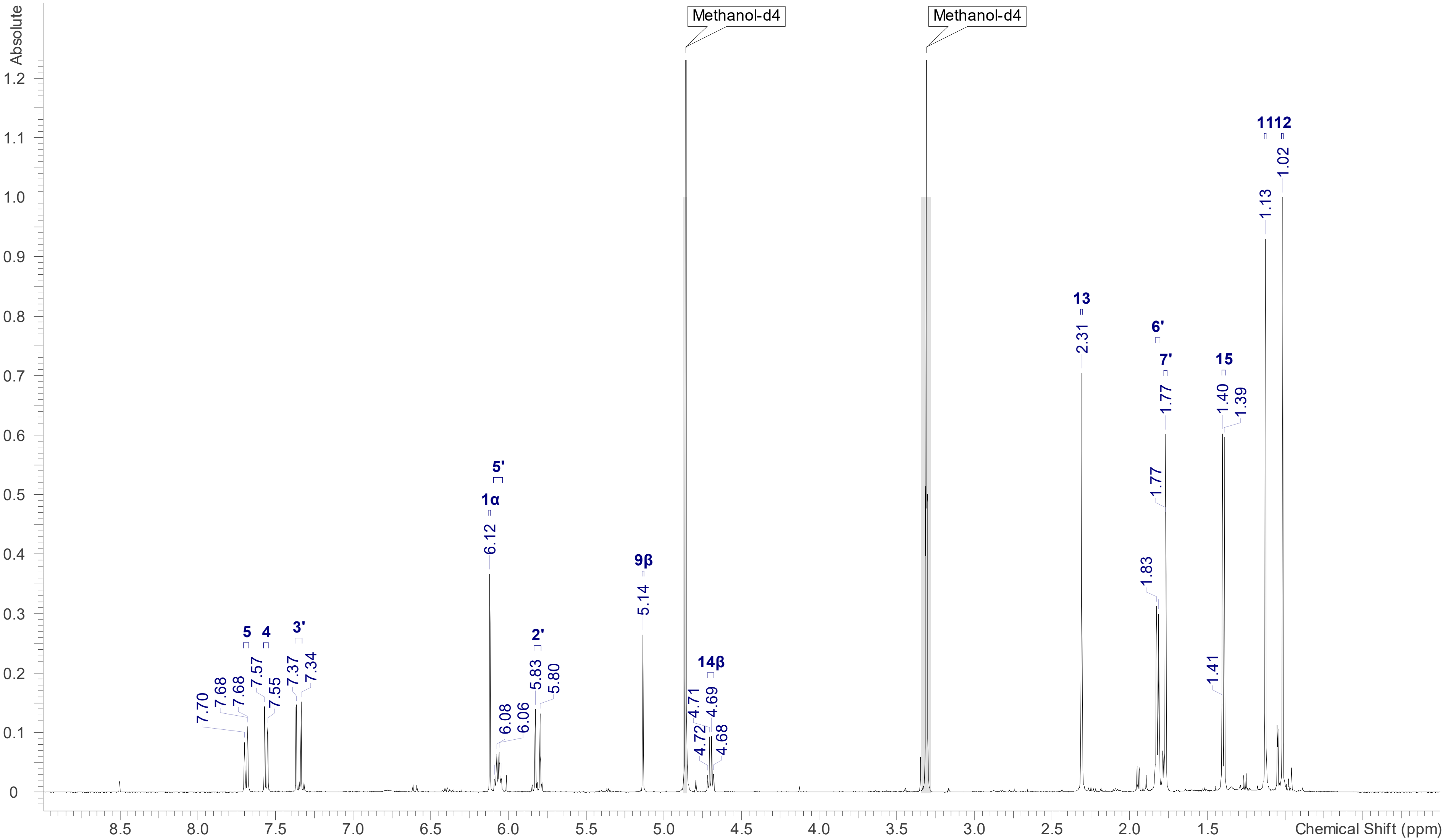

Figure S18. ^{1}H NMR spectrum (500 MHz, methanol-d_4) of heimionone A (**1**).

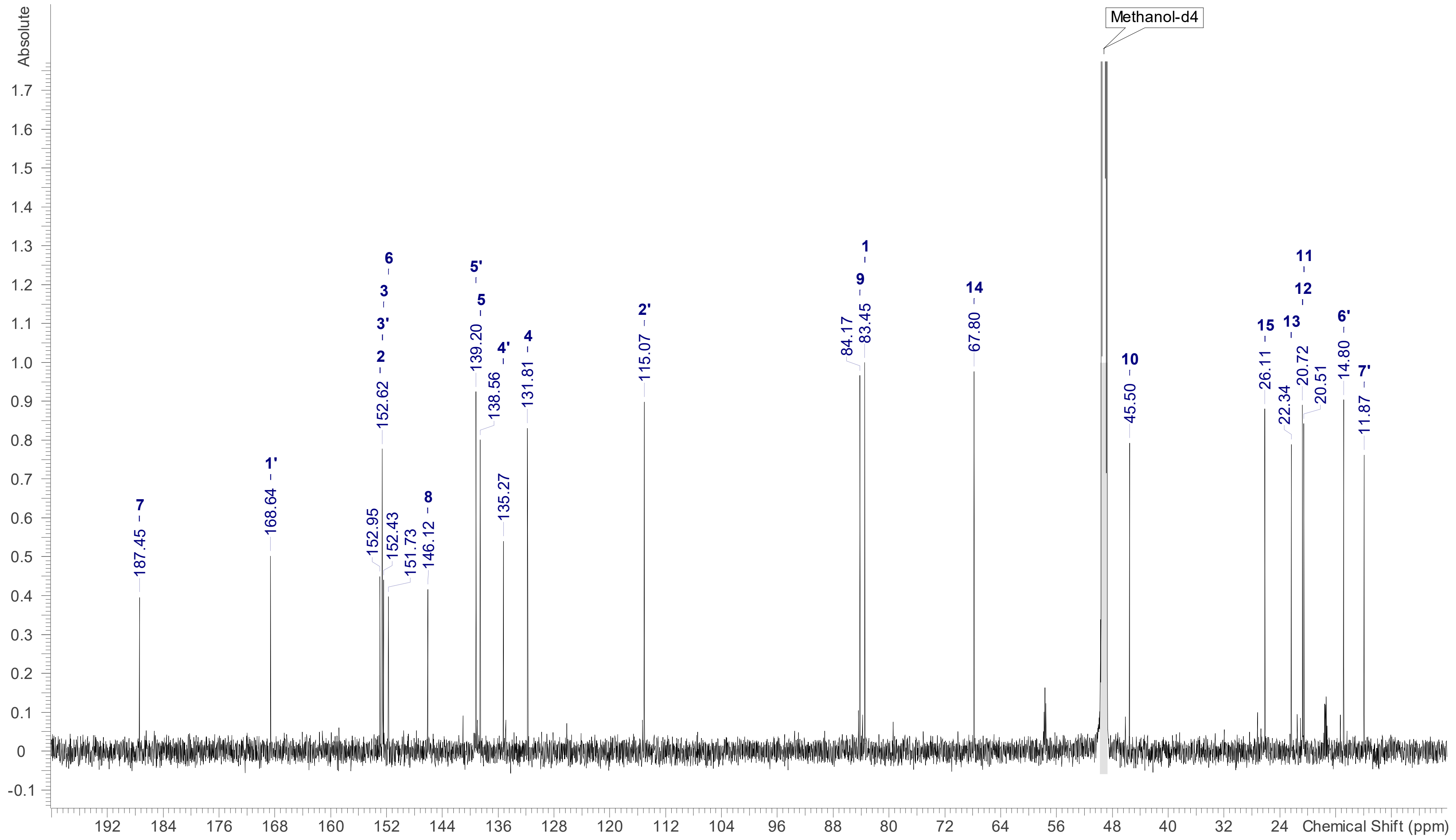

Figure S19. ^{13}C NMR spectrum (175 MHz, methanol-*d*$_4$) of heimionone A (**1**).

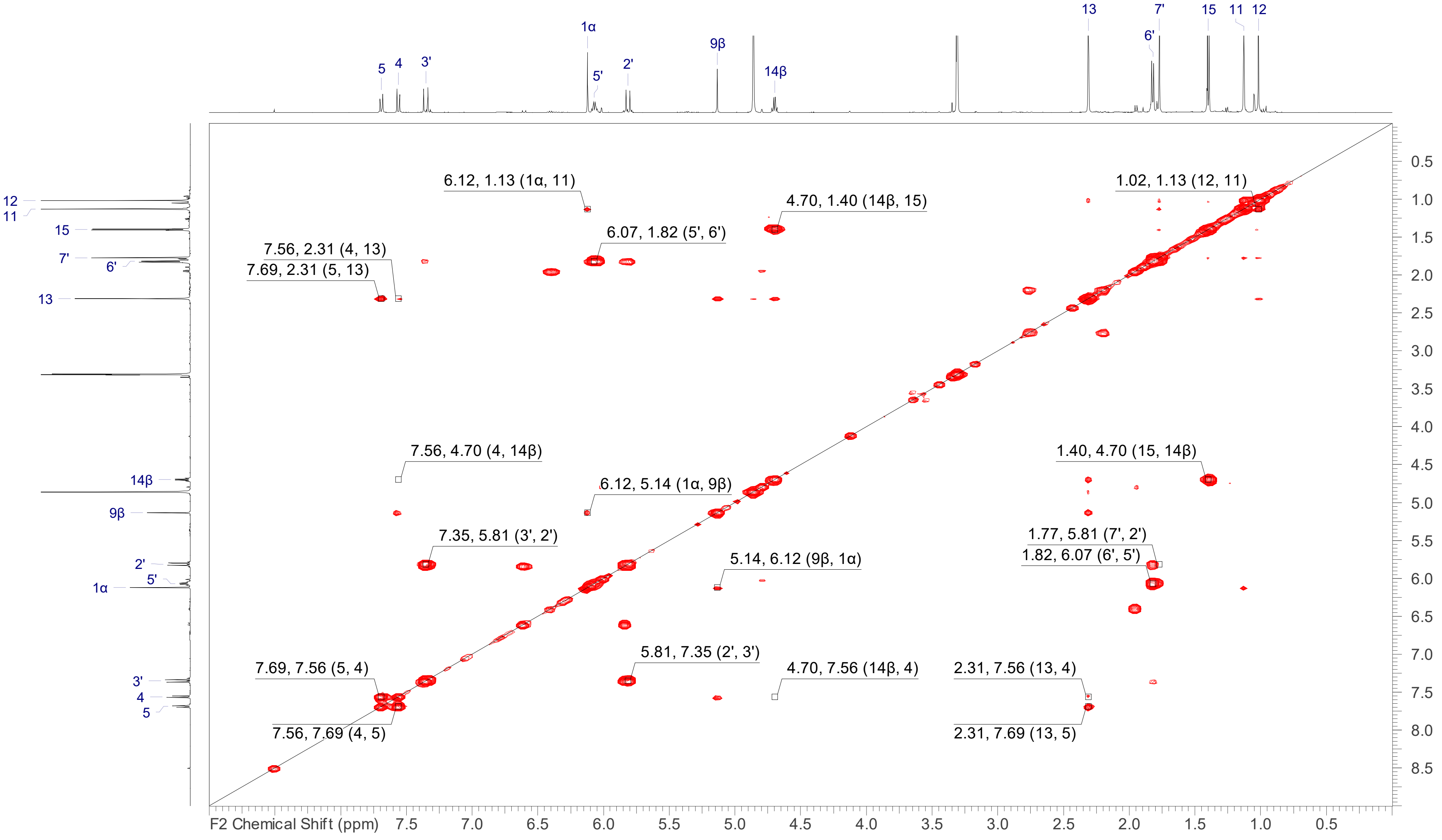

Figure S20. COSY NMR spectrum (500 MHz, methanol-d_4) of heimionone A (**1**).

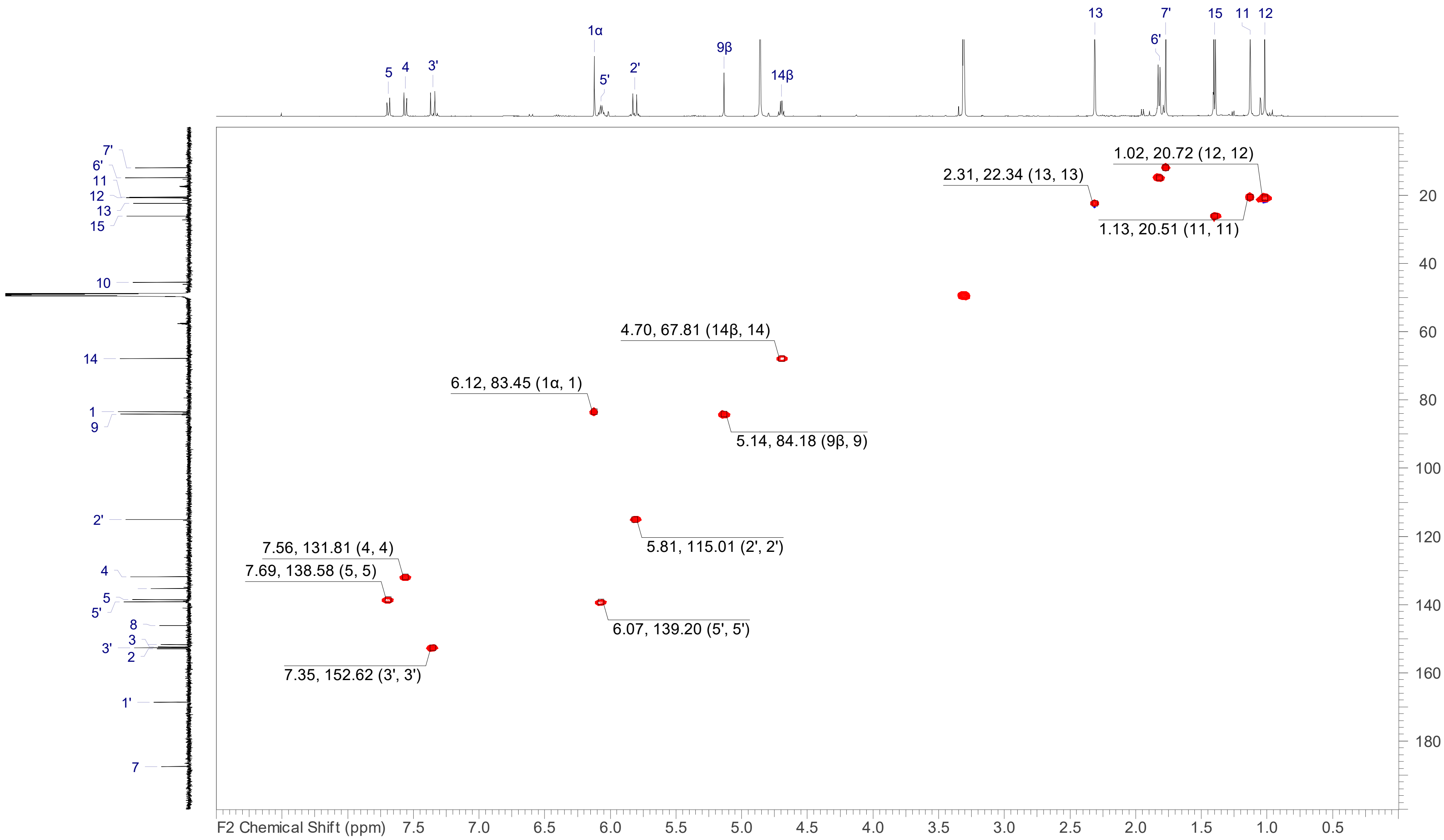

Figure S21. HSQC NMR spectrum (500 MHz, methanol-d_4) of heimionone A (**1**).

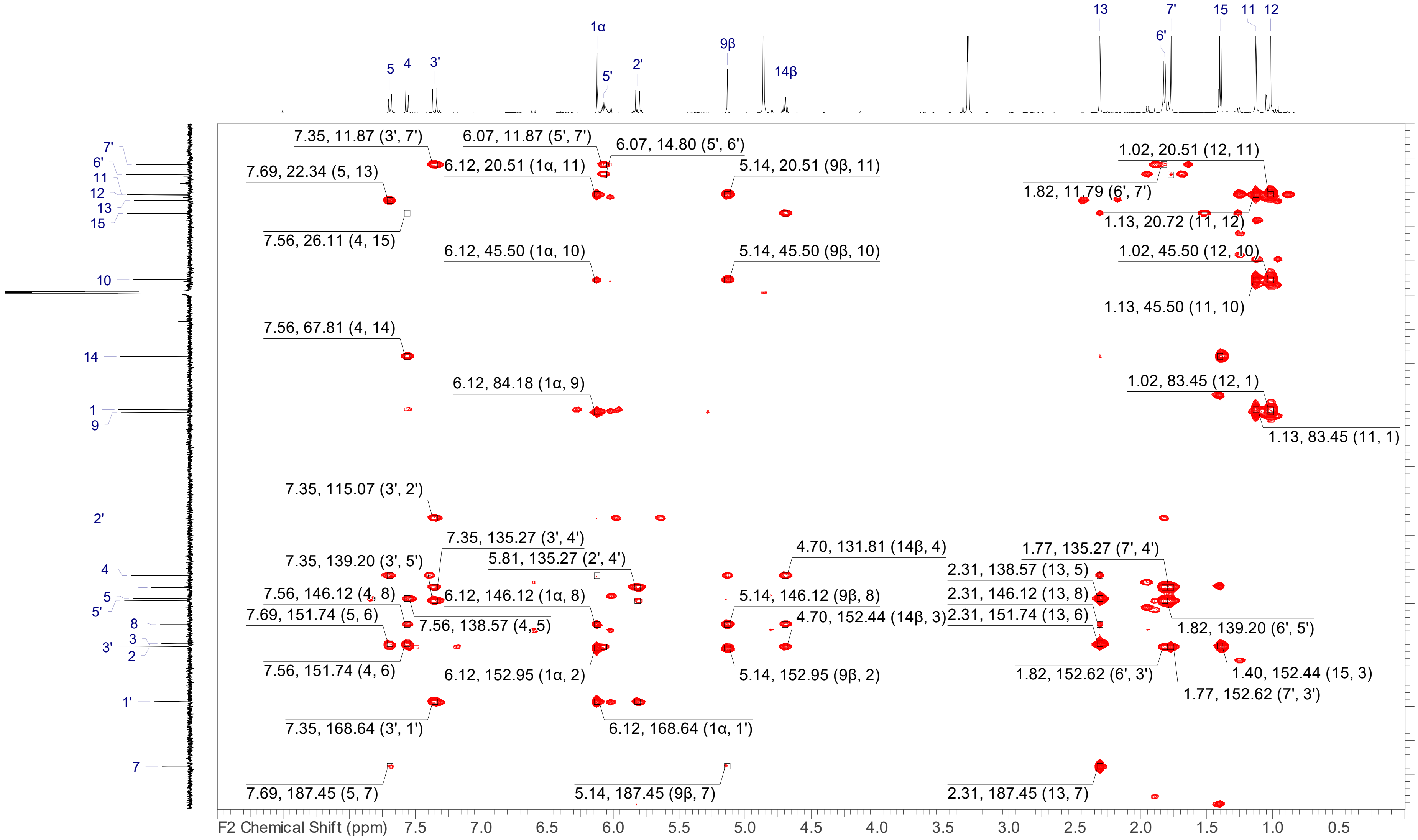

Figure S22. HMBC NMR spectrum (500 MHz, methanol-d_4) of heimionone A (**1**).

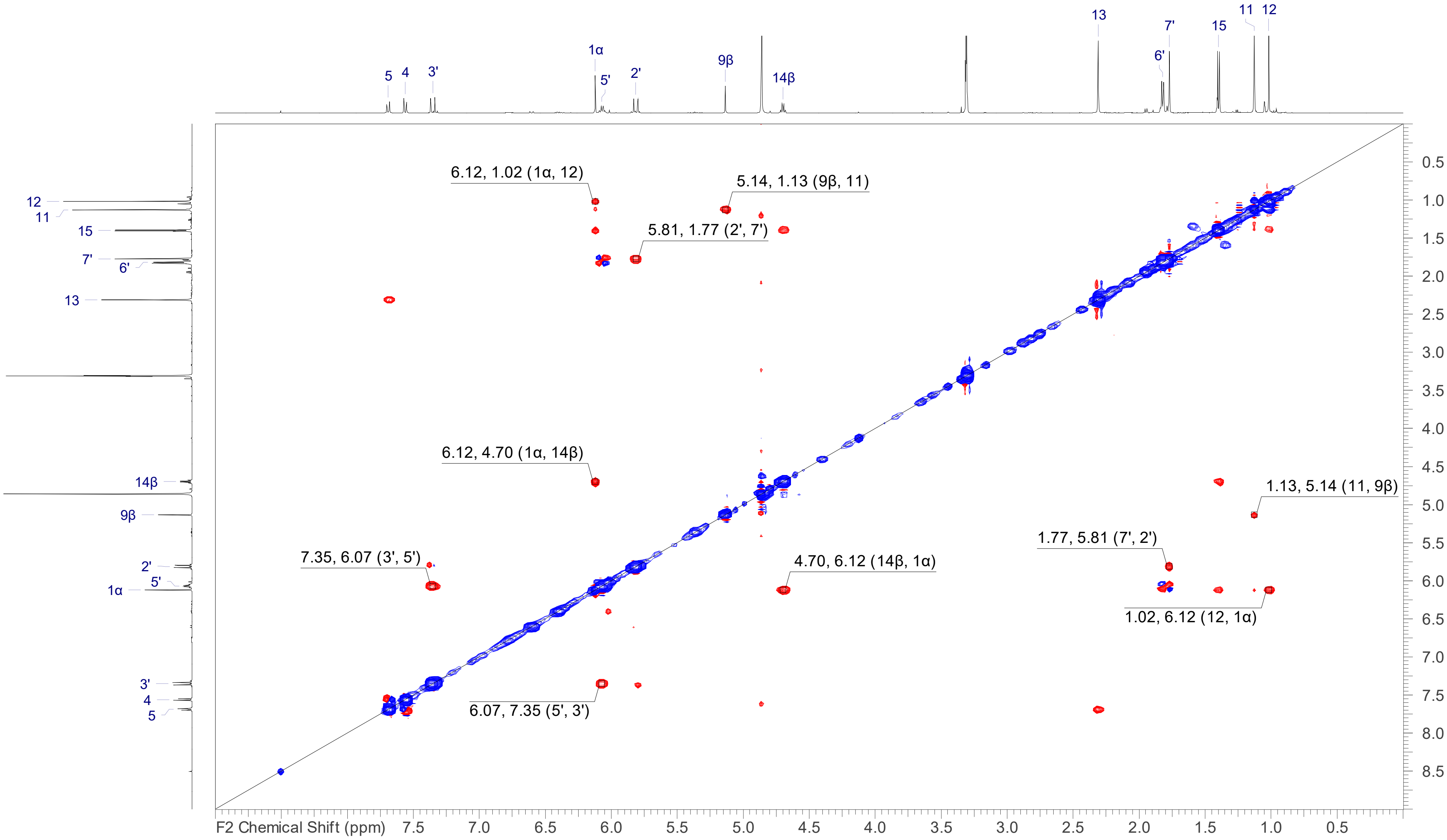

Figure S23. ROESY NMR spectrum (500 MHz, methanol-d_4) of heimionone A (**1**).

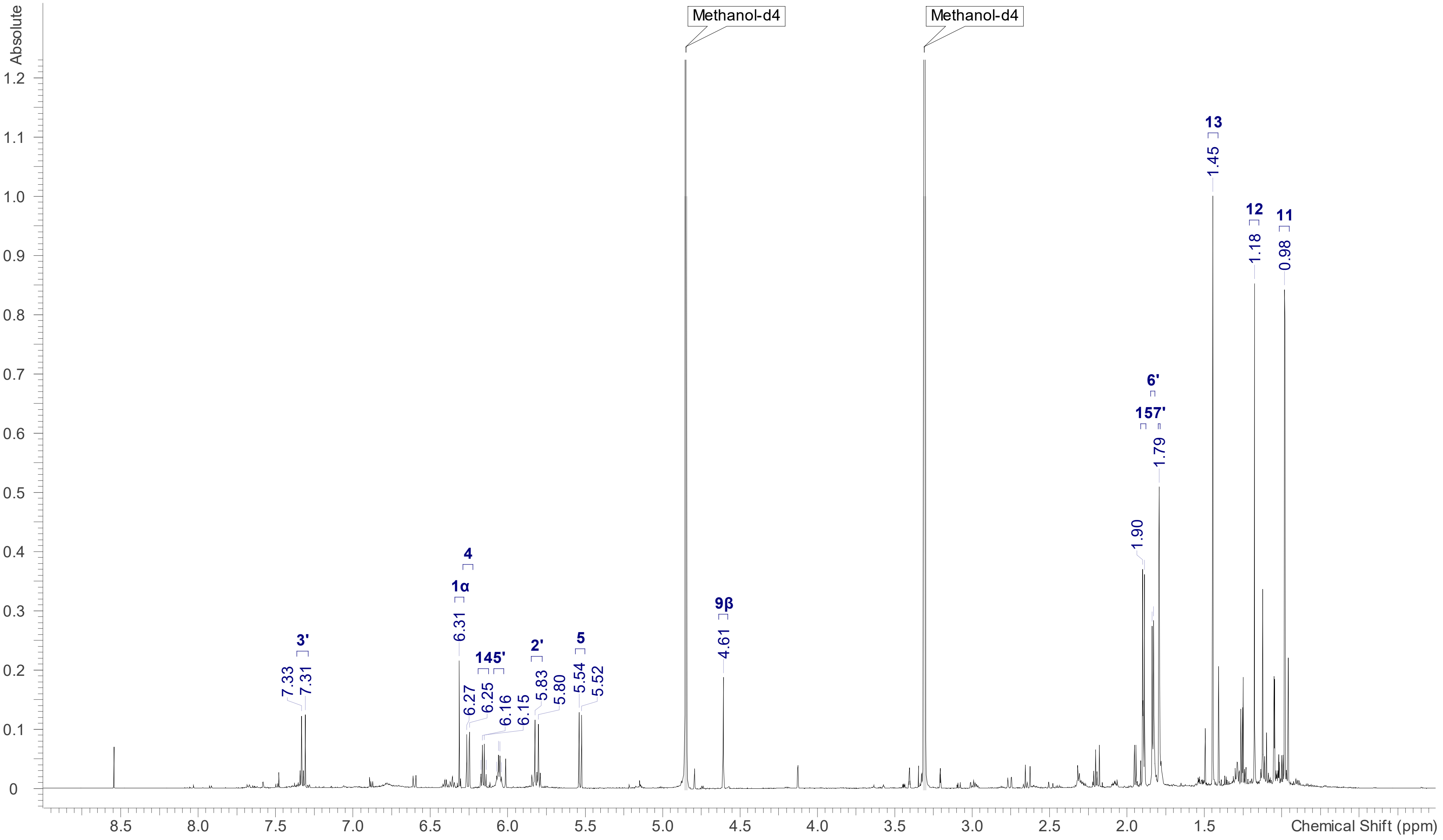

Figure S24. ^{1}H NMR spectrum (700 MHz, methanol-d_4) of heimionone B (**2**).

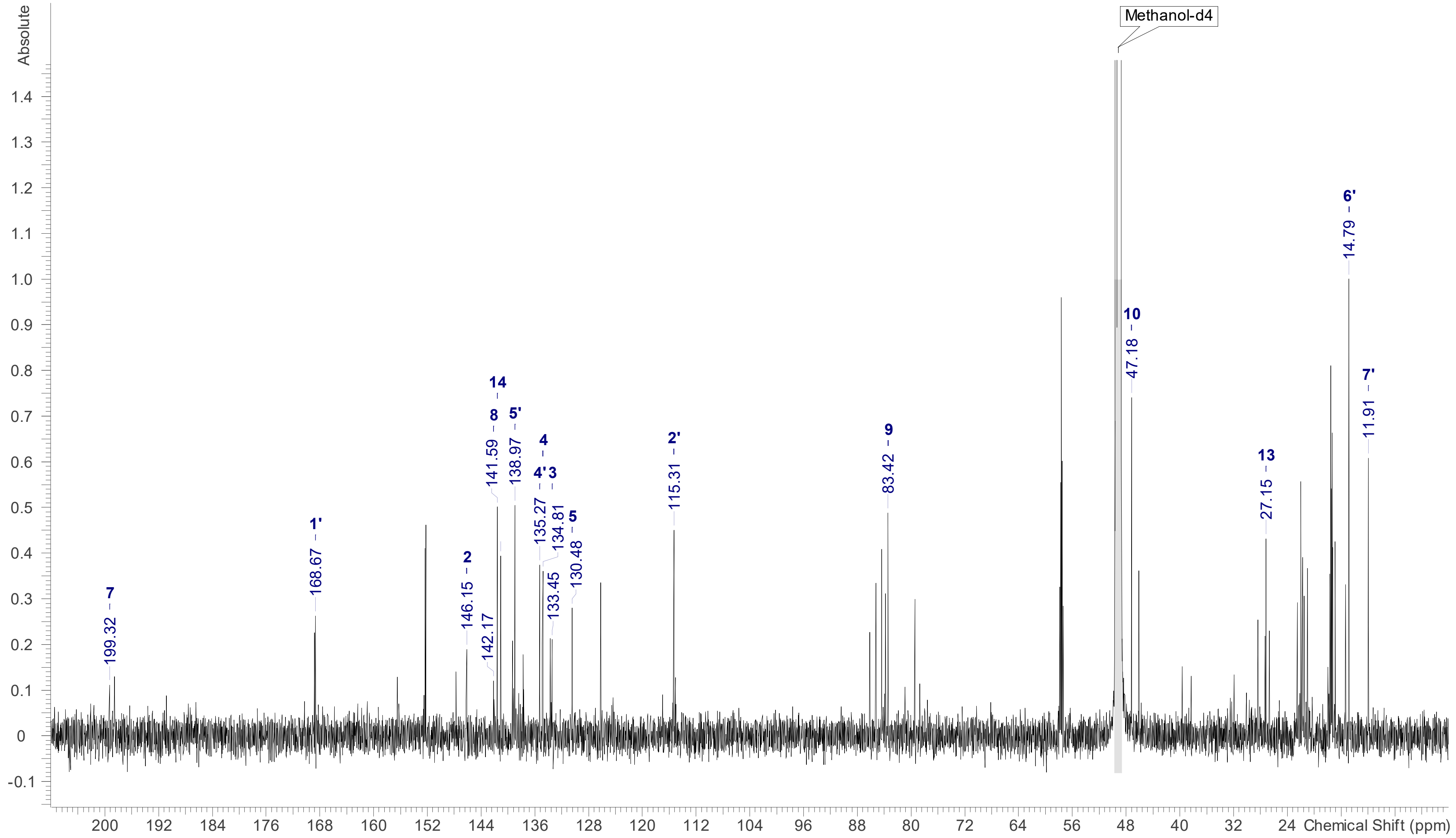

Figure S25. ^{13}C NMR spectrum (175 MHz, methanol-d_4) of heimionone B (**2**).

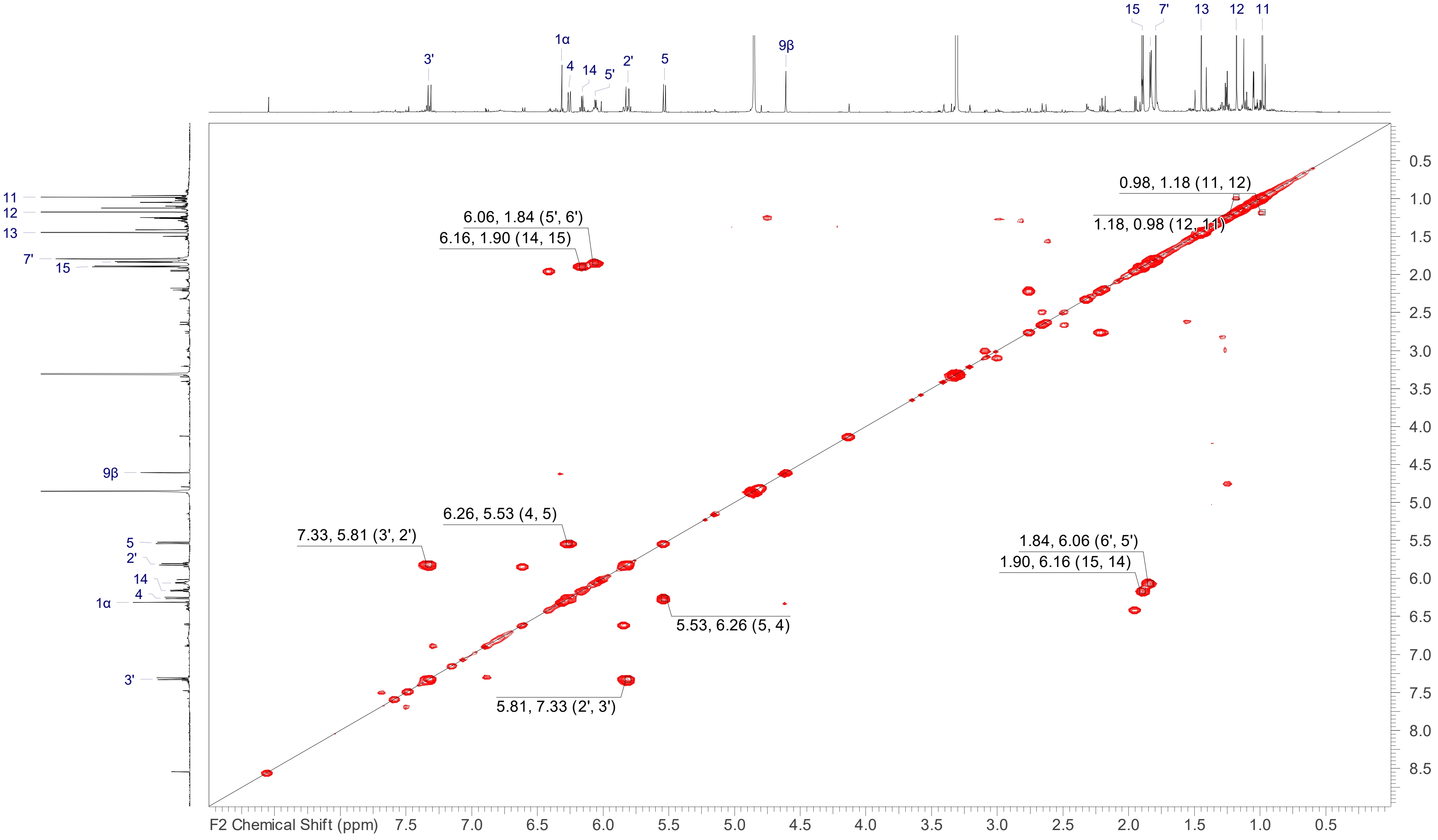

Figure S26. COSY NMR spectrum (700 MHz, methanol-d_4) of heimionone B (**2**).

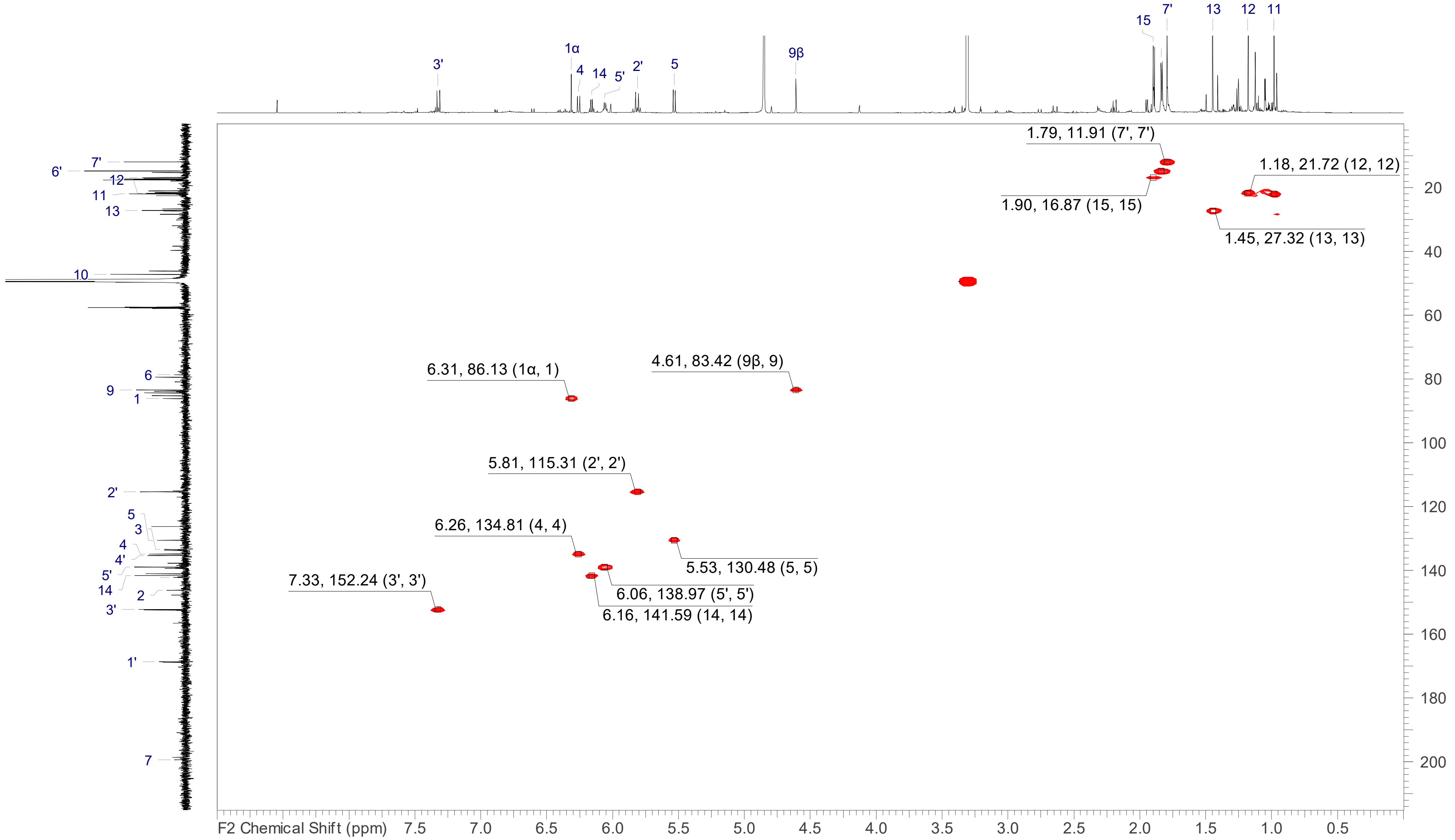

Figure S27. HSQC NMR spectrum (700 MHz, methanol-d_4) of heimionone B (**2**).

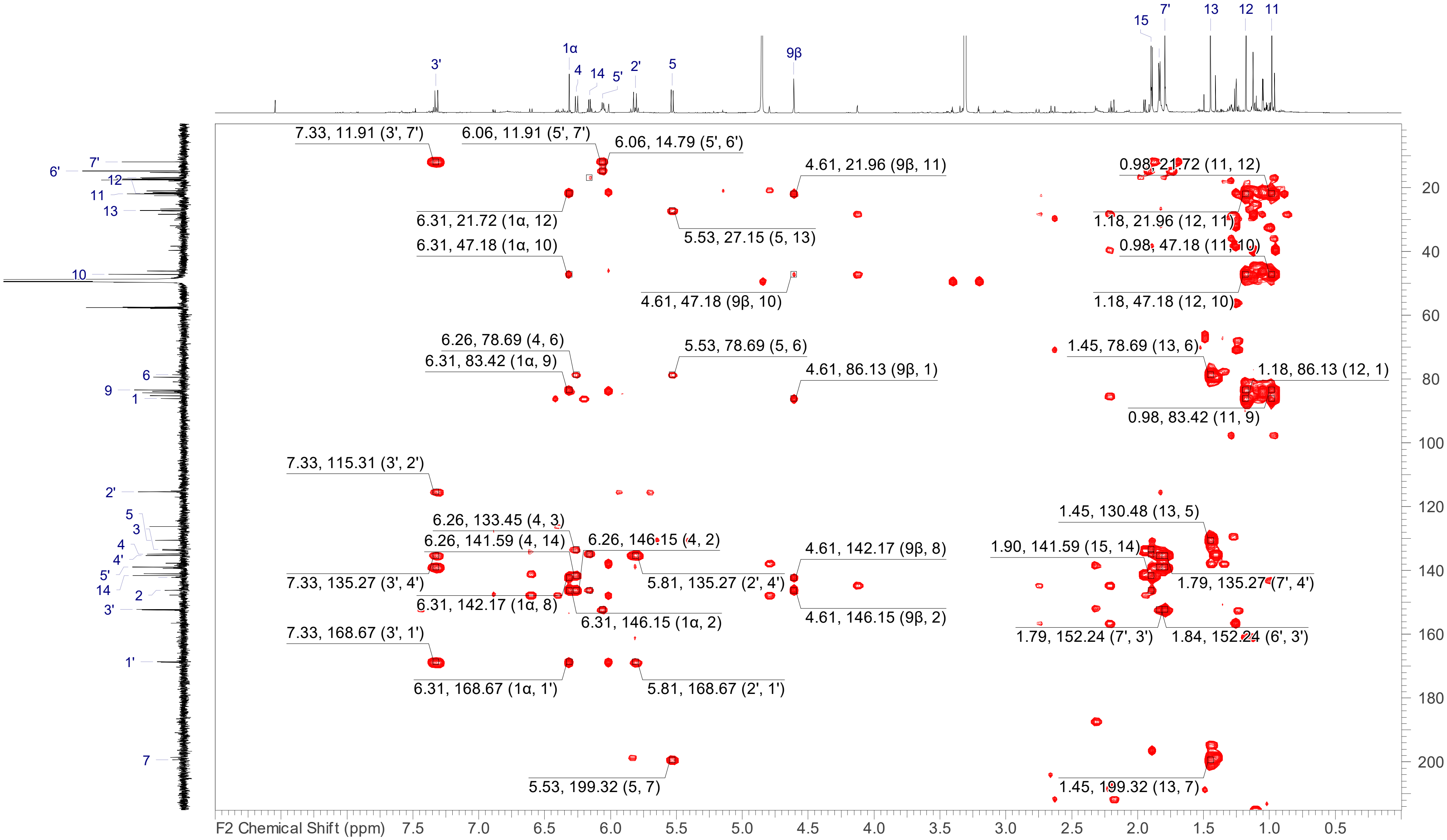

Figure S28. HMBC NMR spectrum (700 MHz, methanol-d_4) of heimionone B (**2**).

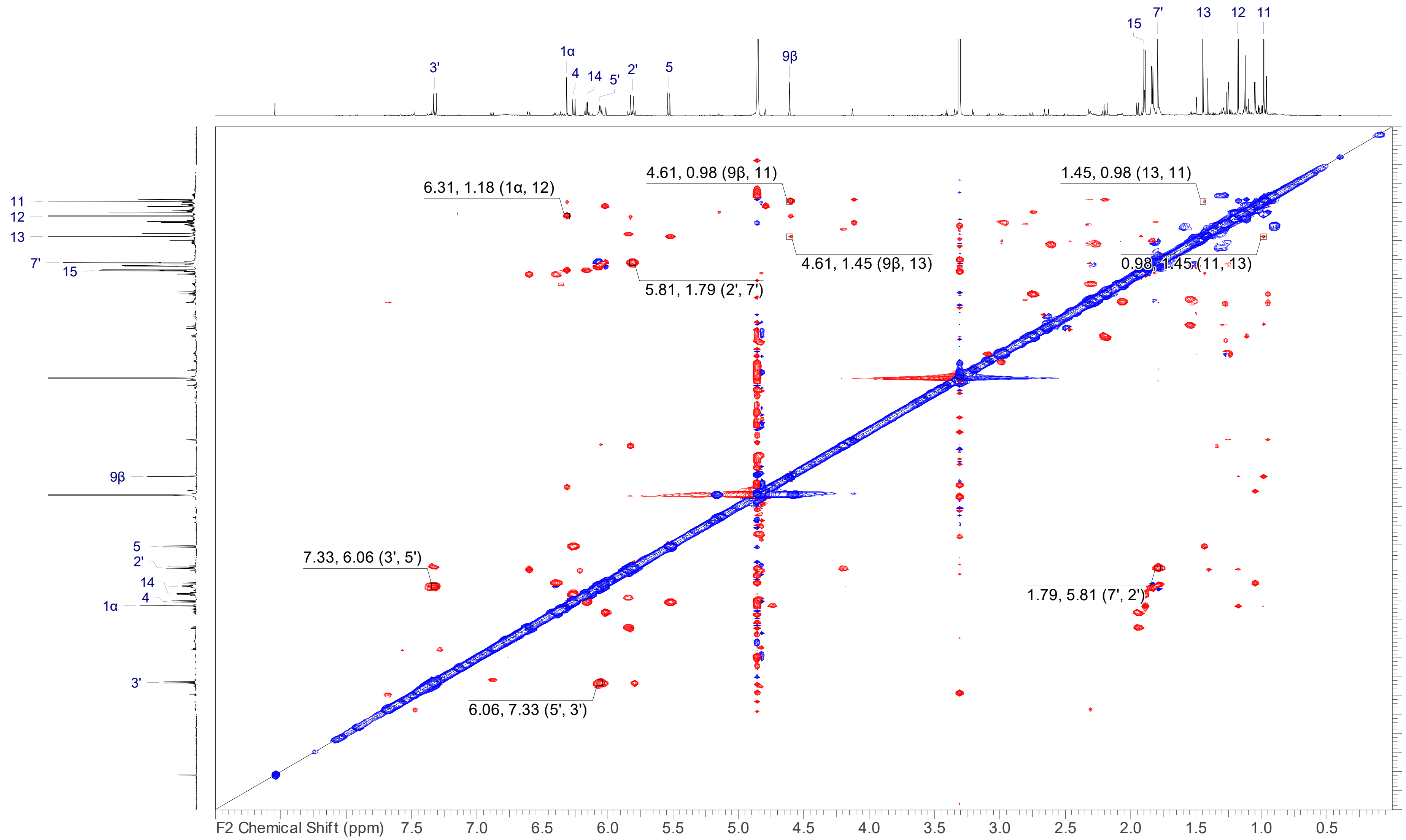

Figure S29. ROESY NMR spectrum (700 MHz, methanol-d_4) of heimionone B (**2**).

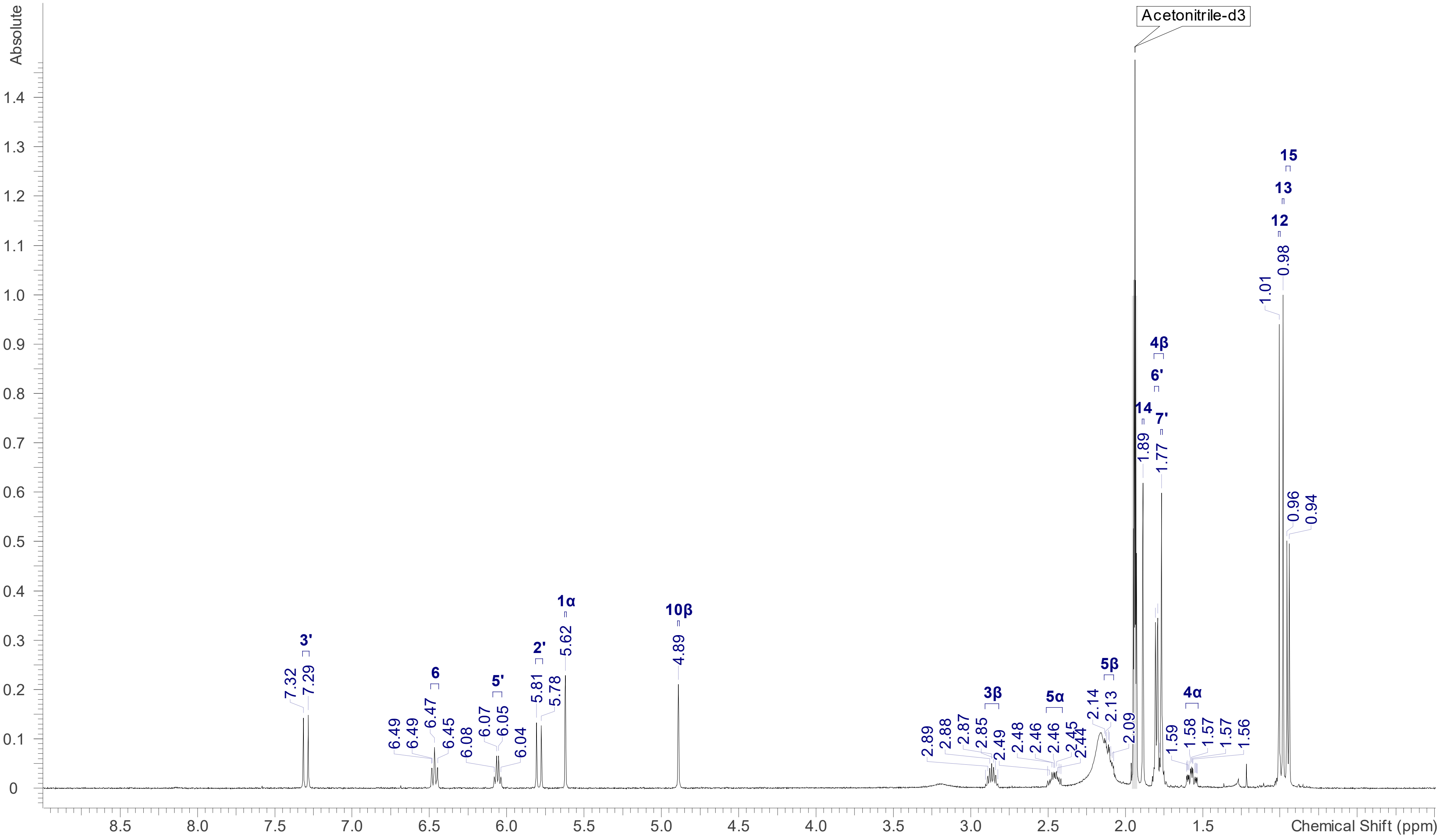

Figure S30. ^{1}H NMR spectrum (500 MHz, acetonitrile-*d*$_3$) of heimionone C (**3**).

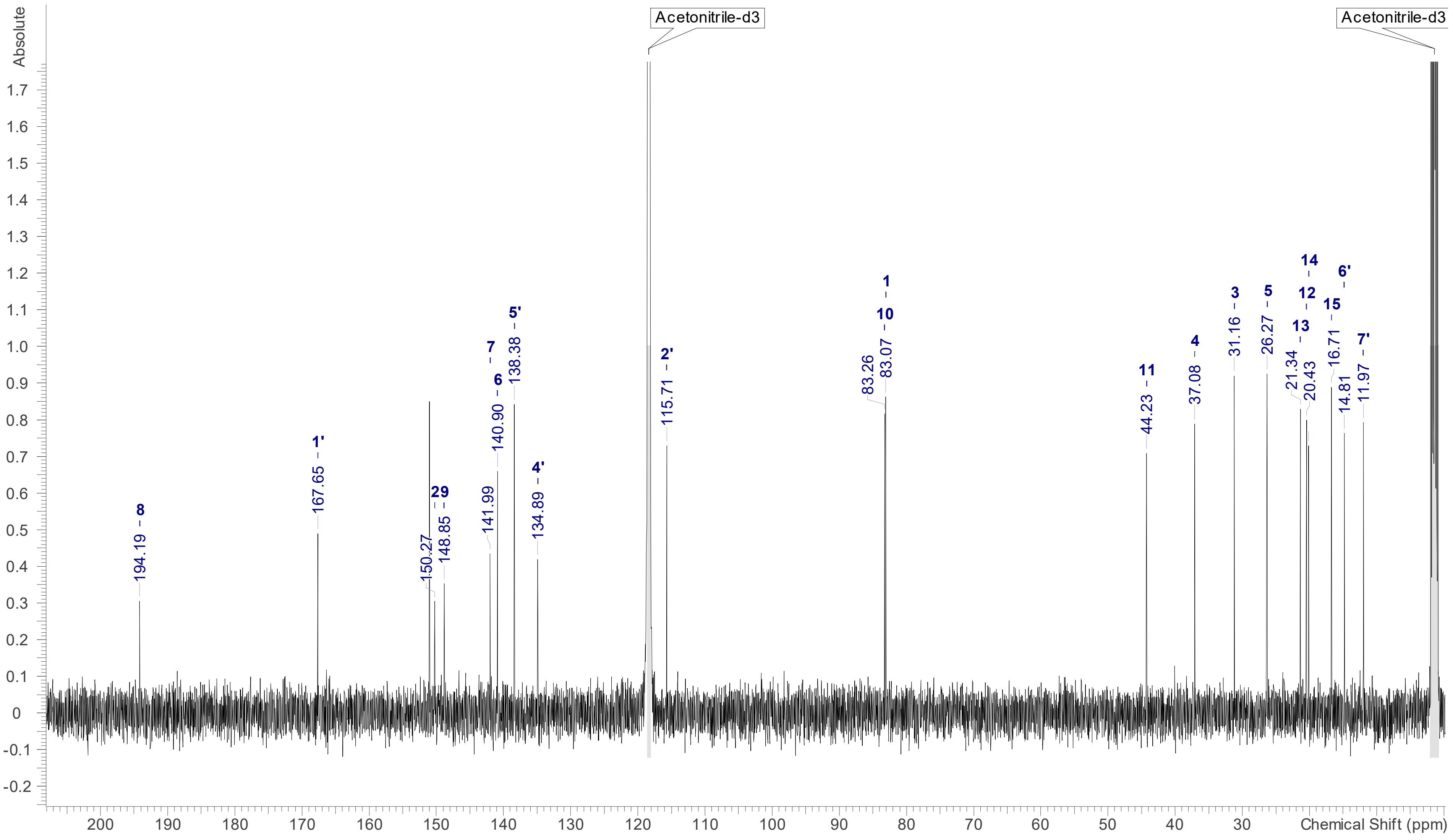

Figure S31. ^{13}C NMR spectrum (125 MHz, acetonitrile-d_3) of heimionone C (**3**).

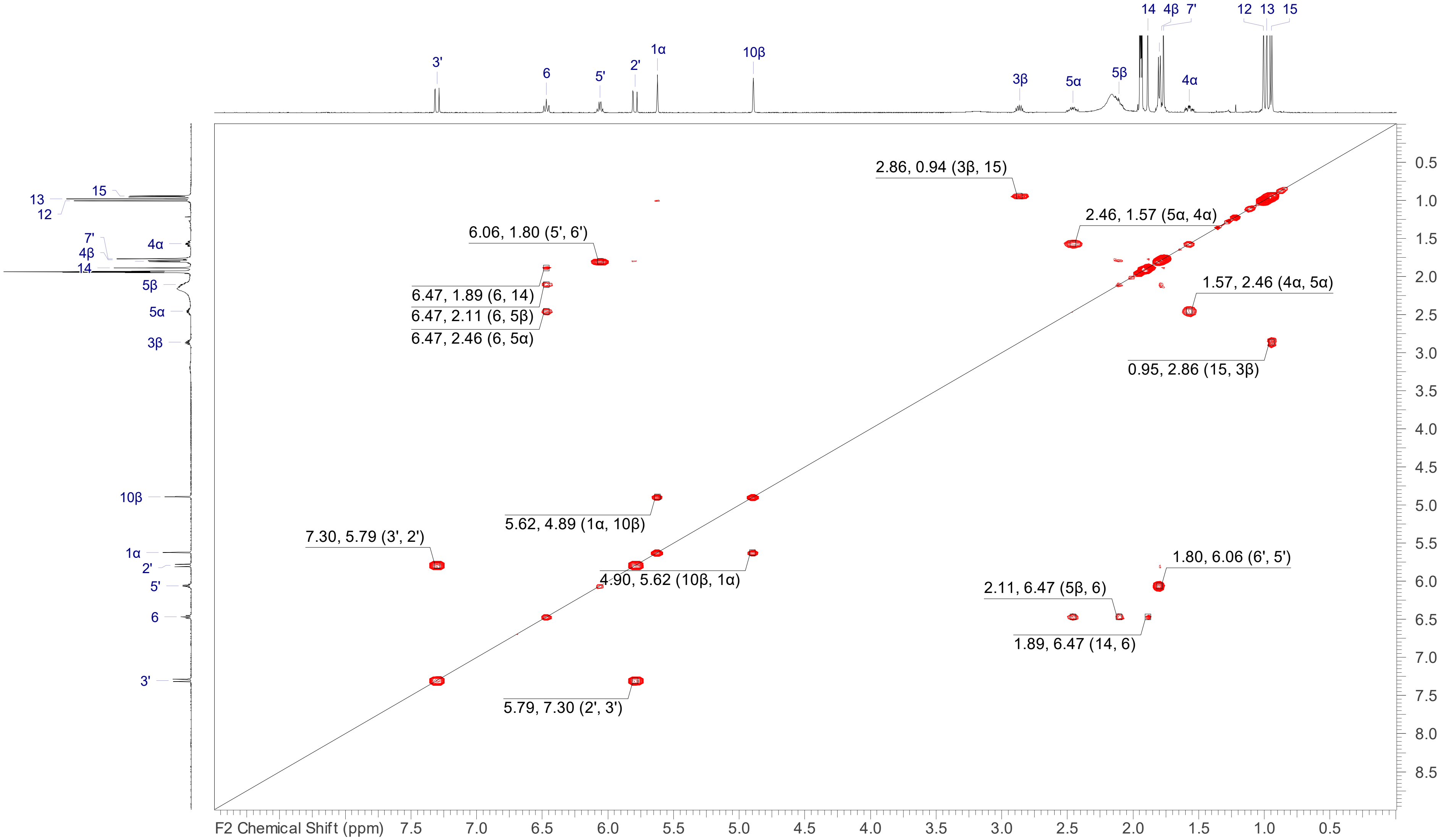

Figure S32. COSY NMR spectrum (500 MHz, acetonitrile-d_3) of heimionone C (**3**).

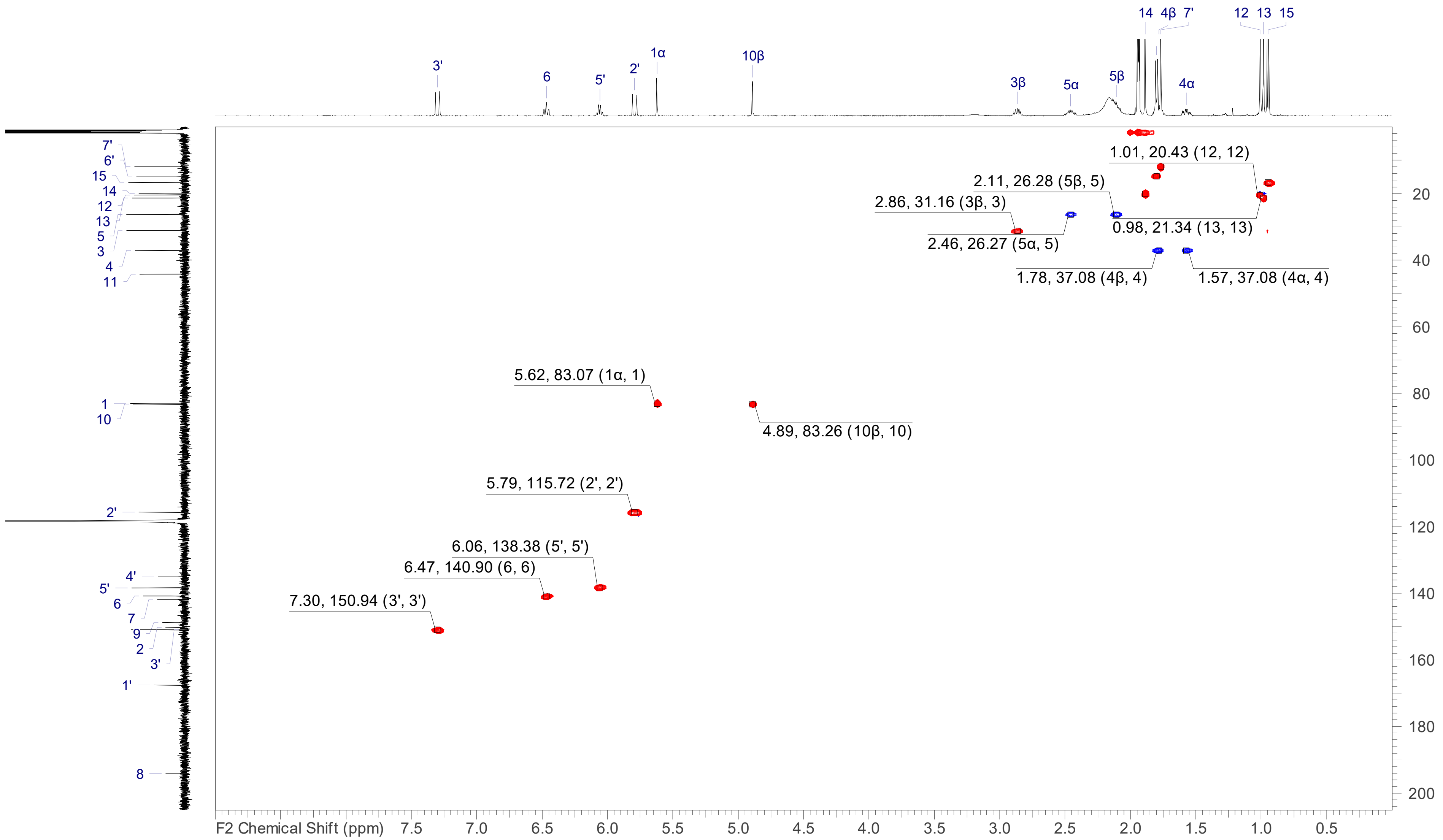

Figure S33. HSQC NMR spectrum (500 MHz, acetonitrile-d_3) of heimionone C (**3**).

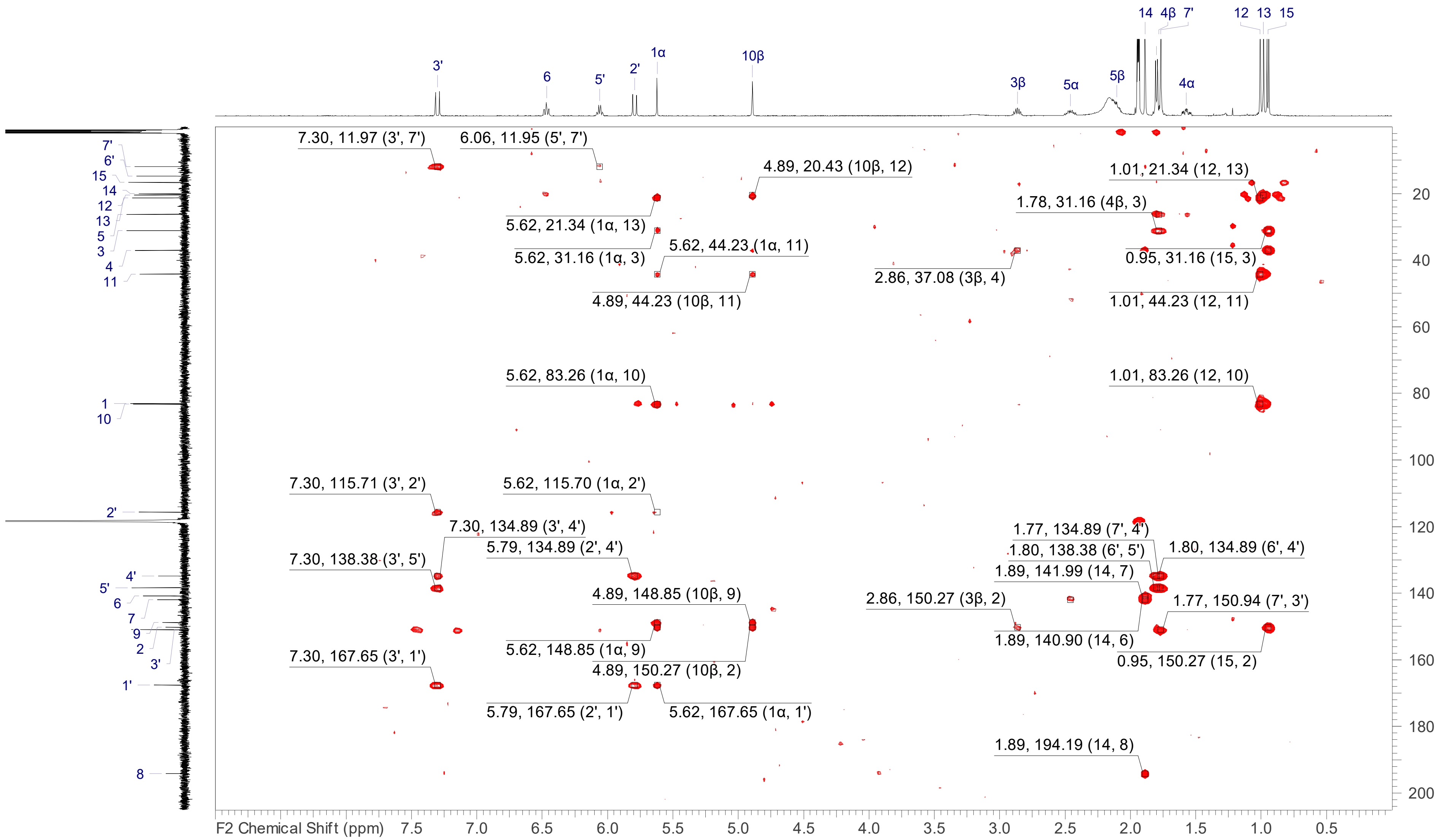

Figure S34. HMBC NMR spectrum (500 MHz, acetonitrile-*d*3) of heimionone C (**3**).

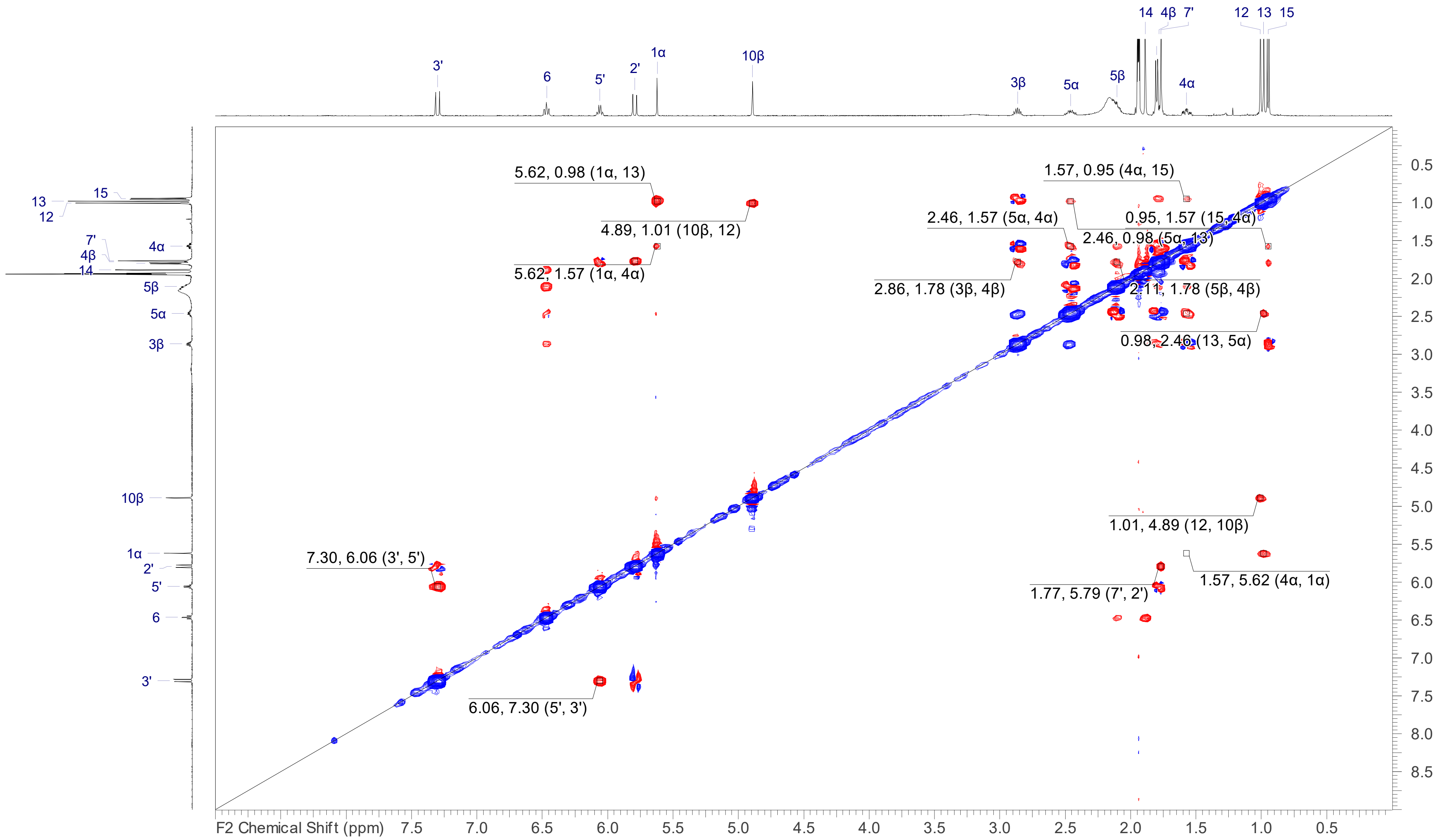

Figure S35. ROESY NMR spectrum (500 MHz, acetonitrile-d_3) of heimionone C (**3**).

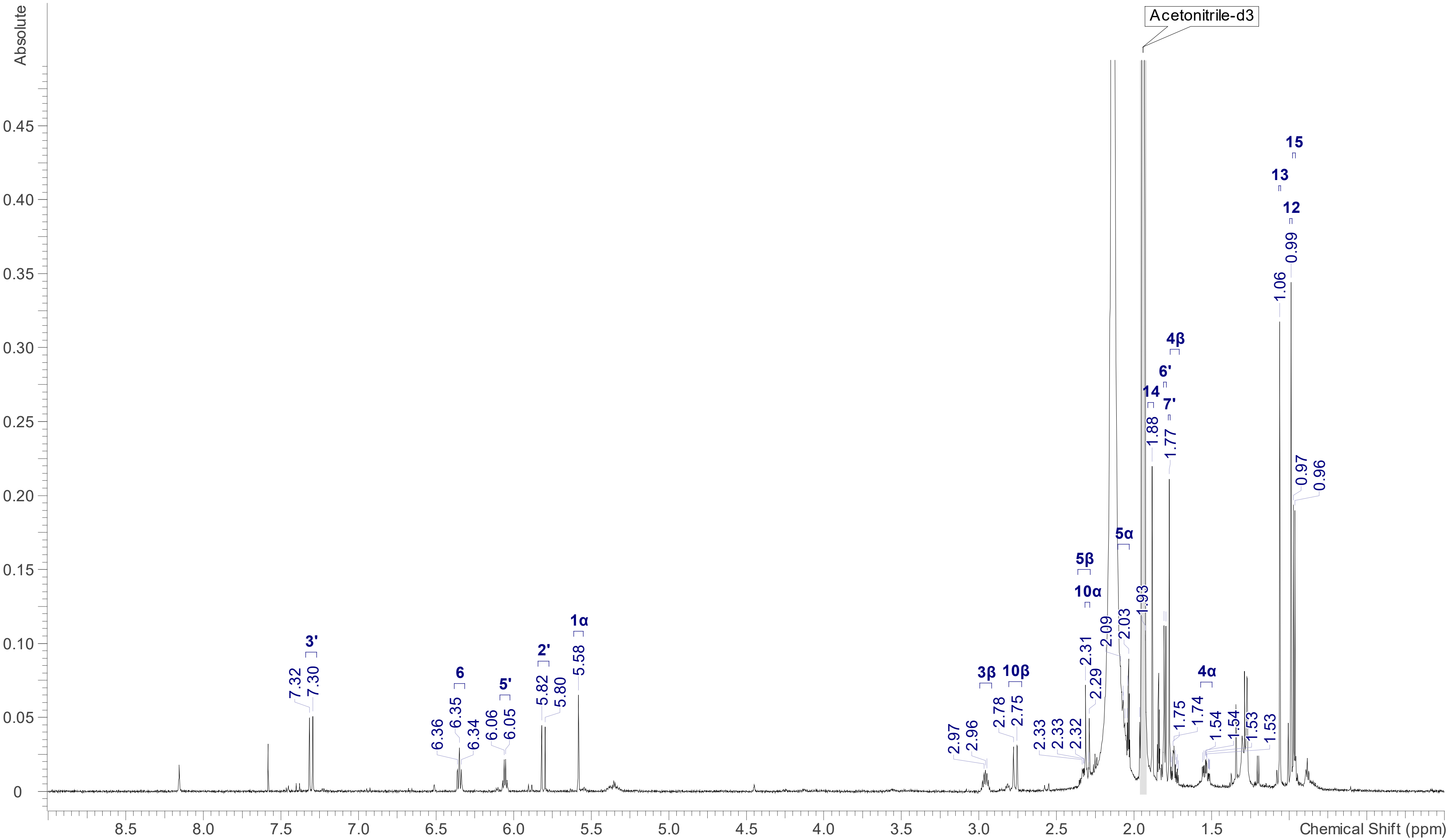

Figure S36. ^{1}H NMR spectrum (700 MHz, acetonitrile-d_3) of heimionone D (**4**).

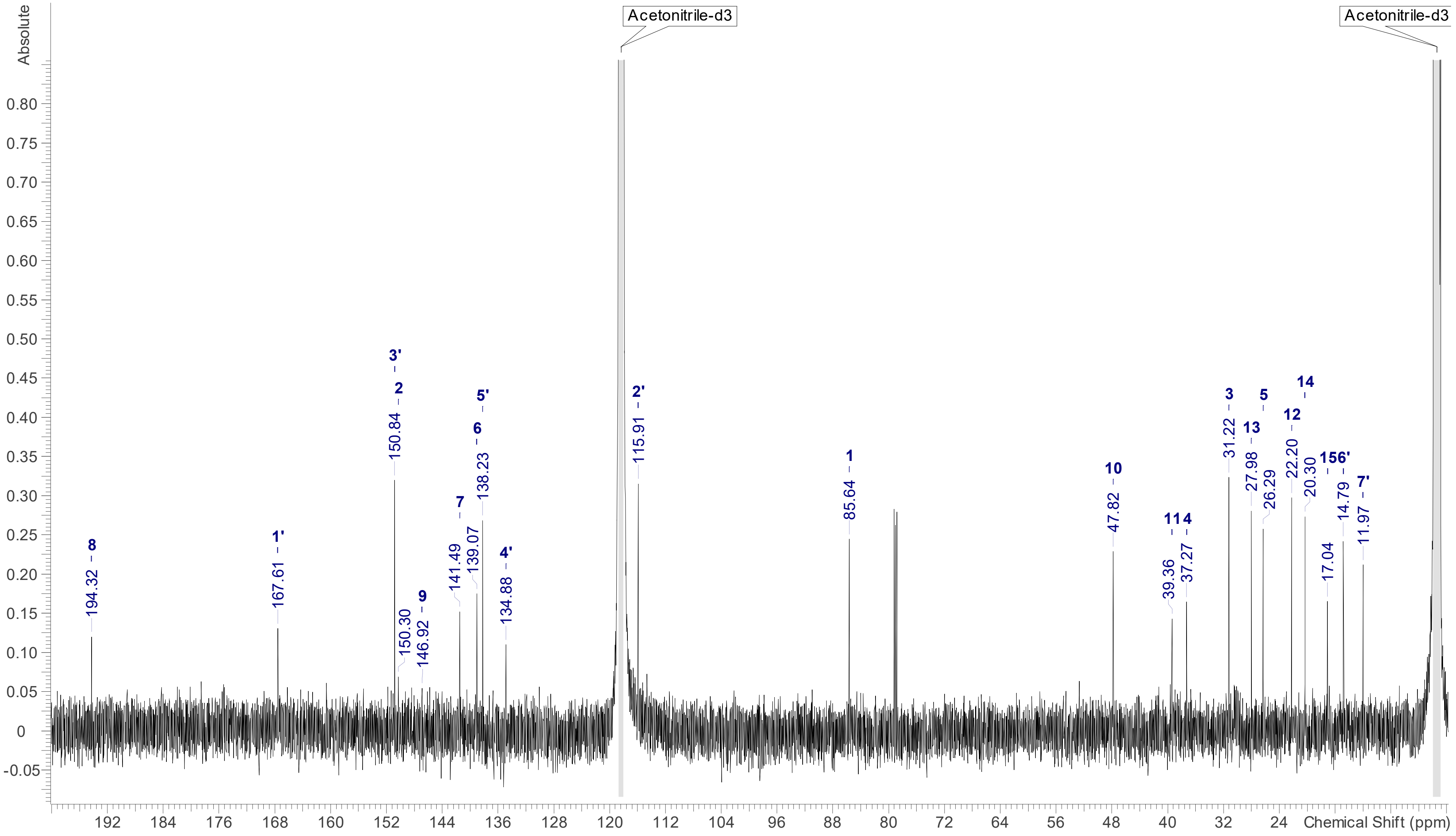

Figure S37. ^{13}C NMR spectrum (175 MHz, acetonitrile-d_3) of heimionone D (**4**).

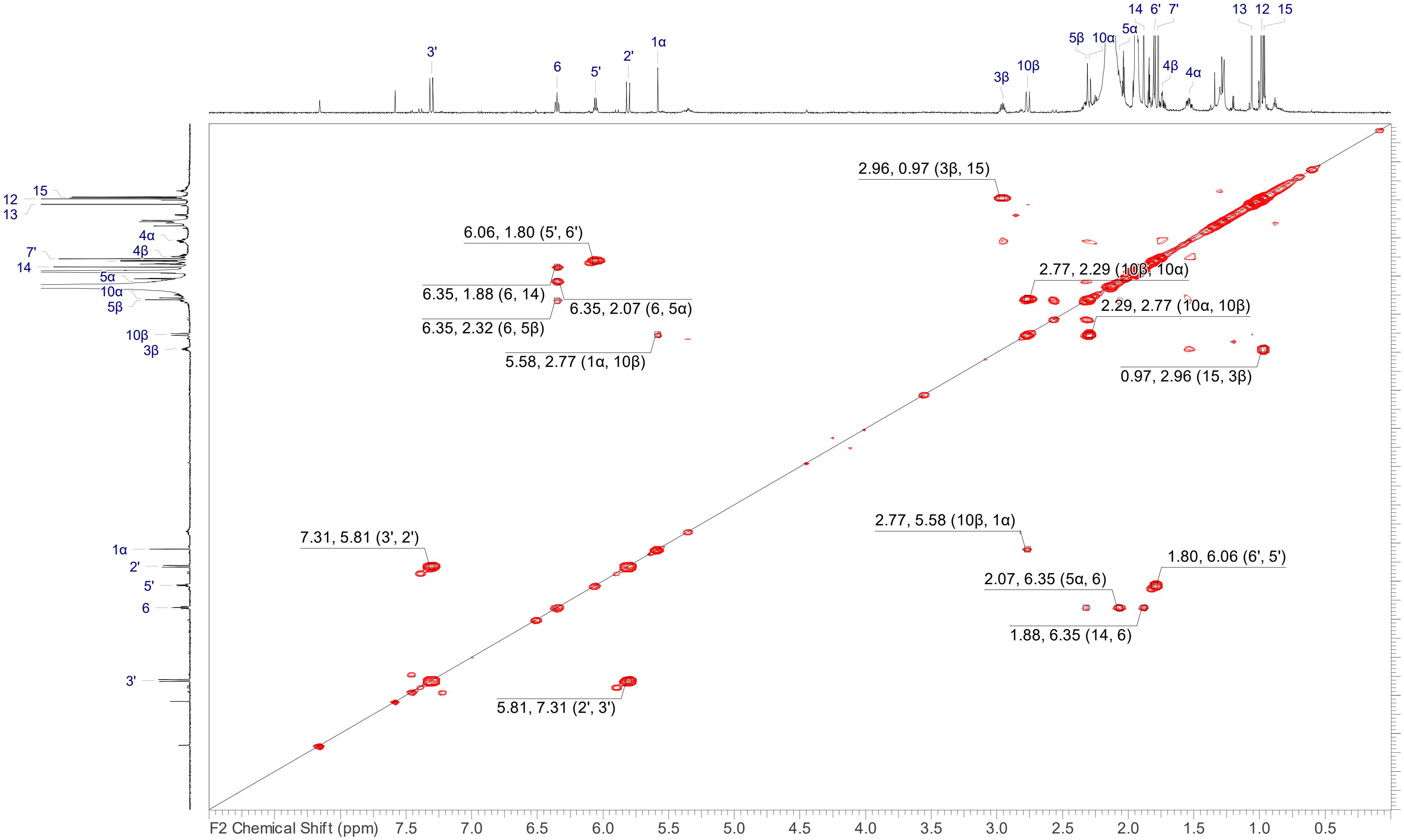

Figure S38. COSY NMR spectrum (700 MHz, acetonitrile-d_3) of heimionone D (**4**).

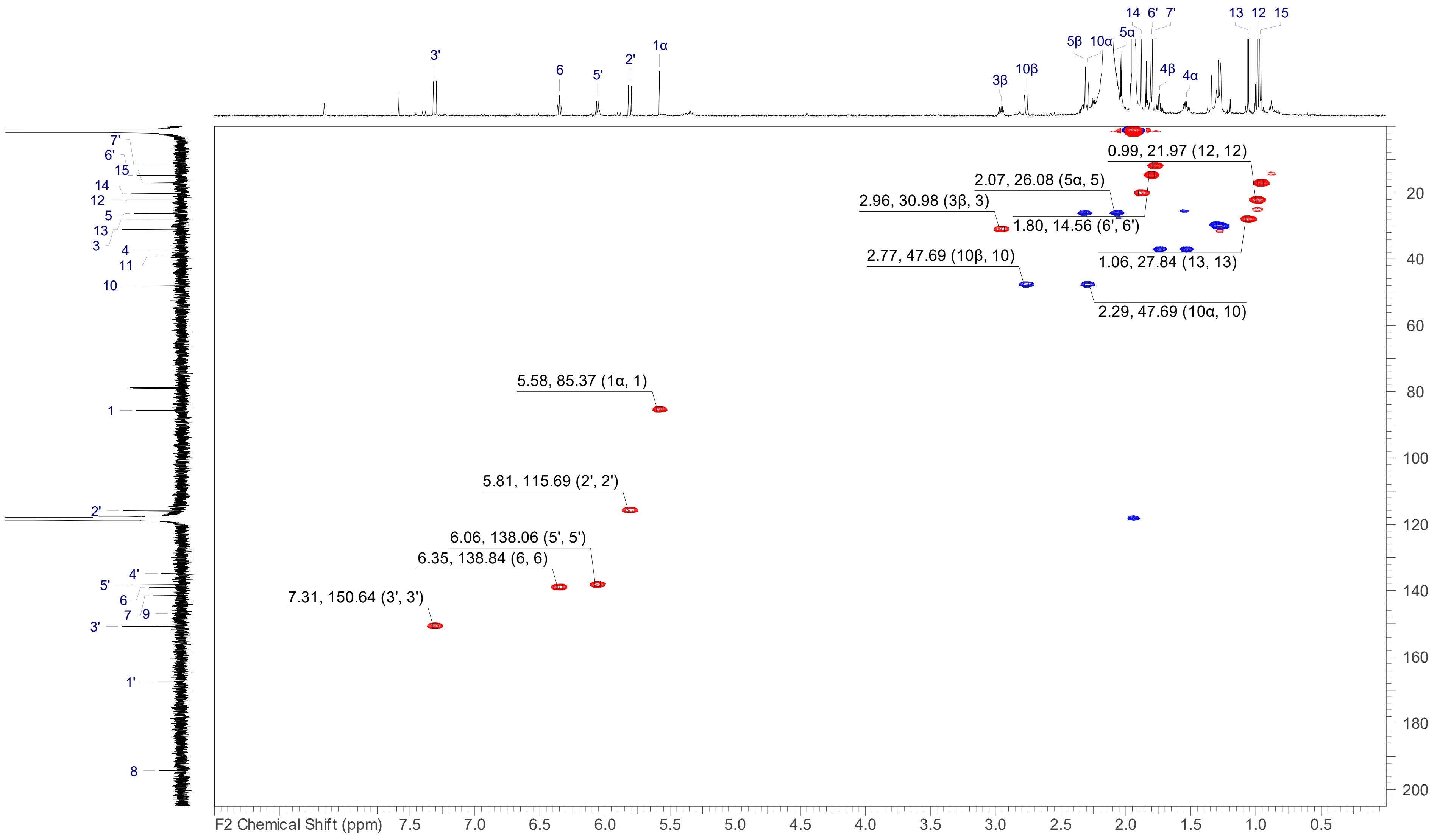

Figure S39. HSQC NMR spectrum (700 MHz, acetonitrile-d_3) of heimionone D (**4**).

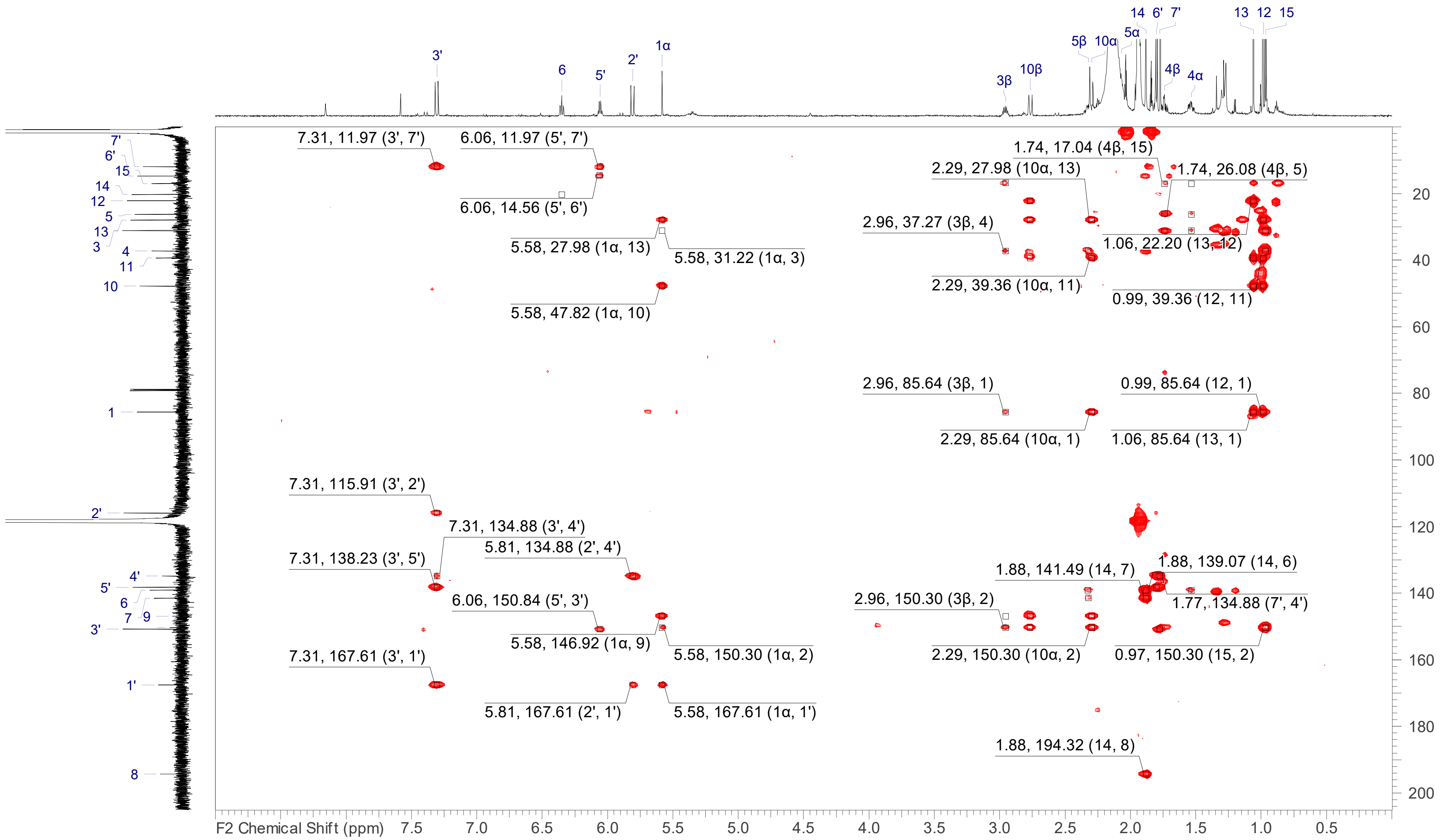

Figure S40. HMBC NMR spectrum (700 MHz, acetonitrile-d_3) of heimionone D (**4**).

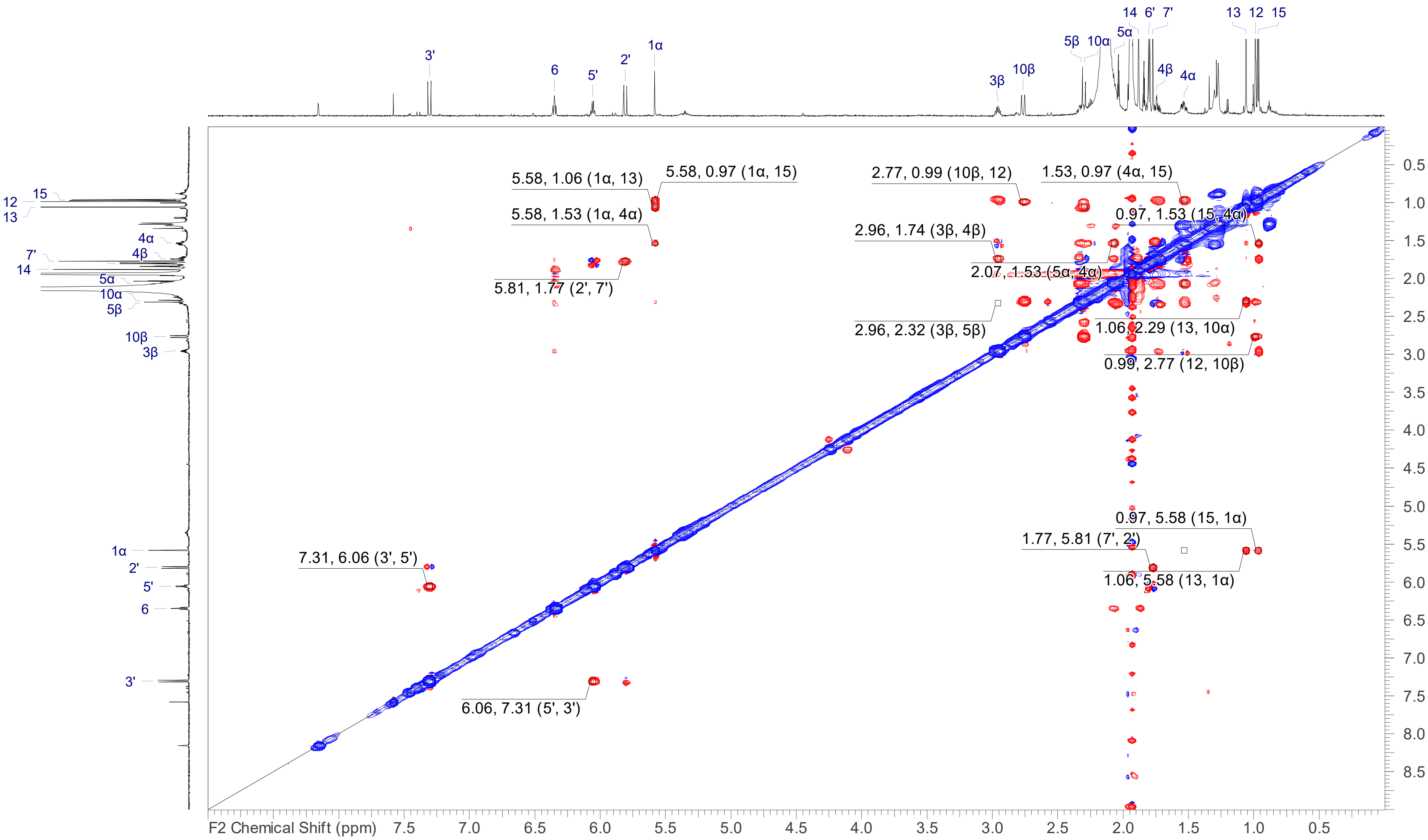

Figure S41. ROESY NMR spectrum (700 MHz, acetonitrile-d_3) of heimionone D (**4**).

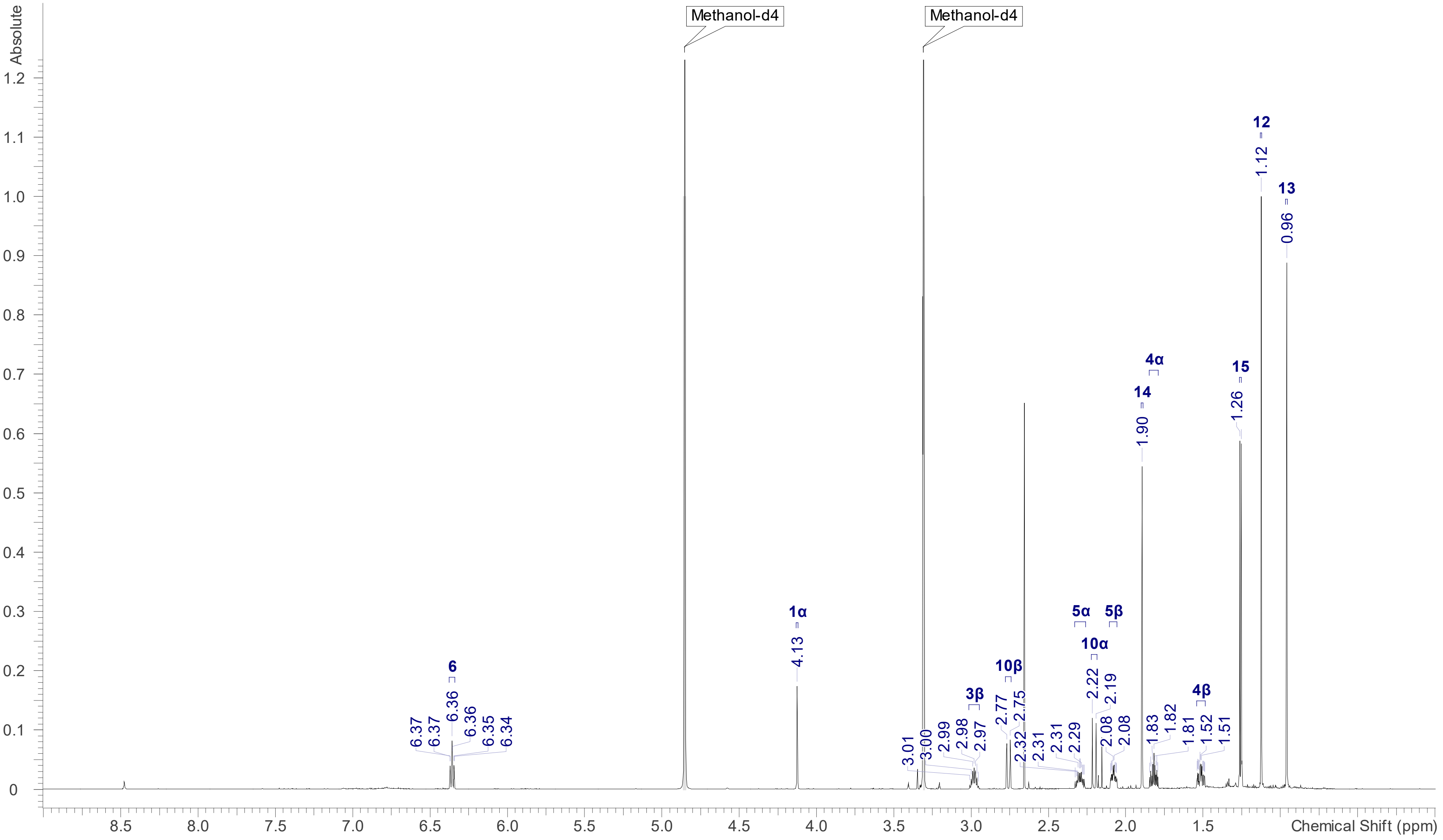

Figure S42. ^{1}H NMR spectrum (700 MHz, methanol-*d*$_4$) of heimionone E (**5**).

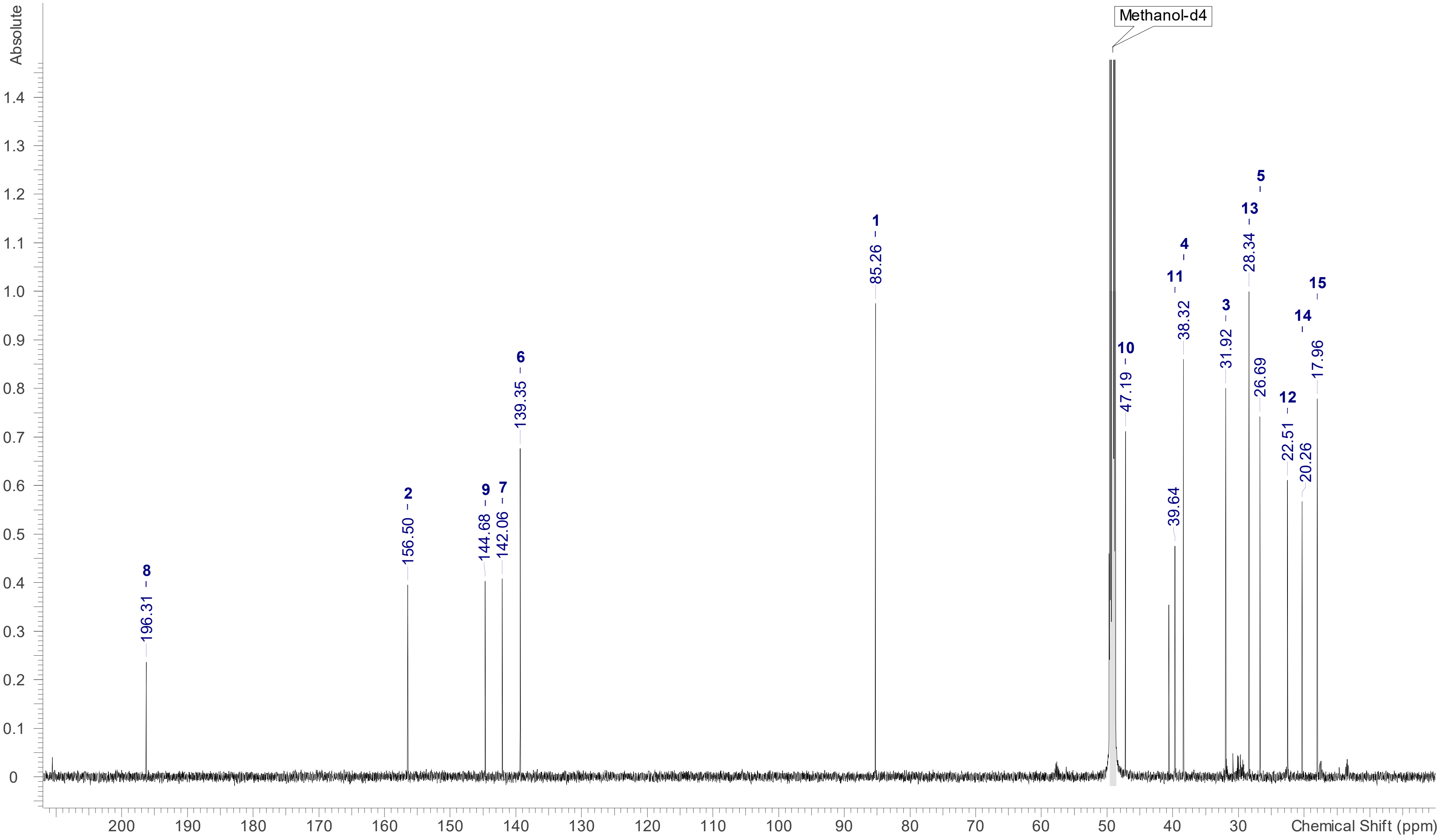

Figure S43. ^{13}C NMR spectrum (175 MHz, methanol-d_4) of heimionone E (**5**).

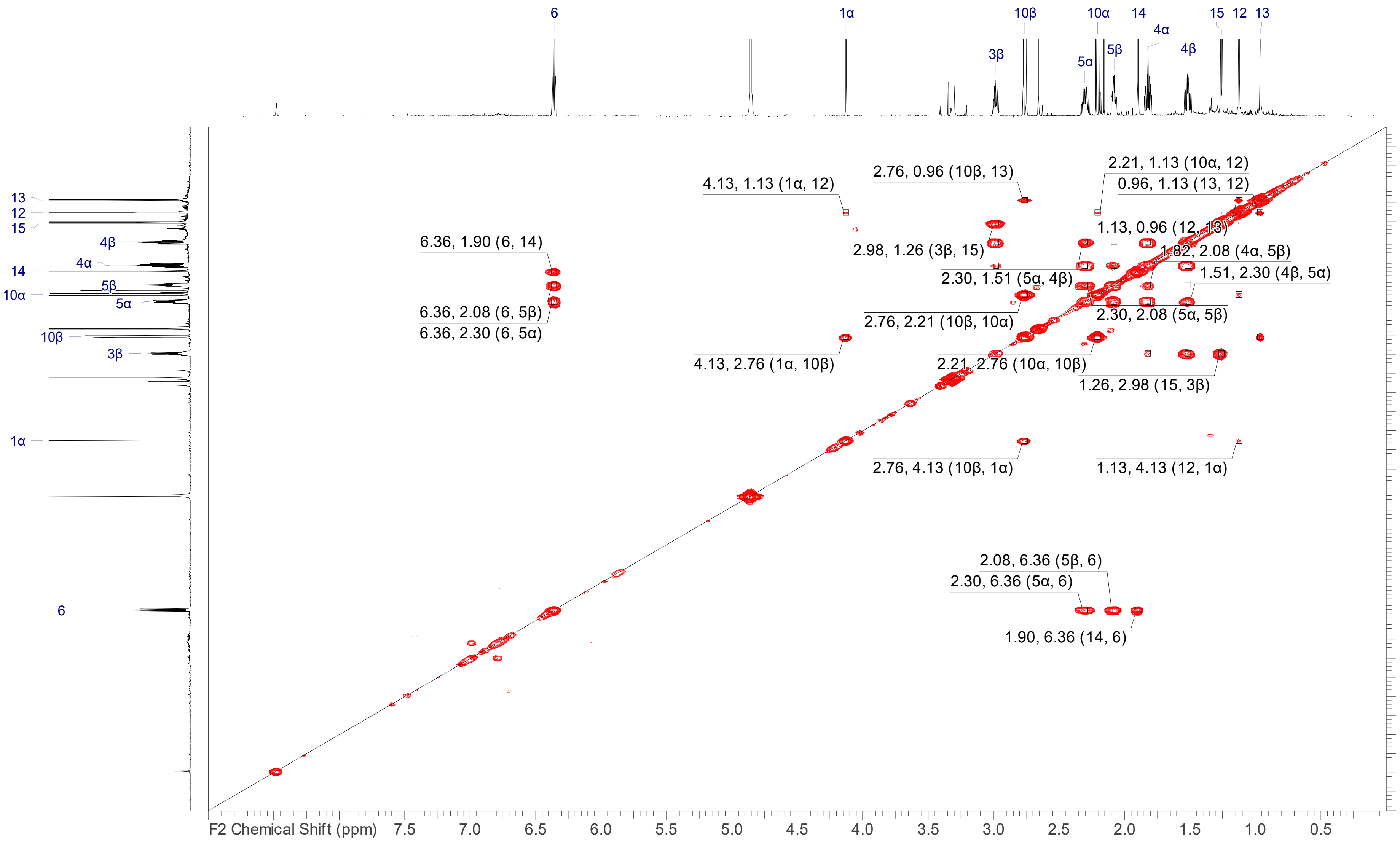

Figure S44. COSY NMR spectrum (700 MHz, methanol-d_4) of heimionone E (**5**).

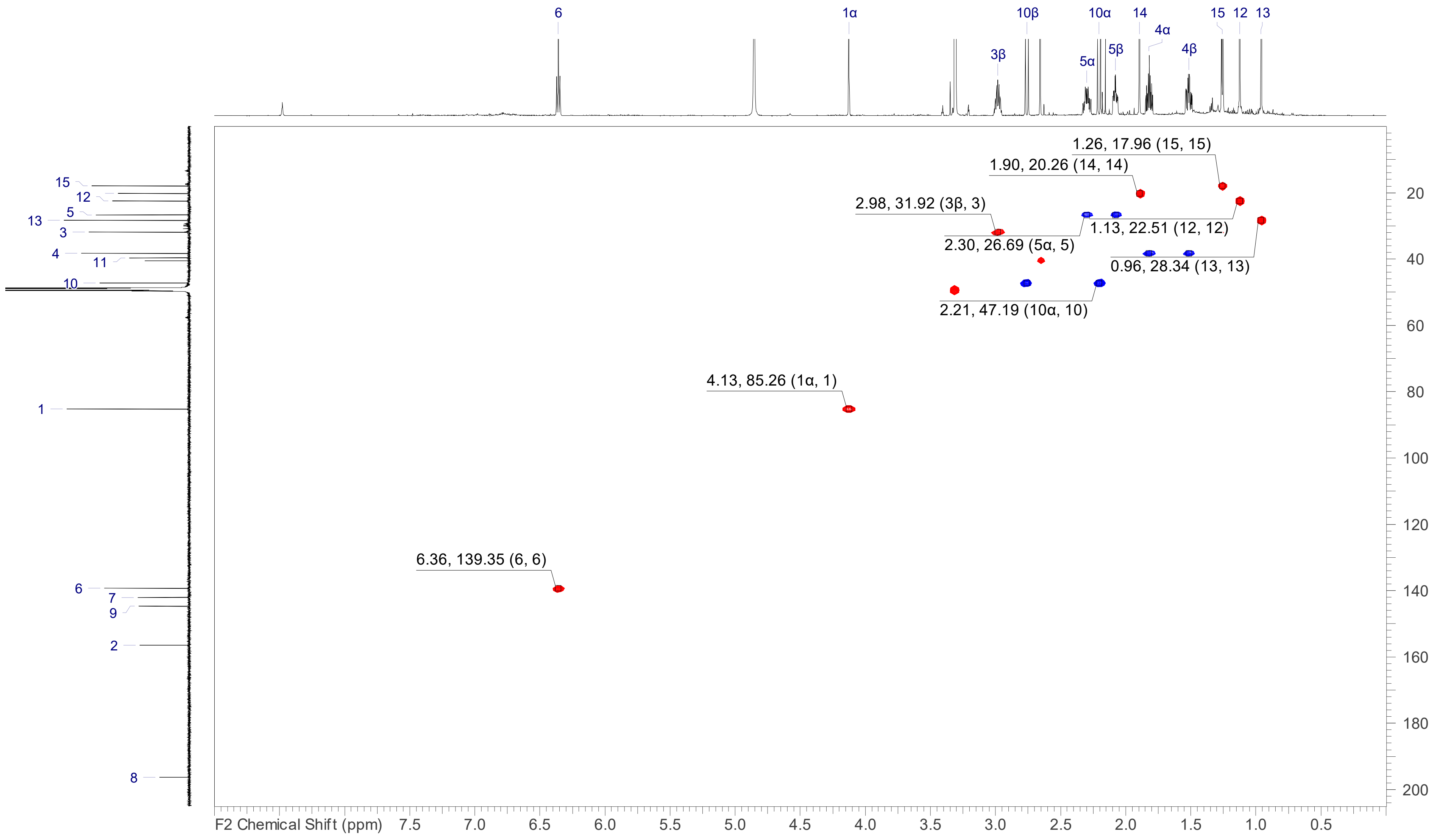

Figure S45. HSQC NMR spectrum (700 MHz, methanol-d_4) of heimionone E (**5**).

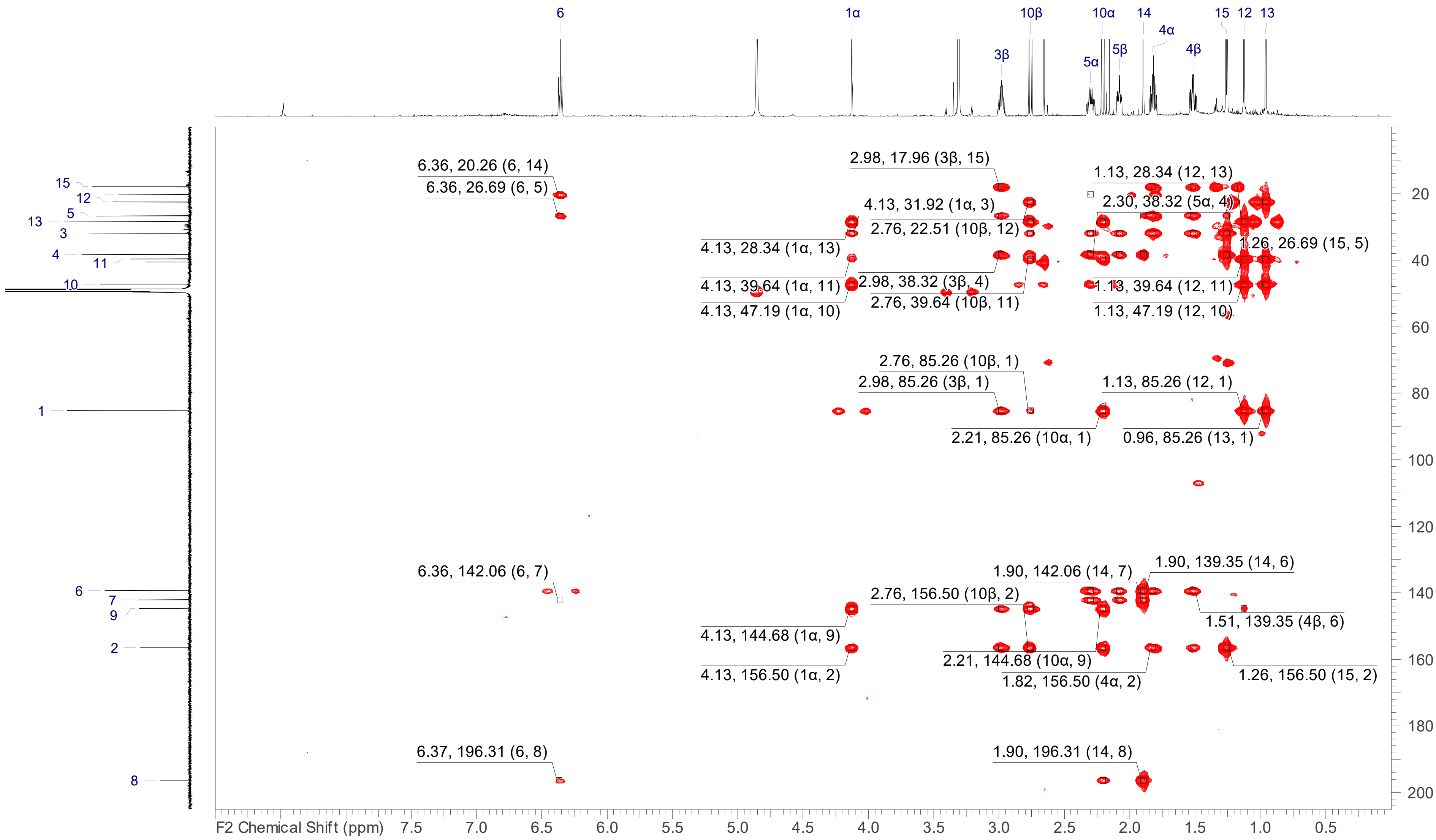

Figure S46. HMBC NMR spectrum (700 MHz, methanol-d_4) of heimionone E (**5**).

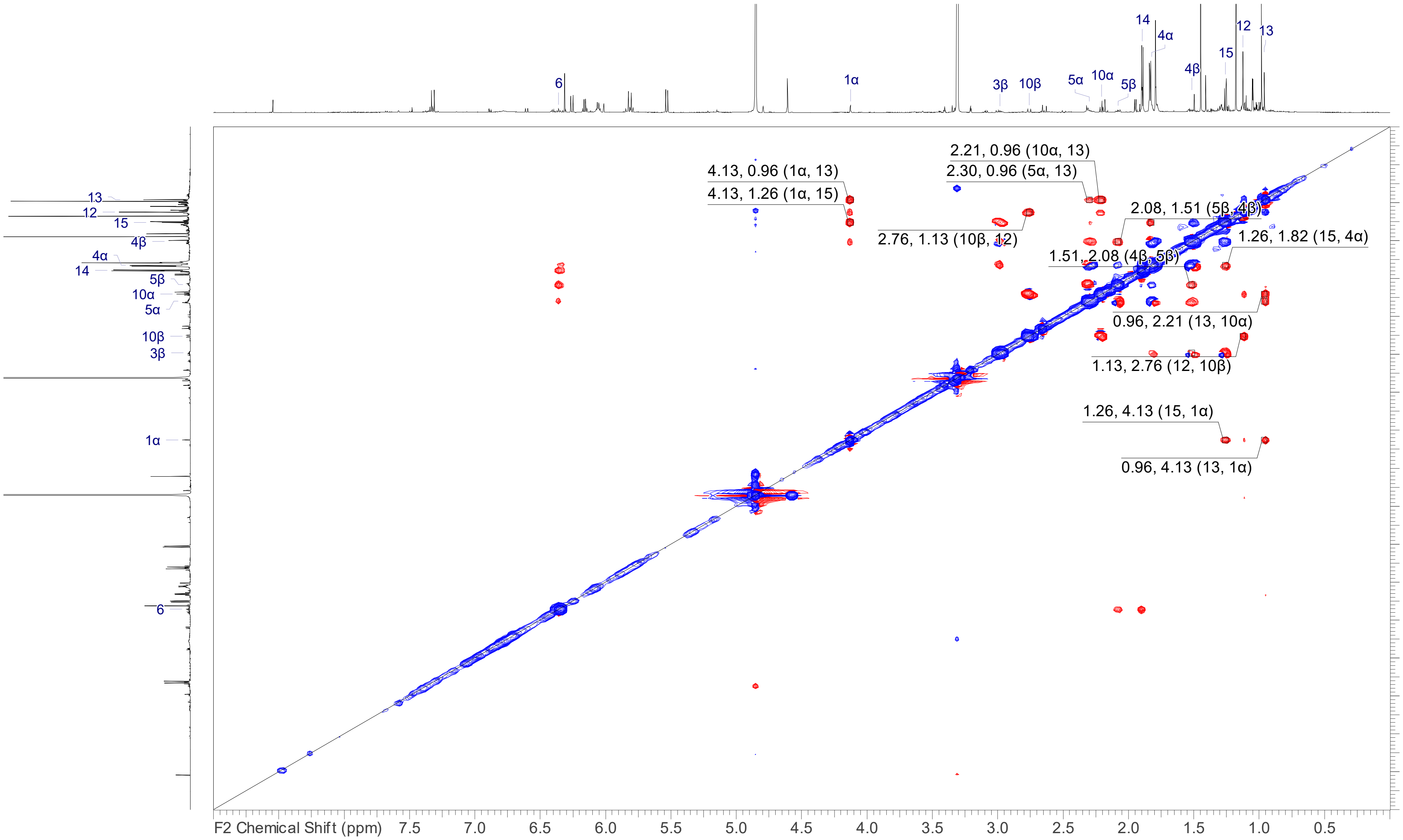

Figure S47. ROESY NMR spectrum (700 MHz, methanol-d_4) of heimionone E (**5**).
